# Supplementary material for: 5-Chloroisoxazoles: A Versatile Starting Material for the Preparation of Amides, Anhydrides, Esters, and Thioesters of 2H-Azirine-2-carboxylic Acids
Source: Molecules. 2022 Dec 29;28(1):275. doi: 10.3390/molecules28010275 (PMC9822487; doi:10.3390/molecules28010275)

Supporting Information for

5-Chloroisoxazoles: Versatile Starting Material for the Preparation of Amides, Anhydrides, Esters and Thioesters of 2*H*-Azirine-2-carboxylic Acids

Anastasiya V. Agafonova, Mikhail S. Novikov and Alexander F. Khlebnikov*

Saint Petersburg State University, Institute of Chemistry, 7/9 Universitetskaya Naberezhnaya, St. Petersburg 199034, Russia

e-mail: a.khlebnikov@spbu.ru

Table of Contents

1. X-ray diffraction of 7n (CCDC 2214535) 2
2. X-ray diffraction of 10a (CCDC 2215155) 7
3. References 11
4. NMR spectra of new compounds 12

1. X-ray diffraction of 7n (CCDC 2214535)

Single crystals of **7n** were grown by slow evaporation of hexane-diethyl ether solution at 4 °C. A suitable crystal was selected and studied on a XtaLAB Synergy, Single source at home/near, HyPix diffractometer. The crystal was kept at 99.9(8) K during data collection. Using Olex2 [1], the structure was solved with the SHELXT [2] structure solution program using Intrinsic Phasing and refined with the SHELXL [3] refinement package using Least Squares minimisation.

Figure S1. Molecular structure of **7n** (CCDC 2214535)

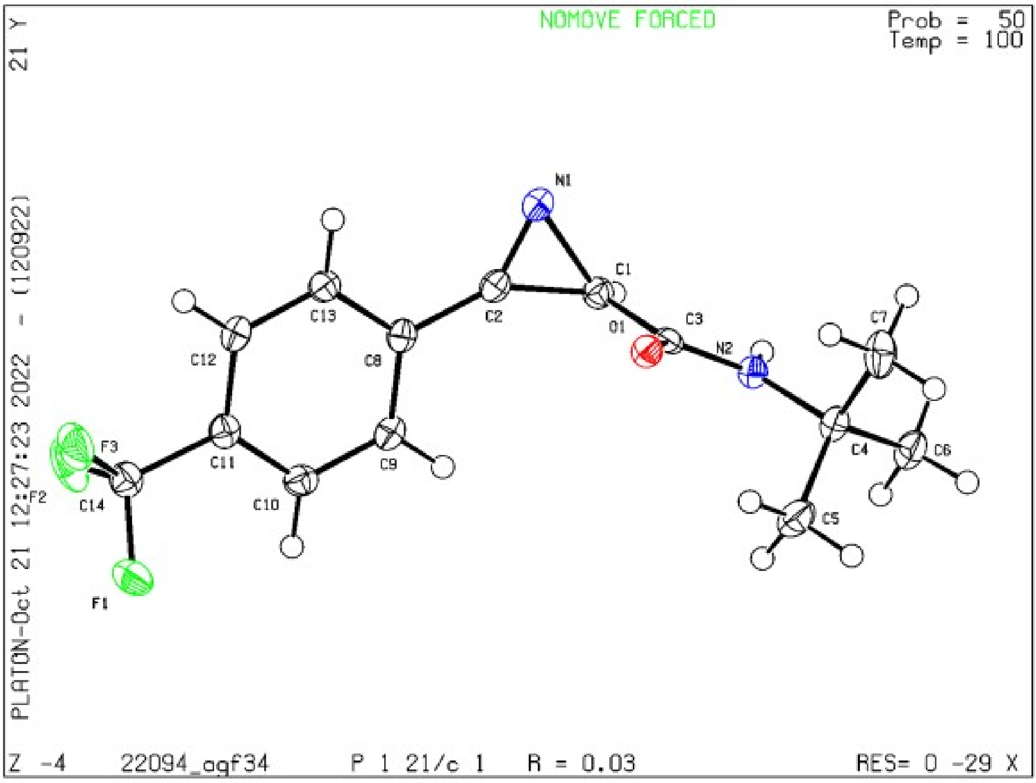

Table S1. Crystal data and structure refinement for **7n**.

Identification code	**7n**
Empirical formula	$C_{14}H_{15}N_2OF_3$
Formula weight	284.28
Temperature/K	99.9(8)
Crystal system	monoclinic
Space group	$P2_1/c$
a/Å	13.4802(2)
b/Å	11.28090(10)
c/Å	9.18210(10)
α/°	90
β/°	90.9830(10)
γ/°	90
Volume/Å^3	1396.11(3)

Z	4
ρ_{calc}g/cm^3	1.352
μ/mm^{-1}	0.978
F(000)	592.0
Crystal size/mm^3	0.12 × 0.1 × 0.08
Radiation	Cu Kα (λ = 1.54184)
2Θ range for data collection/°	6.558 to 124.98
Index ranges	-15 ≤ h ≤ 15, -12 ≤ k ≤ 12, -10 ≤ l ≤ 10
Reflections collected	15788
Independent reflections	2224 [R_{int} = 0.0402, R_{sigma} = 0.0266]
Data/restraints/parameters	2224/0/188
Goodness-of-fit on F^2	1.074
Final R indexes [I>=2σ (I)]	R_1 = 0.0302, wR_2 = 0.0759
Final R indexes [all data]	R_1 = 0.0329, wR_2 = 0.0777
Largest diff. peak/hole / e Å^{-3}	0.23/-0.24

Table S2. Fractional Atomic Coordinates (×10^4) and Equivalent Isotropic Displacement Parameters (Å^2×10^3) for **7n**. U_{eq} is defined as 1/3 of the trace of the orthogonalised U_{IJ} tensor.

**Atom**	***x***	***y***	***z***	**U(eq)**
F1	2227.2(6)	6968.8(7)	7051.7(9)	31.0(2)
O1	7350.0(6)	8141.5(7)	3878.0(9)	18.9(2)
F3	2020.9(7)	8850.1(8)	7103.5(10)	38.4(2)
F2	1356.9(6)	7819.5(10)	5381.4(9)	42.2(3)
N2	7826.0(8)	6909.9(9)	2040.7(12)	16.8(2)
N1	5978.2(8)	9331.9(9)	1849.4(12)	22.5(3)
C3	7225.3(9)	7703.2(10)	2653.7(13)	15.7(3)
C2	5489.7(9)	8546.5(11)	2491.0(13)	18.2(3)
C9	4370.7(9)	7253.3(11)	3867.8(14)	19.9(3)
C8	4650.9(9)	8389.5(11)	3444.6(13)	17.9(3)
C11	3077.3(9)	8092.2(11)	5295.8(13)	19.0(3)
C12	3359.0(9)	9232.9(11)	4894.9(14)	19.3(3)
C13	4141.0(9)	9376.8(11)	3956.0(14)	19.1(3)
C4	8753.5(9)	6425.8(11)	2697.4(14)	20.2(3)
C10	3577.2(9)	7104.1(11)	4784.7(14)	20.7(3)
C1	6326.9(9)	8015.2(11)	1753.3(14)	18.6(3)
C14	2177.4(10)	7935.7(12)	6212.3(14)	22.6(3)
C6	9142.8(10)	5504.3(12)	1631.8(15)	25.2(3)
C5	8526.6(11)	5823.2(13)	4145.8(15)	28.7(3)
C7	9509.5(10)	7425.1(13)	2896.9(16)	29.6(3)

Table S3. Anisotropic Displacement Parameters ($Å^2 \times 10^3$) for **7n**. The Anisotropic displacement factor exponent takes the form: $-2\pi^2[h^2a^{*2}U_{11}+2hka^*b^*U_{12}+\ldots]$.

Atom	U_{11}	U_{22}	U_{33}	U_{23}	U_{13}	U_{12}
F1	30.9(4)	33.7(5)	28.6(4)	8.2(3)	5.8(3)	-3.1(3)
O1	21.2(5)	20.5(4)	15.1(4)	-1.2(3)	1.2(3)	2.4(3)
F3	38.0(5)	35.1(5)	42.8(5)	-7.5(4)	21.4(4)	-0.2(4)
F2	16.5(4)	81.0(7)	29.0(5)	9.6(4)	-2.3(3)	-7.5(4)
N2	17.4(5)	19.6(5)	13.4(6)	-2.0(4)	0.1(4)	0.7(4)
N1	20.7(6)	21.8(6)	25.1(6)	2.7(4)	1.5(4)	4.6(4)
C3	16.7(6)	14.9(6)	15.7(6)	2.2(5)	4.0(5)	-2.3(5)
C2	17.6(6)	19.1(6)	17.8(6)	-1.2(5)	-3.0(5)	2.0(5)
C9	18.5(6)	18.5(6)	22.5(6)	-2.7(5)	-1.6(5)	2.9(5)
C8	15.0(6)	21.2(6)	17.4(6)	-1.2(5)	-3.0(5)	0.7(5)
C11	15.5(6)	24.3(7)	17.2(6)	-0.6(5)	-3.7(5)	-0.4(5)
C12	17.0(6)	20.2(6)	20.8(6)	-3.9(5)	-2.3(5)	2.7(5)
C13	17.6(6)	17.2(6)	22.4(6)	0.0(5)	-2.0(5)	-0.9(5)
C4	17.5(6)	22.9(7)	20.1(6)	-1.5(5)	-0.1(5)	4.9(5)
C10	20.2(6)	18.7(6)	23.0(7)	1.6(5)	-3.0(5)	-1.6(5)
C1	18.9(6)	19.4(6)	17.4(6)	-0.4(5)	1.2(5)	1.1(5)
C14	19.2(7)	27.7(7)	20.7(6)	1.6(5)	-1.6(5)	-0.1(5)
C6	24.5(7)	26.6(7)	24.7(7)	-2.2(6)	1.2(5)	8.9(6)
C5	34.4(8)	29.6(7)	22.1(7)	2.6(6)	1.3(6)	12.8(6)
C7	17.6(7)	34.6(8)	36.5(8)	-10.0(6)	-0.6(6)	0.4(6)

Table S4. Bond Lengths for **7n**.

Atom	Atom	Length/Å	Atom	Atom	Length/Å
F1	C14	1.3366(16)	C9	C8	1.3934(18)
O1	C3	1.2370(15)	C9	C10	1.3827(19)
F3	C14	1.3357(16)	C8	C13	1.3945(18)
F2	C14	1.3393(16)	C11	C12	1.3930(18)
N2	C3	1.3376(16)	C11	C10	1.3883(18)
N2	C4	1.4830(16)	C11	C14	1.4986(18)
N1	C2	1.2566(17)	C12	C13	1.3828(19)
N1	C1	1.5608(16)	C4	C6	1.5268(18)
C3	C1	1.4965(18)	C4	C5	1.5292(19)
C2	C8	1.4528(18)	C4	C7	1.5286(19)
C2	C1	1.4553(18)			

Table S5. Bond Angles for **7n**.

Atom	Atom	Atom	Angle/°	Atom	Atom	Atom	Angle/°
C3	N2	C4	125.99(11)	N2	C4	C6	106.67(10)
C2	N1	C1	61.07(8)	N2	C4	C5	109.72(10)
O1	C3	N2	125.17(11)	N2	C4	C7	109.44(10)
O1	C3	C1	120.28(11)	C6	C4	C5	109.31(11)
N2	C3	C1	114.52(11)	C6	C4	C7	110.07(11)
N1	C2	C8	142.01(12)	C7	C4	C5	111.50(11)
N1	C2	C1	69.84(9)	C9	C10	C11	119.55(12)
C8	C2	C1	148.01(12)	C3	C1	N1	115.73(10)
C10	C9	C8	119.87(11)	C2	C1	N1	49.09(8)
C9	C8	C2	119.85(11)	C2	C1	C3	117.80(11)
C9	C8	C13	120.22(11)	F1	C14	F2	106.34(11)
C13	C8	C2	119.92(11)	F1	C14	C11	112.76(11)
C12	C11	C14	118.95(11)	F3	C14	F1	106.49(10)
C10	C11	C12	121.08(12)	F3	C14	F2	106.68(11)
C10	C11	C14	119.84(11)	F3	C14	C11	113.02(11)
C13	C12	C11	119.17(12)	F2	C14	C11	111.11(10)
C12	C13	C8	120.11(12)				

Table S6. Torsion Angles for **7n**.

A	B	C	D	Angle/°	A	B	C	D	Angle/°
O1	C3	C1	N1	37.85(16)	C12	C11	C10	C9	0.29(18)
O1	C3	C1	C2	-17.56(17)	C12	C11	C14	F1	-151.30(11)
N2	C3	C1	N1	-144.27(10)	C12	C11	C14	F3	-30.47(16)
N2	C3	C1	C2	160.32(11)	C12	C11	C14	F2	89.42(14)
N1	C2	C8	C9	178.97(16)	C4	N2	C3	O1	-1.02(19)
N1	C2	C8	C13	-2.3(2)	C4	N2	C3	C1	-178.77(11)
N1	C2	C1	C3	101.11(12)	C10	C9	C8	C2	179.49(11)
C3	N2	C4	C6	176.76(11)	C10	C9	C8	C13	0.74(18)
C3	N2	C4	C5	58.46(16)	C10	C11	C12	C13	0.92(18)
C3	N2	C4	C7	-64.19(15)	C10	C11	C14	F1	32.78(16)
C2	N1	C1	C3	-105.51(12)	C10	C11	C14	F3	153.62(11)
C2	C8	C13	C12	-178.27(11)	C10	C11	C14	F2	-86.50(14)
C9	C8	C13	C12	0.48(18)	C1	N1	C2	C8	176.1(2)
C8	C2	C1	N1	-175.5(2)	C1	C2	C8	C9	-8.0(3)
C8	C2	C1	C3	-74.4(2)	C1	C2	C8	C13	170.80(17)
C8	C9	C10	C11	-1.12(18)	C14	C11	C12	C13	-174.95(11)
C11	C12	C13	C8	-1.30(18)	C14	C11	C10	C9	176.12(11)

Table S7. Hydrogen Atom Coordinates (Å×10^4) and Isotropic Displacement Parameters (Å^2×10^3) for **7n**.

**Atom**	***x***	***y***	***z***	**U(eq)**
H9	4724.07	6582.48	3527.22	24
H12	3018.08	9903.6	5262.02	23
H13	4331.14	10149.59	3658.79	23
H10	3375.53	6330.88	5062.93	25
H1	6198.4	7569.9	827.46	22
H6A	8655.8	4864.66	1516.81	38
H6B	9769.88	5177.51	2009.19	38
H6C	9252.7	5879.1	685.3	38
H5A	8308	6419.36	4847.12	43
H5B	9125.92	5429.86	4523.79	43
H5C	8000.51	5234.14	3994.61	43
H7A	9659.23	7770.3	1946.19	44
H7B	10119.42	7107.63	3342.88	44
H7C	9234.57	8038.17	3529.47	44
H2	7705(10)	6777(12)	1160(19)	15(3)

2. X-ray diffraction of 10a (CCDC 2215155)

Single crystals of 10a were grown by slow evaporation of hexane-diethyl ether solution at 4 °C. A suitable crystal was selected and studied on a SuperNova, Single source at offset/far, HyPix3000 diffractometer. The crystal was kept at 99.9(4) K during data collection. Using Olex2 [1], the structure was solved with the SHELXT [2] structure solution program using Intrinsic Phasing and refined with the SHELXL [3] refinement package using Least Squares minimisation.

Figure S2. Molecular structure of **10a** (CCDC 2215155)

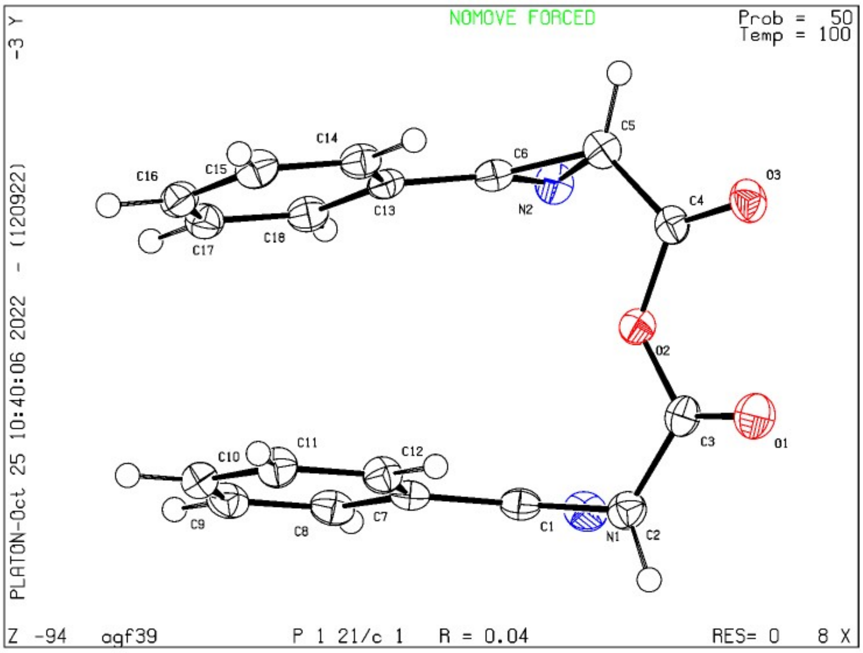

Table S8. Crystal data and structure refinement for **10a**.

Identification code	**10a**
Empirical formula	$C_{18}H_{12}N_2O_3$
Formula weight	304.30
Temperature/K	99.9(4)
Crystal system	monoclinic
Space group	$P2_1/c$
a/Å	14.8956(6)
b/Å	10.4117(4)
c/Å	9.6977(4)
α/°	90
β/°	105.934(4)
γ/°	90
Volume/Å^3	1446.22(10)
Z	4
ρ_{calc}g/cm^3	1.398
μ/mm^{-1}	0.798

F(000)	632.0
Crystal size/mm^3	0.11 × 0.1 × 0.06
Radiation	Cu Kα (λ = 1.54184)
2Θ range for data collection/°	6.17 to 124.932
Index ranges	-14 ≤ h ≤ 17, -10 ≤ k ≤ 11, -10 ≤ l ≤ 11
Reflections collected	6101
Independent reflections	2258 [R_{int} = 0.0295, R_{sigma} = 0.0356]
Data/restraints/parameters	2258/0/208
Goodness-of-fit on F^2	1.048
Final R indexes [I>=2σ (I)]	R_1 = 0.0378, wR_2 = 0.1013
Final R indexes [all data]	R_1 = 0.0452, wR_2 = 0.1053
Largest diff. peak/hole / e Å^{-3}	0.18/-0.21

Table S9. Fractional Atomic Coordinates (×10^4) and Equivalent Isotropic Displacement Parameters (Å^2×10^3) for **10a**. U_{eq} is defined as 1/3 of the trace of the orthogonalised U_{IJ} tensor.

Atom	*x*	*y*	*z*	U(eq)
O2	7405.5(6)	1596.9(10)	4675.5(10)	21.0(3)
O3	8520.8(7)	35.7(10)	5433.6(11)	27.2(3)
O1	6739.8(8)	78.4(11)	5796.7(12)	33.5(3)
N2	8418.2(8)	2730.0(12)	2970.1(12)	23.7(3)
N1	5646.6(8)	2575.7(13)	3202.4(13)	26.8(3)
C7	6145.0(9)	4497.8(14)	4911.2(16)	20.8(3)
C6	8701.0(9)	3308.8(15)	4147.2(15)	20.9(3)
C1	5915.6(9)	3220.9(15)	4333.1(16)	21.8(3)
C13	8793.4(9)	4557.4(14)	4825.1(16)	20.2(3)
C4	8282.4(9)	1060.8(14)	4899.3(14)	20.3(3)
C12	6552.0(10)	4626.2(15)	6377.2(16)	23.2(3)
C3	6684.8(10)	1052.6(14)	5129.0(15)	22.6(3)
C5	8883.0(10)	1933.5(14)	4344.5(15)	21.6(3)
C17	8565.7(10)	6837.1(15)	4671.9(17)	24.8(4)
C18	8464.1(10)	5651.6(15)	4005.4(16)	23.5(3)
C8	5953.4(10)	5580.7(15)	4027.0(16)	24.2(4)
C16	8983.8(10)	6938.0(15)	6135.4(16)	24.8(4)
C14	9221.0(10)	4659.6(15)	6288.6(16)	23.5(3)
C11	6767.3(10)	5834.1(15)	6963.5(16)	25.7(4)
C9	6167.9(10)	6780.2(15)	4624.4(17)	26.2(4)
C10	6575.4(10)	6915.3(15)	6090.9(17)	25.8(4)
C15	9314.7(10)	5852.2(16)	6944.7(16)	25.7(3)
C2	5849.2(10)	1871.6(15)	4676.8(16)	24.4(4)

Table S10. Anisotropic Displacement Parameters (Å^2×10^3) for **10a**. The Anisotropic displacement factor exponent takes the form: $-2\pi^2[h^2a^{*2}U_{11}+2hka^*b^*U_{12}+\ldots]$.

**Atom**	**U_{11}**	**U_{22}**	**U_{33}**	**U_{23}**	**U_{13}**	**U_{12}**
O2	20.2(5)	21.8(6)	19.0(5)	2.0(4)	2.1(4)	-0.2(4)
O3	29.8(6)	21.0(6)	28.1(6)	3.9(4)	3.5(4)	3.3(4)
O1	36.2(6)	25.9(7)	40.4(7)	8.8(5)	13.8(5)	-0.2(5)
N2	27.3(6)	24.1(7)	18.5(7)	3.1(5)	4.0(5)	-0.1(5)
N1	23.5(6)	31.9(8)	20.9(7)	-0.7(6)	-0.7(5)	3.6(5)
C7	14.9(7)	26.6(8)	20.8(7)	1.3(6)	4.8(5)	2.4(6)
C6	16.4(7)	26.7(8)	19.4(8)	2.2(6)	4.3(5)	-1.0(6)
C1	16.1(7)	28.4(8)	19.6(8)	0.7(6)	3.0(5)	2.0(6)
C13	15.0(7)	23.1(8)	22.7(8)	1.9(6)	5.7(5)	-2.0(6)
C4	22.4(7)	20.7(8)	14.8(7)	-3.3(6)	-0.1(5)	0.4(6)
C12	21.7(7)	25.4(8)	21.1(8)	3.0(6)	3.4(6)	1.4(6)
C3	26.4(7)	22.1(8)	18.2(7)	-2.5(6)	4.3(6)	-3.2(6)
C5	23.3(7)	24.3(8)	15.5(7)	0.3(6)	2.4(5)	1.4(6)
C17	20.7(7)	24.2(8)	29.3(9)	3.7(6)	6.4(6)	1.3(6)
C18	19.5(7)	28.9(9)	20.2(8)	1.3(6)	2.5(6)	-0.2(6)
C8	21.0(7)	31.6(9)	19.8(8)	4.9(6)	5.0(6)	4.9(6)
C16	21.9(7)	26.3(9)	27.2(8)	-5.3(6)	8.3(6)	-3.7(6)
C14	20.5(7)	28.2(8)	21.9(8)	3.2(6)	5.9(6)	-0.1(6)
C11	24.8(7)	27.8(8)	21.0(8)	-0.8(6)	0.7(6)	-0.4(6)
C9	23.2(7)	27.3(9)	29.8(9)	9.5(6)	10.2(6)	5.3(6)
C10	22.2(7)	23.3(8)	31.6(9)	-0.5(6)	7.0(6)	-0.9(6)
C15	23.9(7)	32.8(9)	19.2(8)	-1.3(6)	3.7(6)	-3.0(6)
C2	23.7(7)	26.4(8)	22.8(8)	-2.3(6)	5.7(6)	-2.0(6)

Table S11. Bond Lengths for **10a**.

**Atom**	**Atom**	**Length/Å**	**Atom**	**Atom**	**Length/Å**
O2	C4	1.3817(17)	C1	C2	1.454(2)
O2	C3	1.3880(17)	C13	C18	1.399(2)
O3	C4	1.1970(18)	C13	C14	1.391(2)
O1	C3	1.1940(19)	C4	C5	1.476(2)
N2	C6	1.257(2)	C12	C11	1.381(2)
N2	C5	1.5611(18)	C3	C2	1.473(2)
N1	C1	1.254(2)	C17	C18	1.382(2)
N1	C2	1.5608(19)	C17	C16	1.388(2)
C7	C1	1.447(2)	C8	C9	1.377(2)
C7	C12	1.391(2)	C16	C15	1.387(2)
C7	C8	1.398(2)	C14	C15	1.385(2)
C6	C13	1.446(2)	C11	C10	1.390(2)
C6	C5	1.460(2)	C9	C10	1.392(2)

Table S12. Bond Angles for **10a**.

Atom	Atom	Atom	Angle/°	Atom	Atom	Atom	Angle/°
C4	O2	C3	124.43(11)	O2	C3	C2	109.47(12)
C6	N2	C5	61.30(10)	O1	C3	O2	125.08(13)
C1	N1	C2	61.03(10)	O1	C3	C2	125.45(14)
C12	C7	C1	118.35(13)	C6	C5	N2	49.03(9)
C12	C7	C8	120.53(14)	C6	C5	C4	122.82(13)
C8	C7	C1	121.12(13)	C4	C5	N2	117.84(12)
N2	C6	C13	144.12(14)	C18	C17	C16	120.42(14)
N2	C6	C5	69.68(11)	C17	C18	C13	119.04(13)
C13	C6	C5	146.19(13)	C9	C8	C7	119.27(13)
N1	C1	C7	144.66(14)	C15	C16	C17	120.44(14)
N1	C1	C2	69.96(12)	C15	C14	C13	119.84(14)
C7	C1	C2	145.39(14)	C12	C11	C10	120.07(14)
C18	C13	C6	119.94(13)	C8	C9	C10	120.49(14)
C14	C13	C6	119.50(13)	C11	C10	C9	119.96(14)
C14	C13	C18	120.56(14)	C14	C15	C16	119.70(13)
O2	C4	C5	109.60(12)	C1	C2	N1	49.02(9)
O3	C4	O2	125.70(13)	C1	C2	C3	121.52(13)
O3	C4	C5	124.69(13)	C3	C2	N1	118.67(12)
C11	C12	C7	119.69(14)				

Table S13. Torsion Angles for **10a**.

A	B	C	D	Angle/°	A	B	C	D	Angle/°
O2	C4	C5	N2	-34.82(16)	C13	C6	C5	C4	81.2(3)
O2	C4	C5	C6	22.38(18)	C13	C14	C15	C16	-0.2(2)
O2	C3	C2	N1	35.90(17)	C4	O2	C3	O1	2.1(2)
O2	C3	C2	C1	-21.31(19)	C4	O2	C3	C2	-178.71(11)
O3	C4	C5	N2	143.82(14)	C12	C7	C1	N1	-171.19(19)
O3	C4	C5	C6	-158.99(14)	C12	C7	C1	C2	8.6(3)
O1	C3	C2	N1	-144.90(15)	C12	C7	C8	C9	-0.3(2)
O1	C3	C2	C1	157.89(15)	C12	C11	C10	C9	-0.2(2)
N2	C6	C13	C18	1.2(3)	C3	O2	C4	O3	4.5(2)
N2	C6	C13	C14	-178.02(18)	C3	O2	C4	C5	-176.93(11)
N2	C6	C5	C4	-100.13(15)	C5	N2	C6	C13	178.7(2)
N1	C1	C2	C3	102.28(15)	C5	C6	C13	C18	178.99(19)
C7	C1	C2	N1	-179.9(2)	C5	C6	C13	C14	-0.2(3)
C7	C1	C2	C3	-77.6(3)	C17	C16	C15	C14	-0.4(2)
C7	C12	C11	C10	0.2(2)	C18	C13	C14	C15	0.6(2)
C7	C8	C9	C10	0.3(2)	C18	C17	C16	C15	0.8(2)
C6	N2	C5	C4	110.68(15)	C8	C7	C1	N1	9.6(3)

Table S13. Torsion Angles for **10a**.

A	B	C	D	Angle/°	A	B	C	D	Angle/°
C6	C13	C18	C17	-179.49(12)	C8	C7	C1	C2	-170.58(19)
C6	C13	C14	C15	179.80(12)	C8	C7	C12	C11	0.1(2)
C1	N1	C2	C3	-108.31(15)	C8	C9	C10	C11	-0.1(2)
C1	C7	C12	C11	-179.12(13)	C16	C17	C18	C13	-0.4(2)
C1	C7	C8	C9	178.86(13)	C14	C13	C18	C17	-0.3(2)
C13	C6	C5	N2	-178.6(2)	C2	N1	C1	C7	179.9(2)

Table S14. Hydrogen Atom Coordinates (Å×10^4) and Isotropic Displacement Parameters (Å^2×10^3) for **10a**.

Atom	*x*	*y*	*z*	U(eq)
H12	6681.44	3886.59	6972.72	28
H5	9540.98	1647.99	4463.78	26
H17	8348.15	7587.71	4125.19	30
H18	8174.77	5581.03	3005.21	28
H8	5678.24	5490.15	3024.99	29
H16	9043.65	7756.06	6585.53	30
H14	9448.29	3913.03	6836.67	28
H11	7046.85	5926.05	7964.26	31
H9	6036.78	7521.06	4031.08	31
H10	6722.32	7745.92	6495.32	31
H15	9604.27	5926.54	7944.55	31
H2	5295.46	1609.49	5004.88	29

3. References

[1] Dolomanov, O. V.; Bourhis, L. J.; Gildea, R. J.; Howard, J. A. K.; Puschmann, H. *J. Appl. Cryst.* **2009**, 42, 339–341.

[2] Sheldrick, G.M. *Acta Cryst.* **2015**, A71, 3–8.

[3] Sheldrick, G.M. *Acta Cryst.* **2015**, C71, 3–8.

4. NMR spectra of new compounds

^{1}H NMR spectra ($CDCl_3$, 400 MHz) of compound **2c**

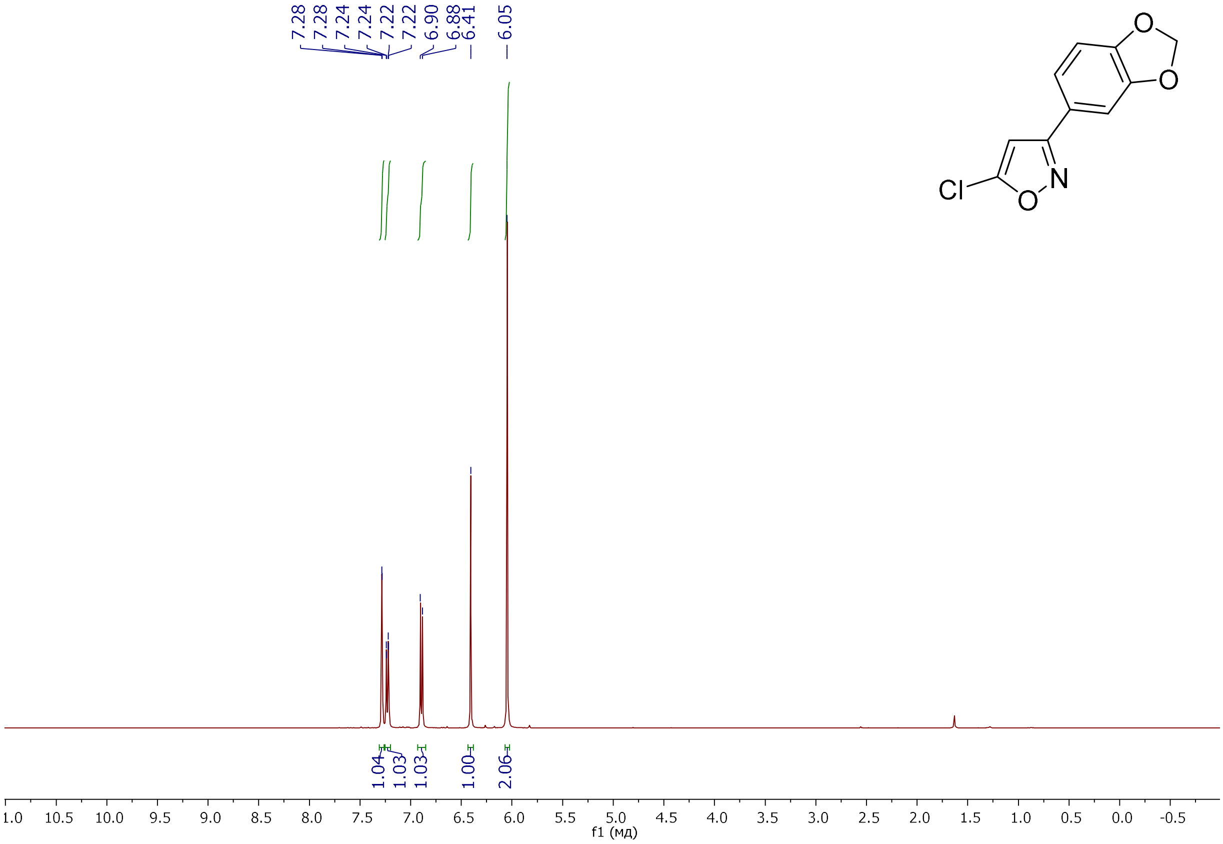

$^{13}C\{^{1}H\}$ NMR spectra ($CDCl_3$, 100 MHz) of compound **2c**

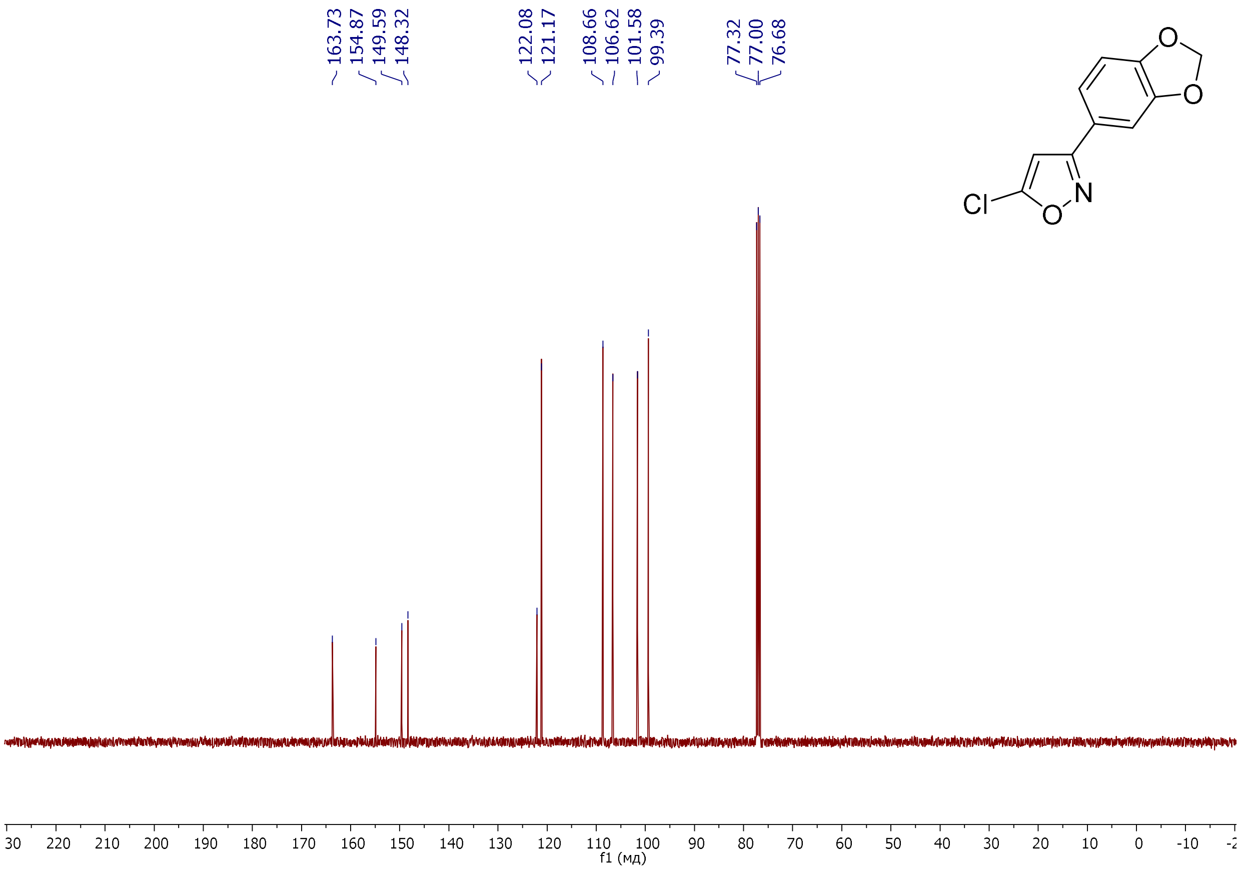

^{1}H NMR spectra ($CDCl_3$, 400 MHz) of compound **2d**

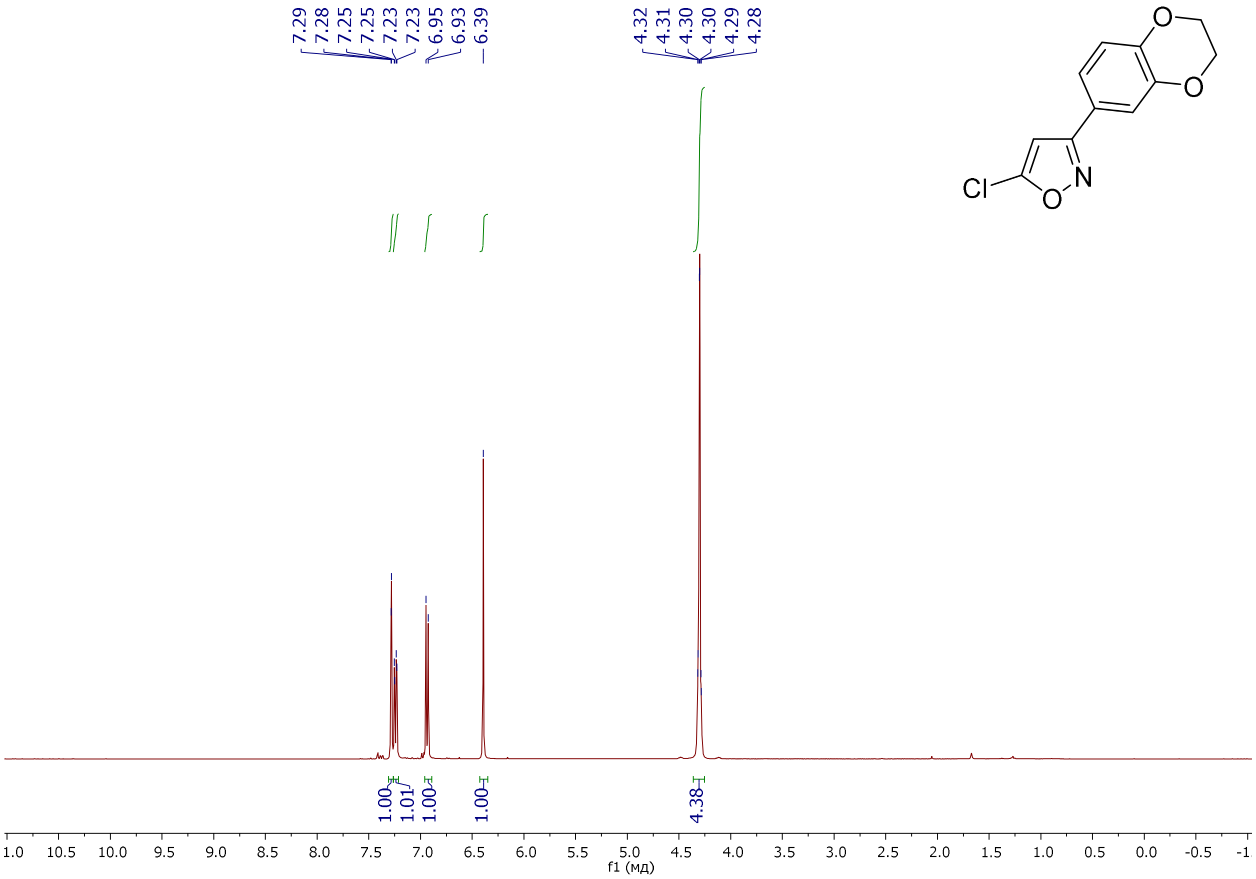

^{13}C{^{1}H} NMR spectra ($CDCl_3$, 100 MHz) of compound **2d**

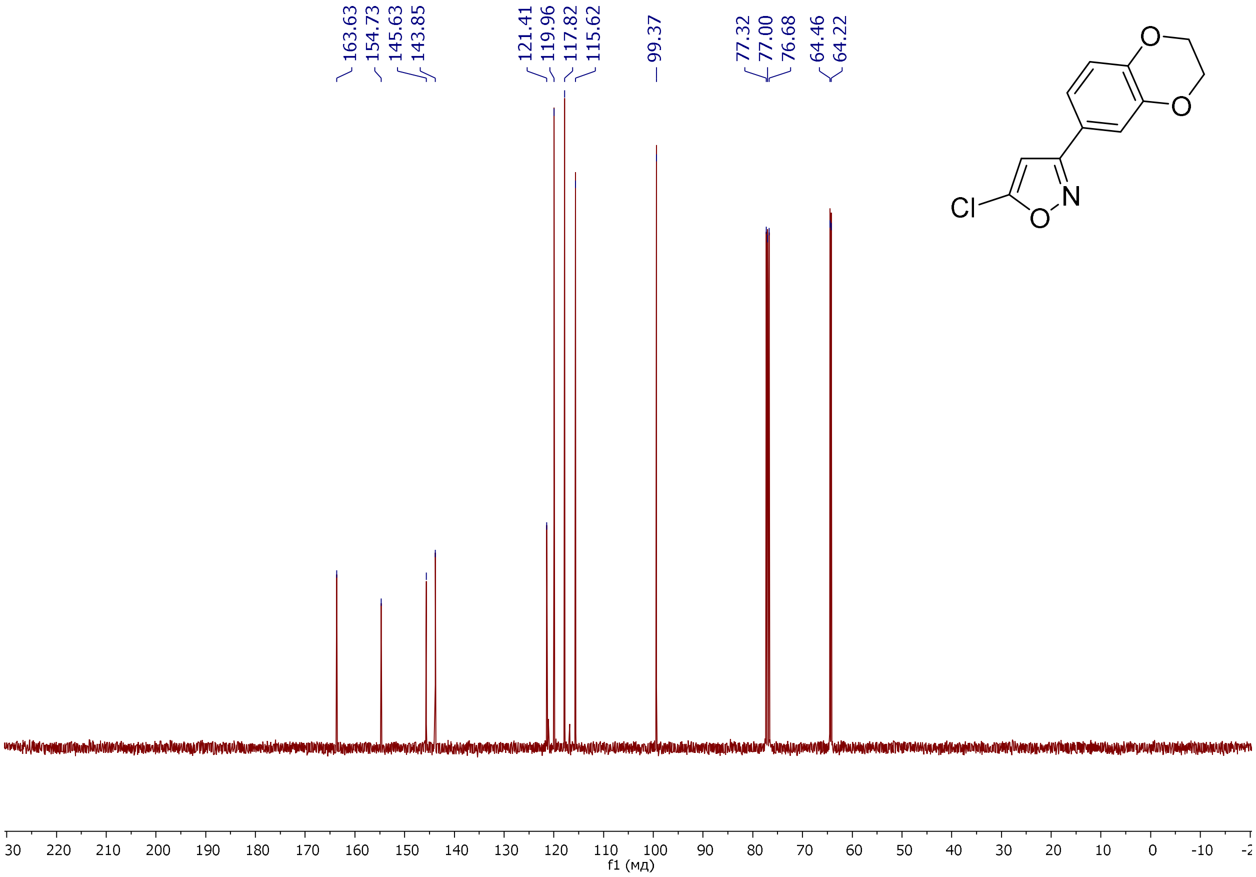

^{1}H NMR spectra ($CDCl_3$, 400 MHz) of compound **2e**

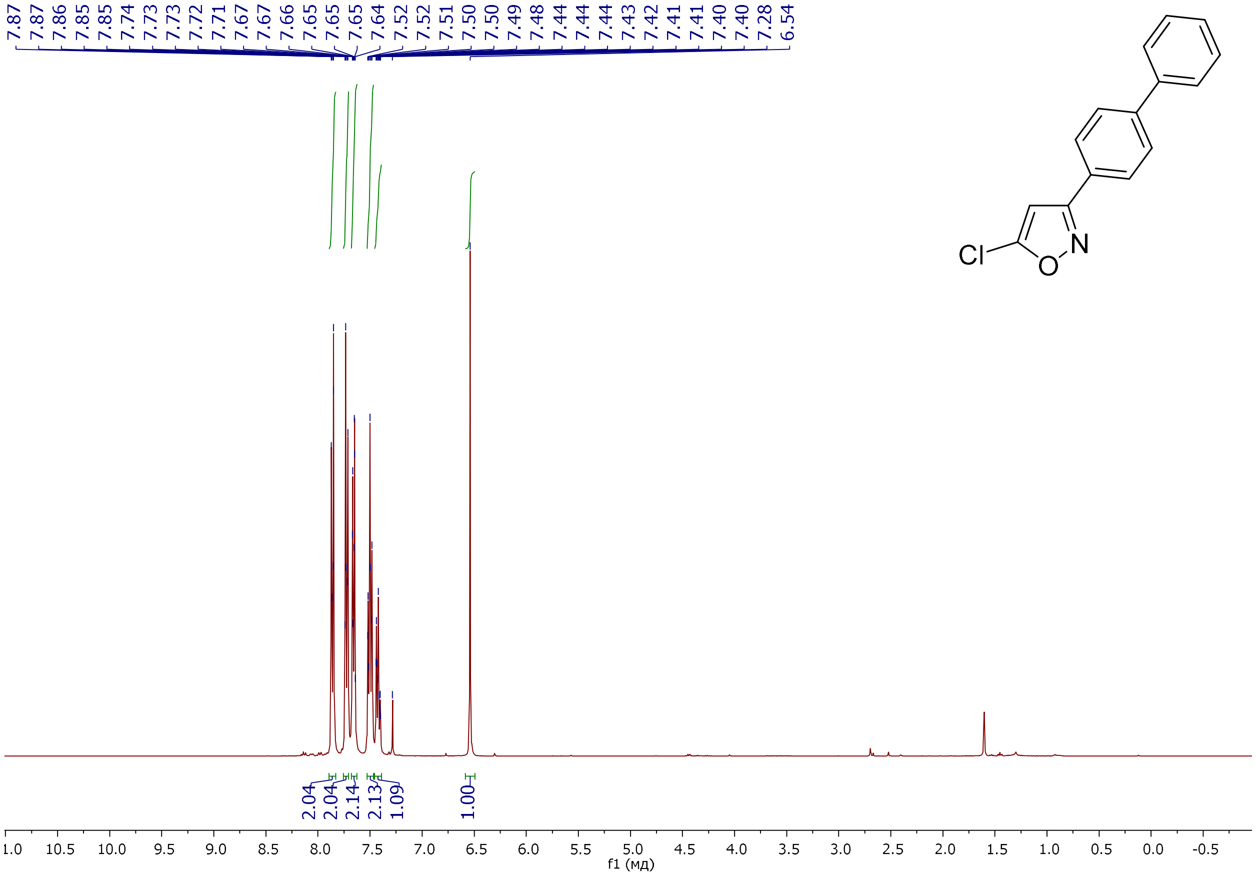

^{13}C{^{1}H} NMR spectra ($CDCl_3$, 100 MHz) of compound **2e**

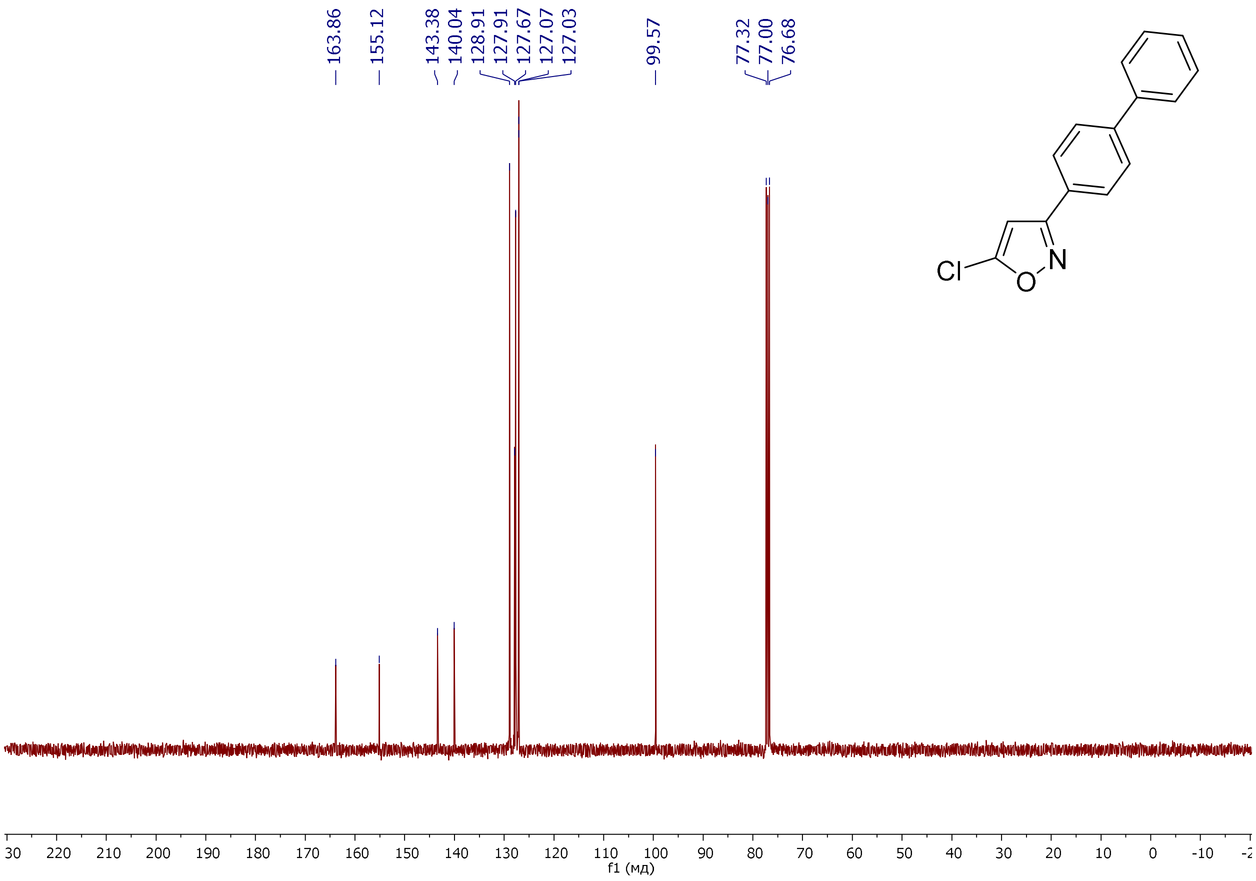

^{1}H NMR spectra ($CDCl_3$, 400 MHz) of compound **7a**

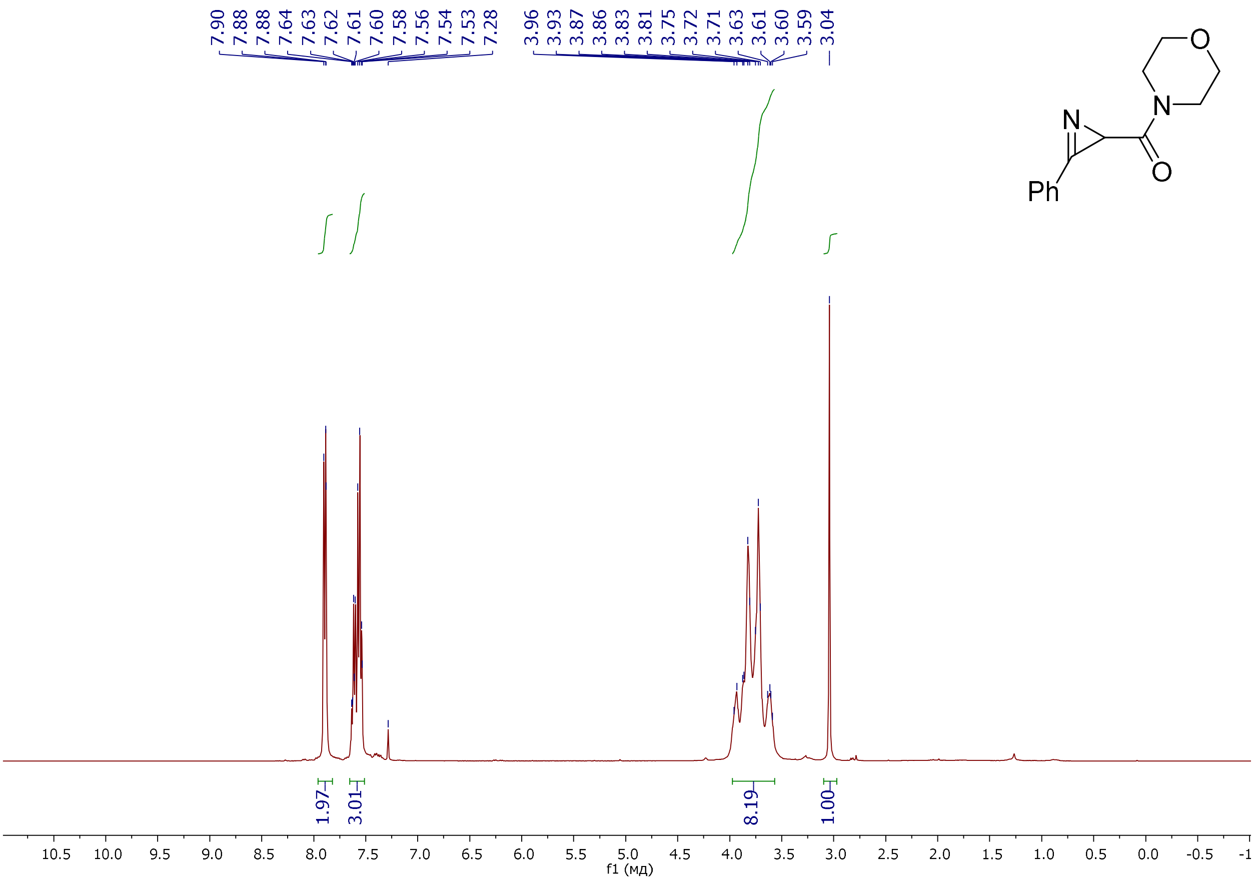

^{13}C{^{1}H} NMR spectra ($CDCl_3$, 100 MHz) of compound **7a**

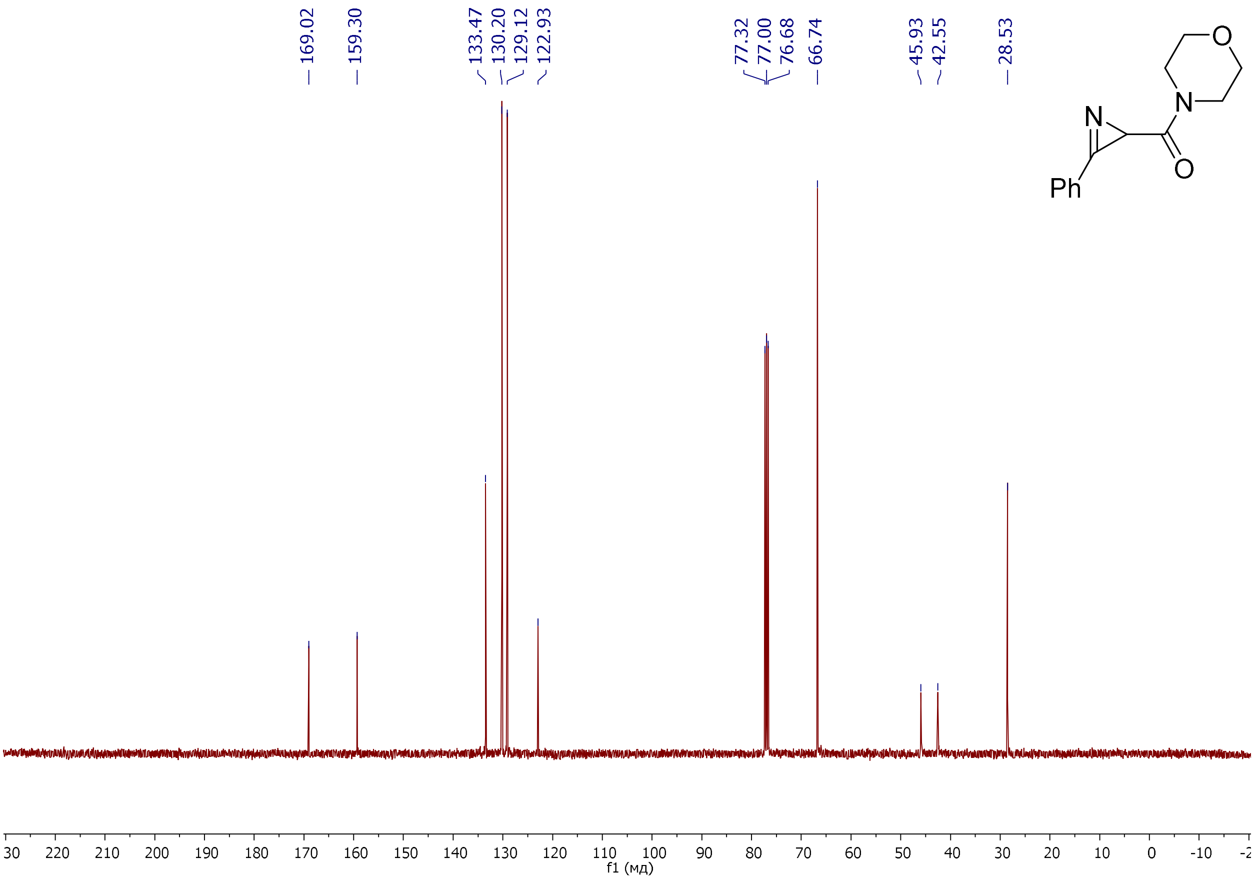

^{1}H NMR spectra ($CDCl_3$, 400 MHz) of compound **7b**

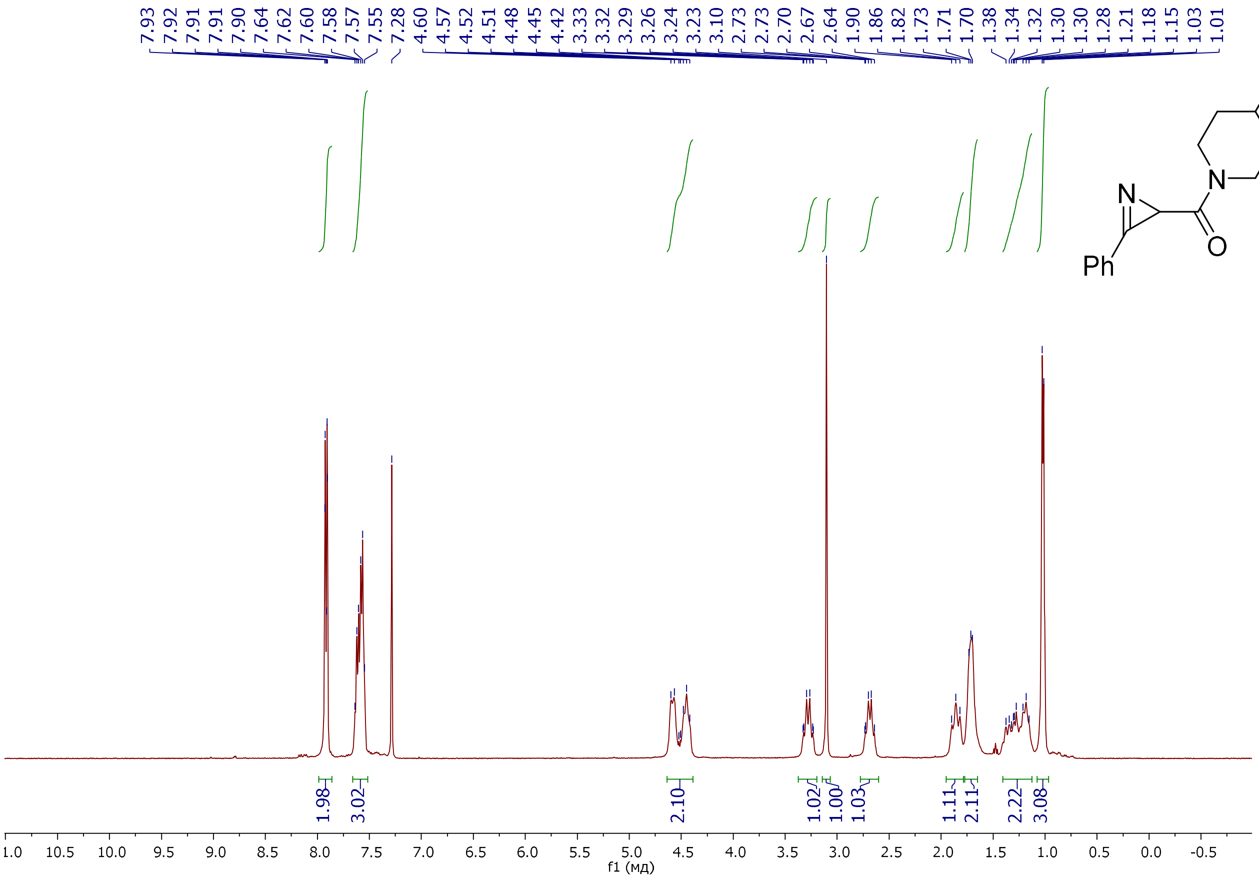

^{13}C{^{1}H} NMR spectra ($CDCl_3$, 100 MHz) of compound **7b**

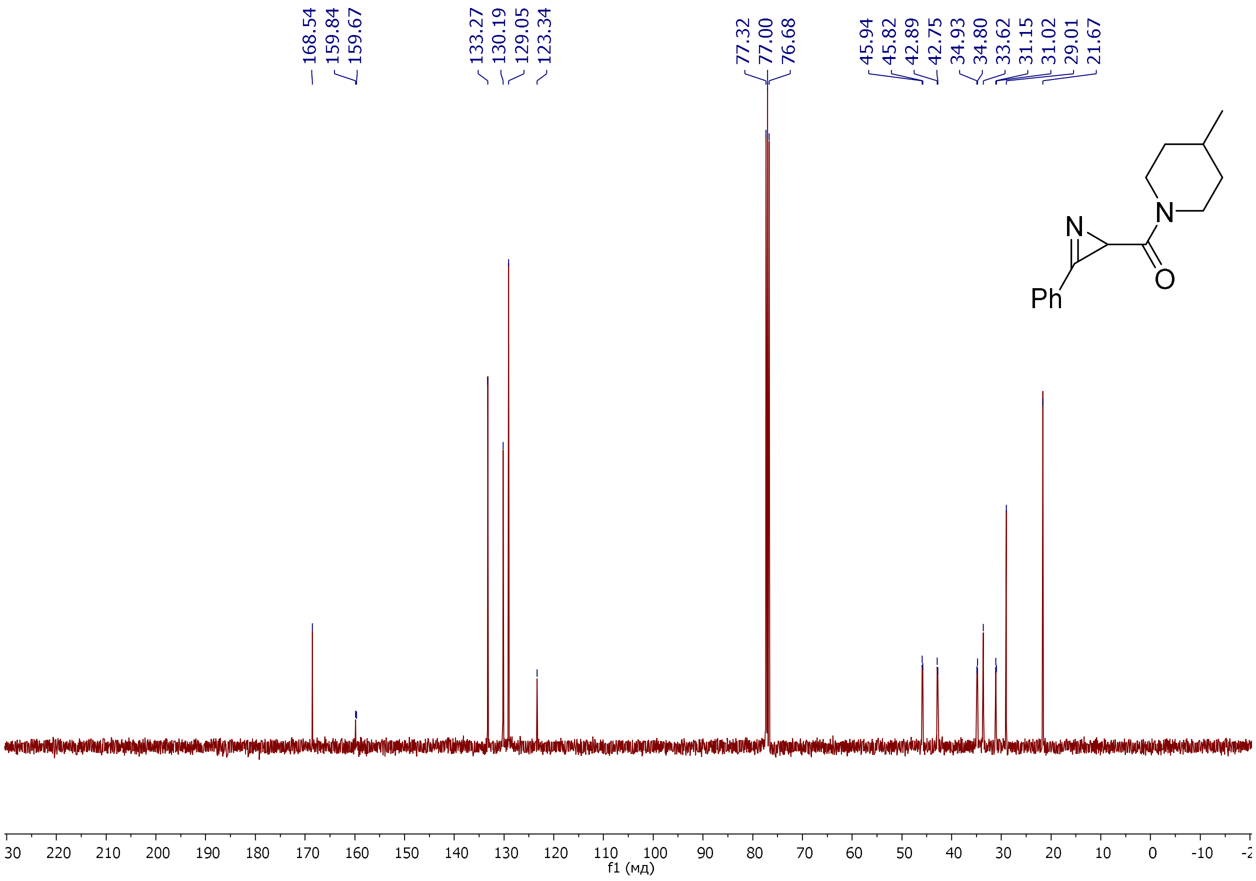

^{1}H NMR spectra ($CDCl_3$, 400 MHz) of compound **7c**

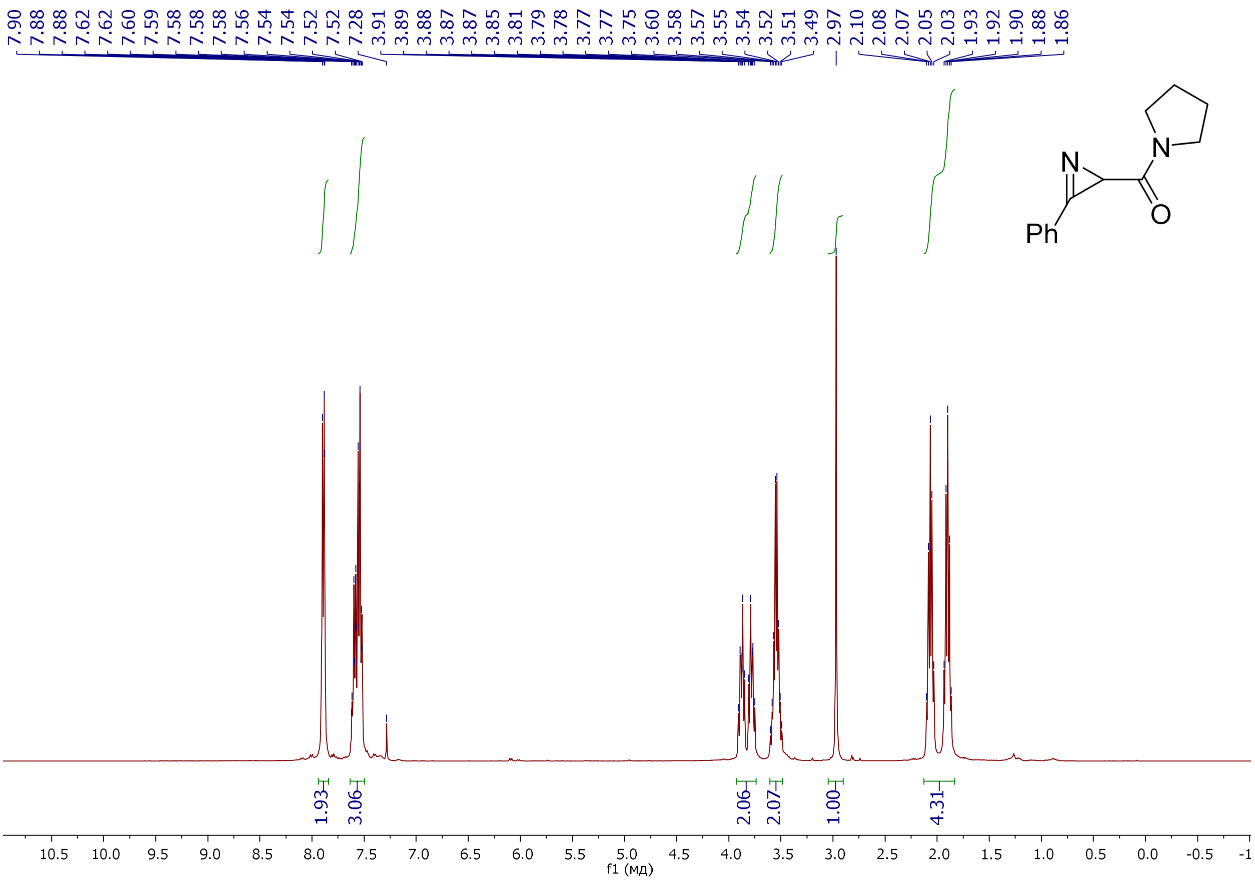

$^{13}C\{^1H\}$ NMR spectra ($CDCl_3$, 100 MHz) of compound **7c**

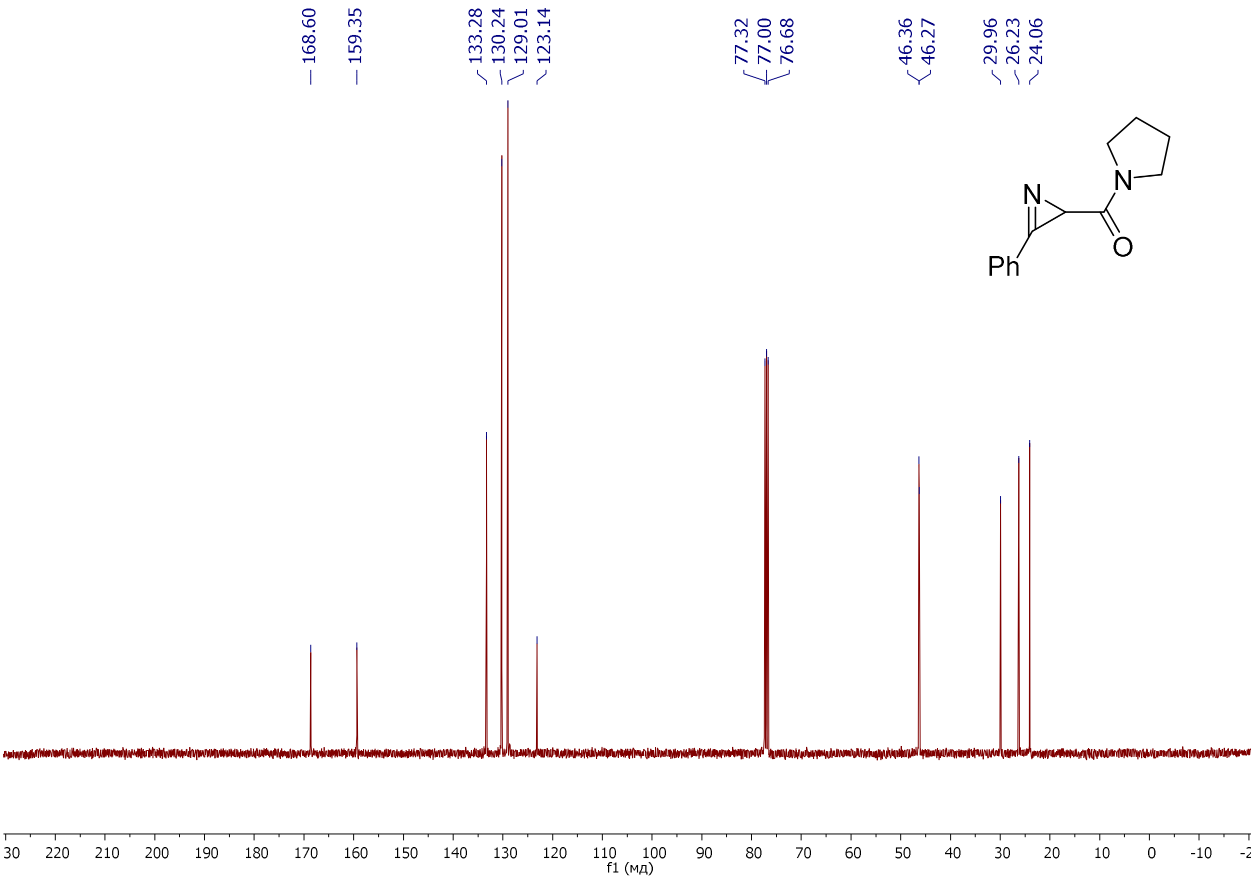

^{1}H NMR spectra ($CDCl_3$, 400 MHz) of compound **7d**

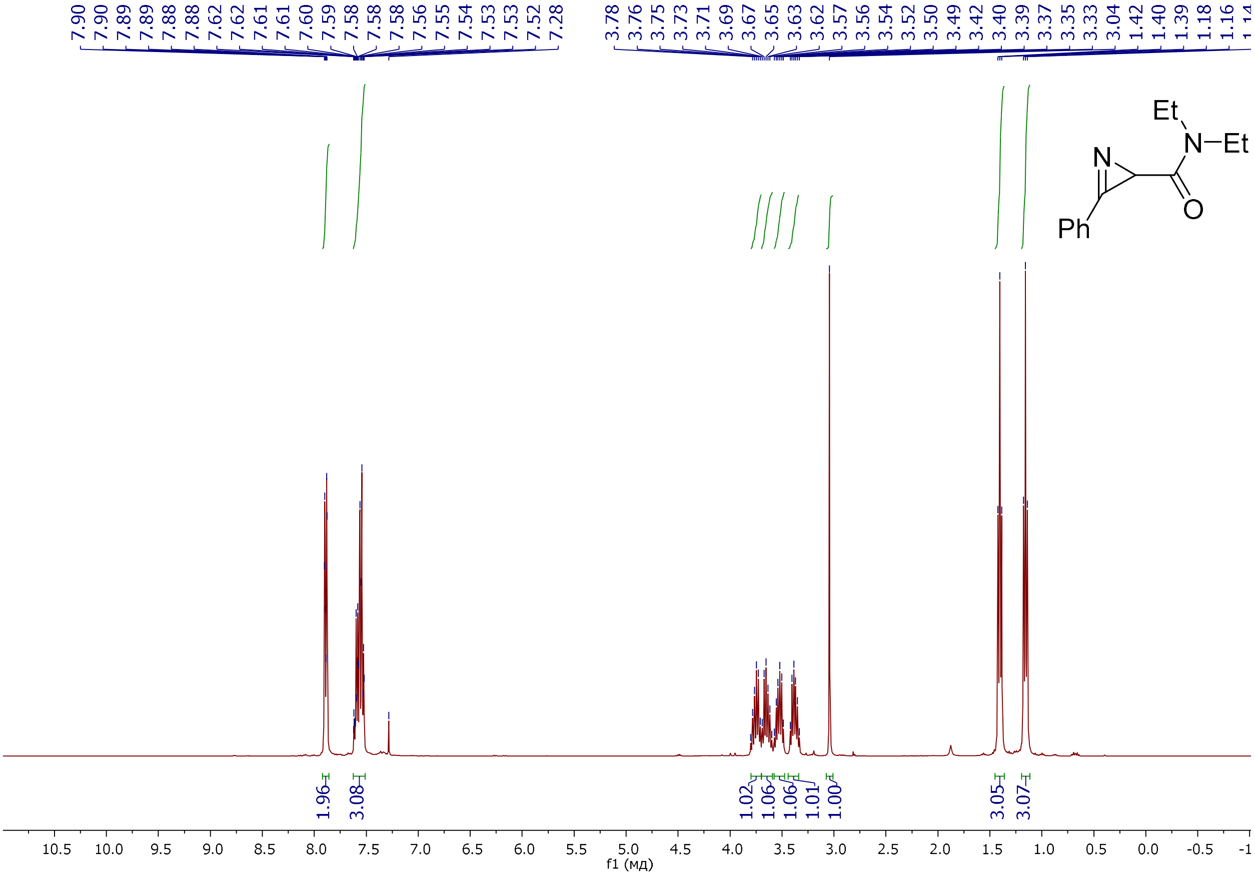

^{13}C{^{1}H} NMR spectra ($CDCl_3$, 100 MHz) of compound **7d**

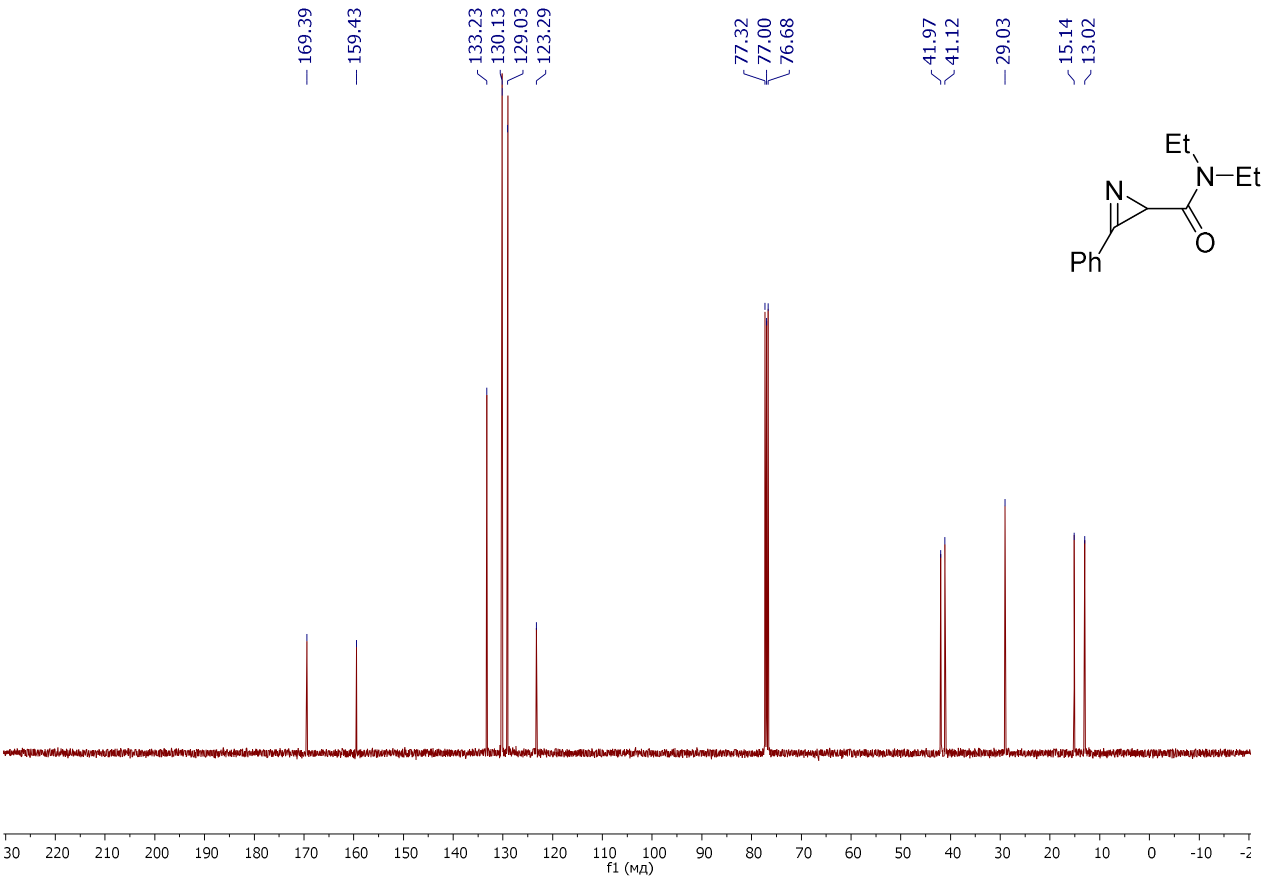

^{1}H NMR spectra ($CDCl_3$, 400 MHz) of compound **7e**

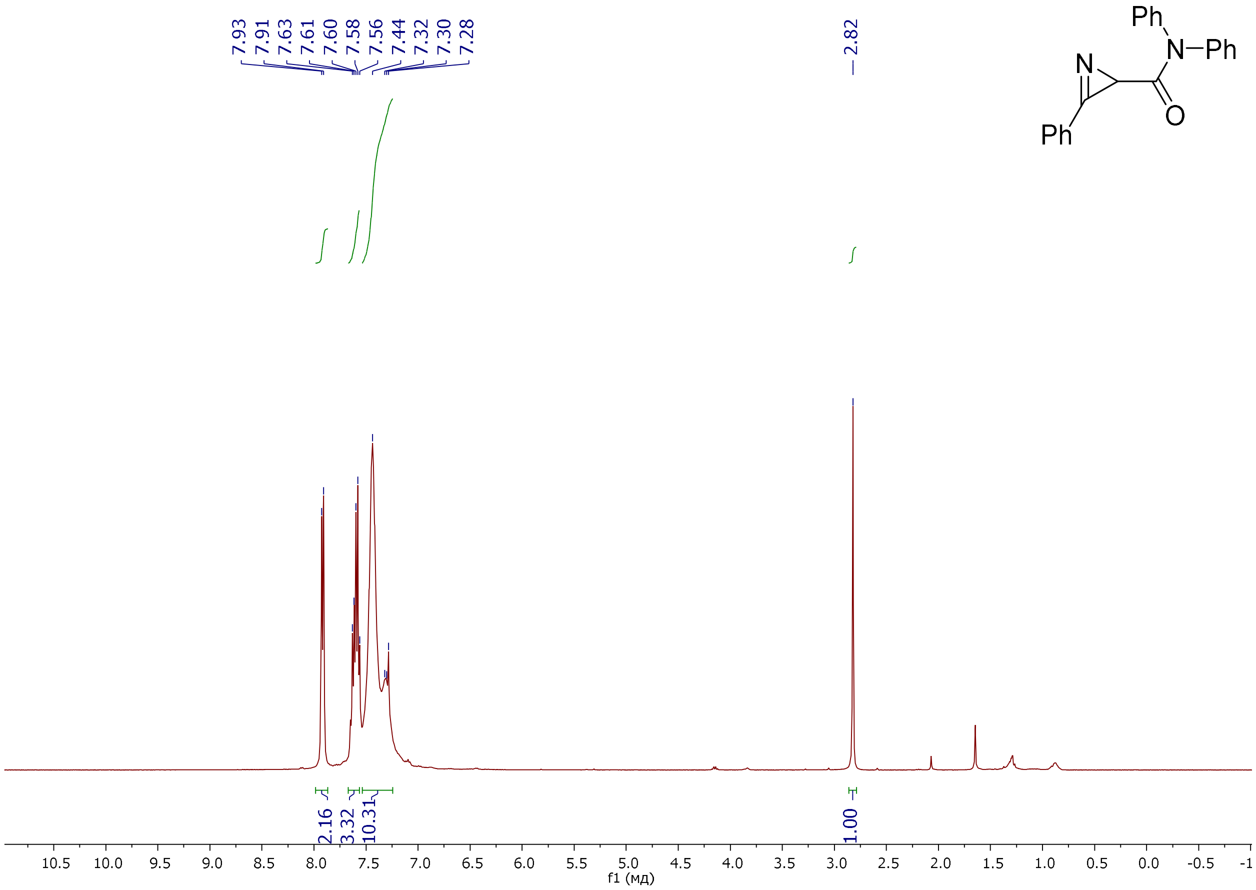

$^{13}C\{^{1}H\}$ NMR spectra ($CDCl_3$, 100 MHz) of compound **7e**

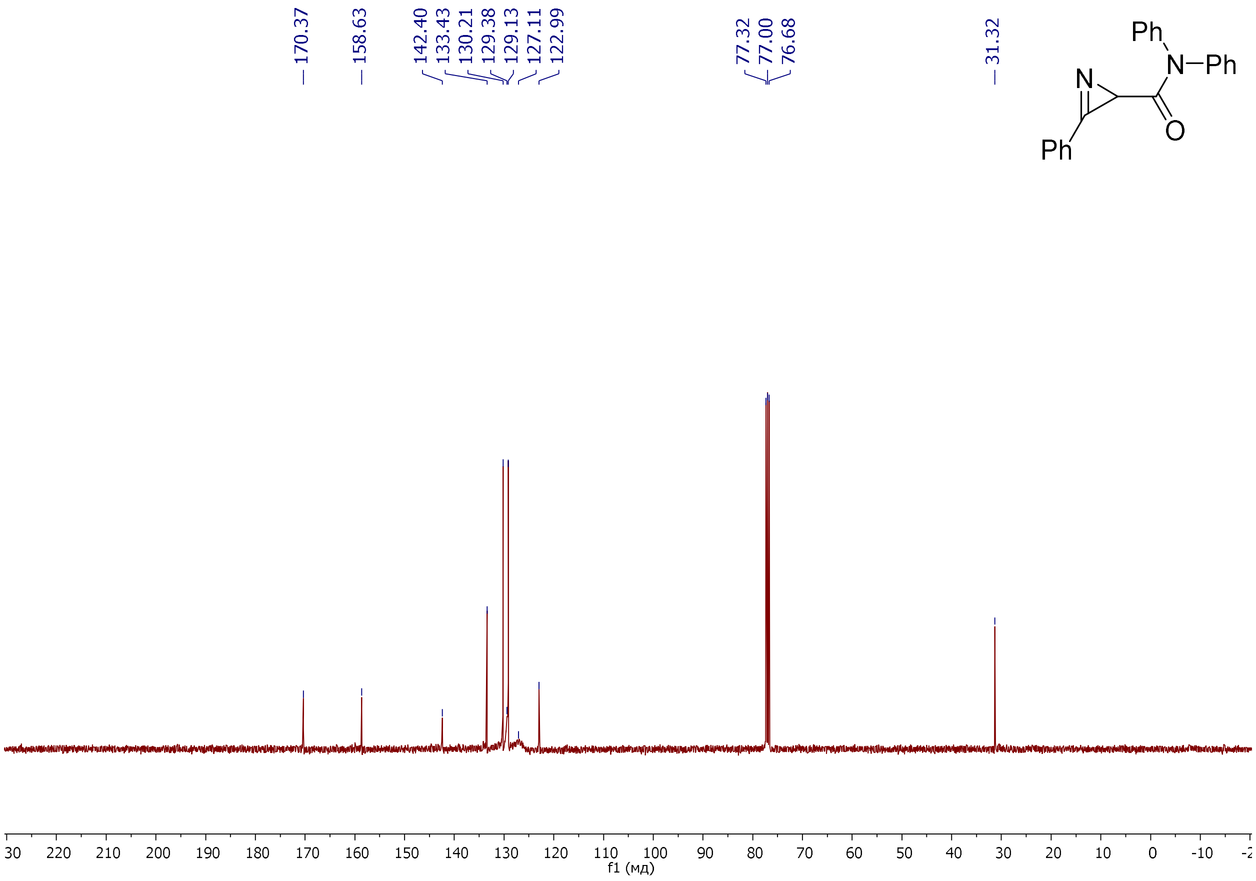

^{1}H NMR spectra ($CDCl_3$, 400 MHz) of compound **7f**

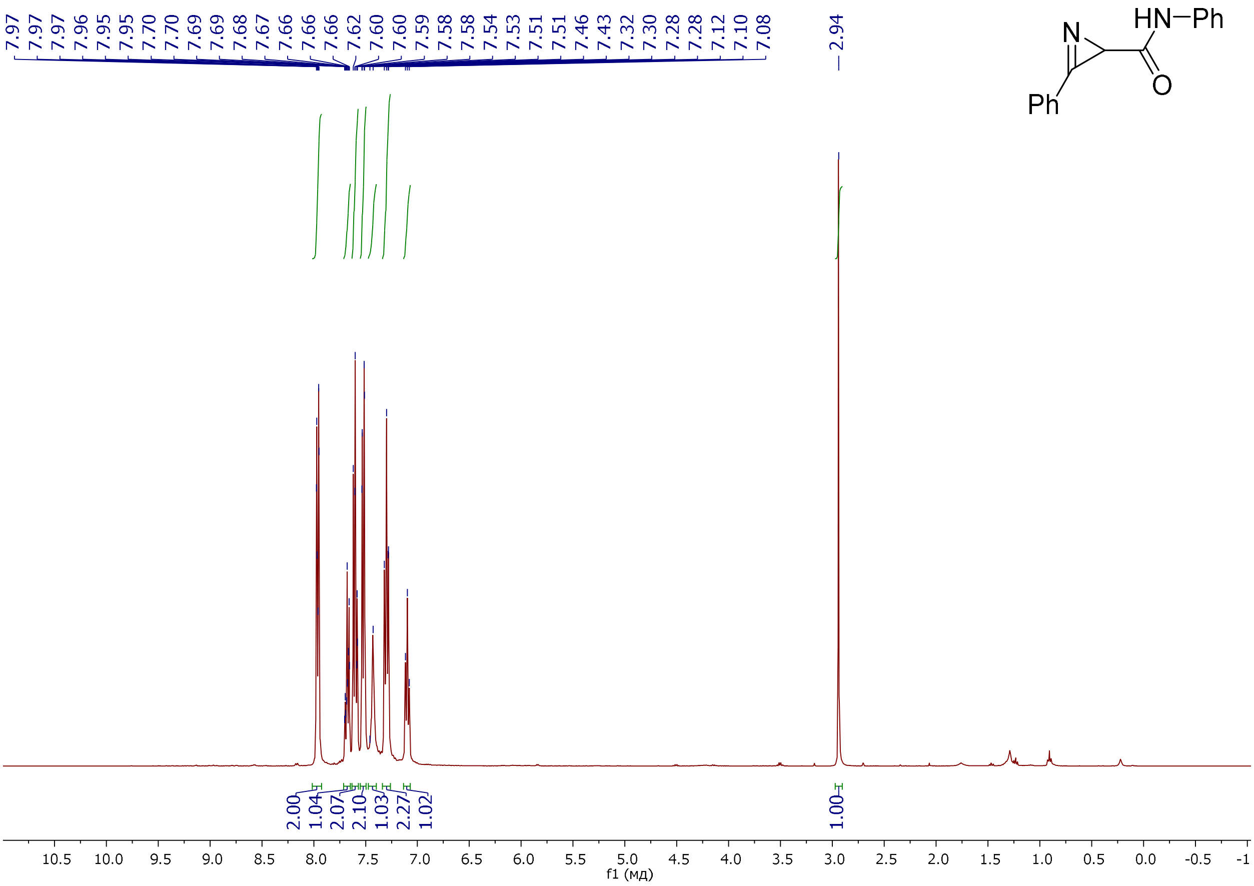

^{13}C{^{1}H} NMR spectra ($CDCl_3$, 100 MHz) of compound **7f**

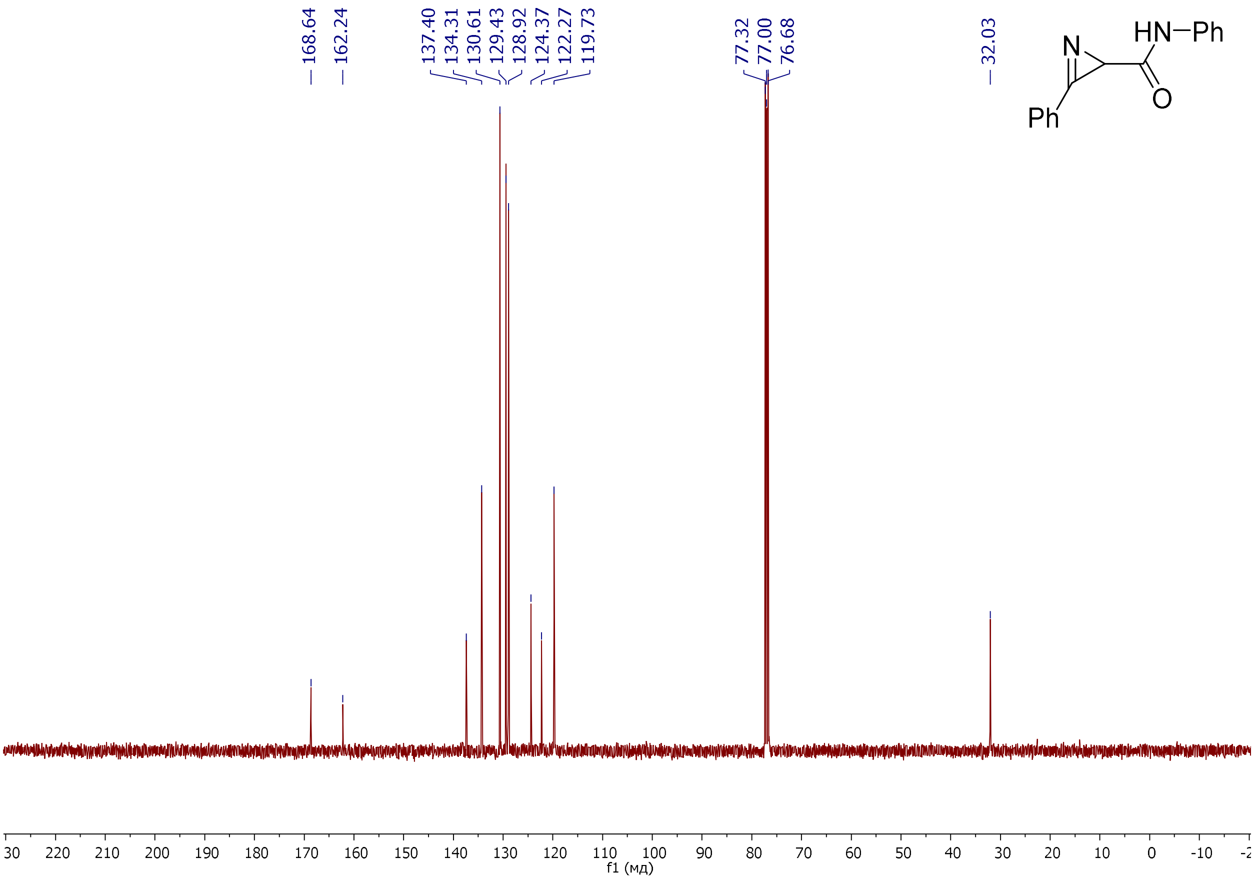

^{1}H NMR spectra ($CDCl_3$, 400 MHz) of compound **7g**

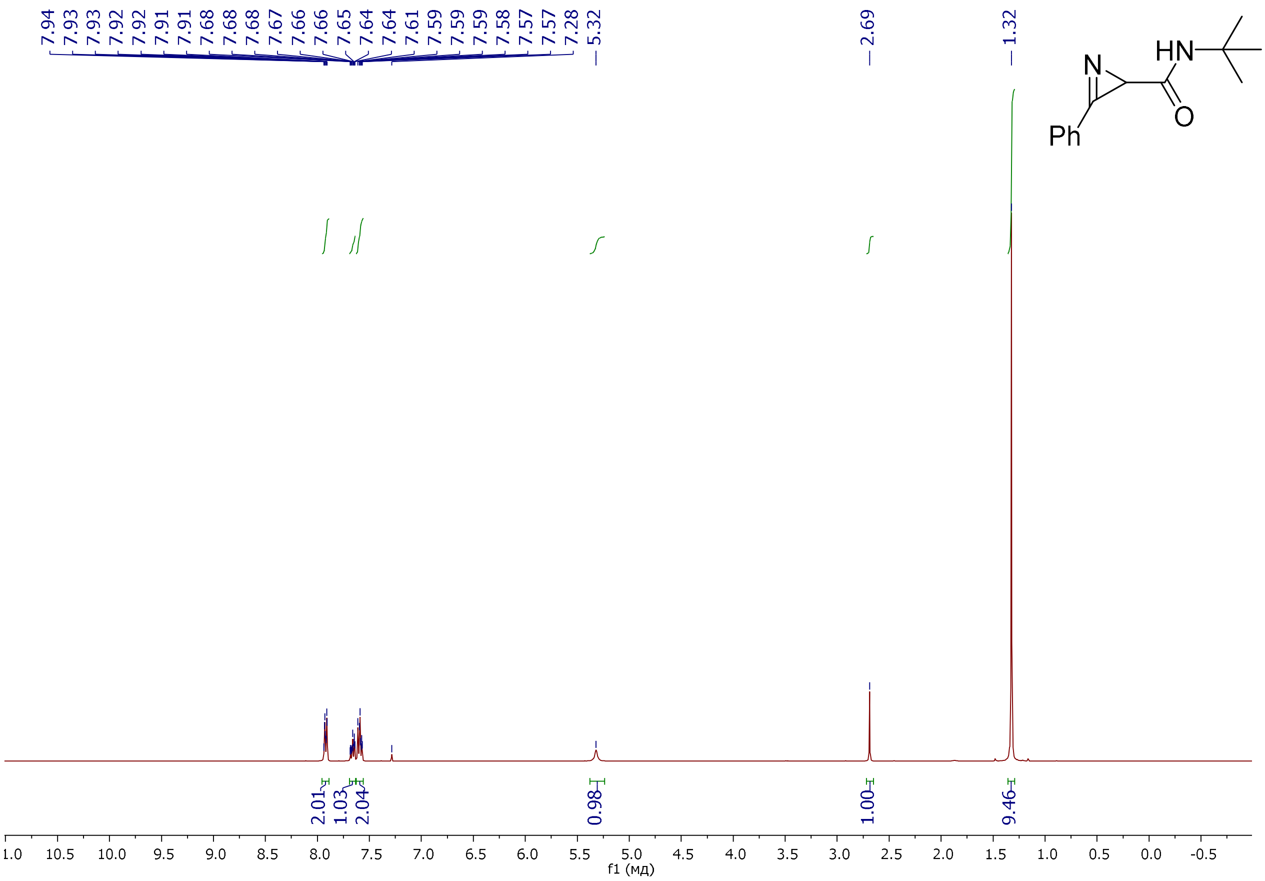

^{13}C{^{1}H} NMR spectra ($CDCl_3$, 100 MHz) of compound **7g**

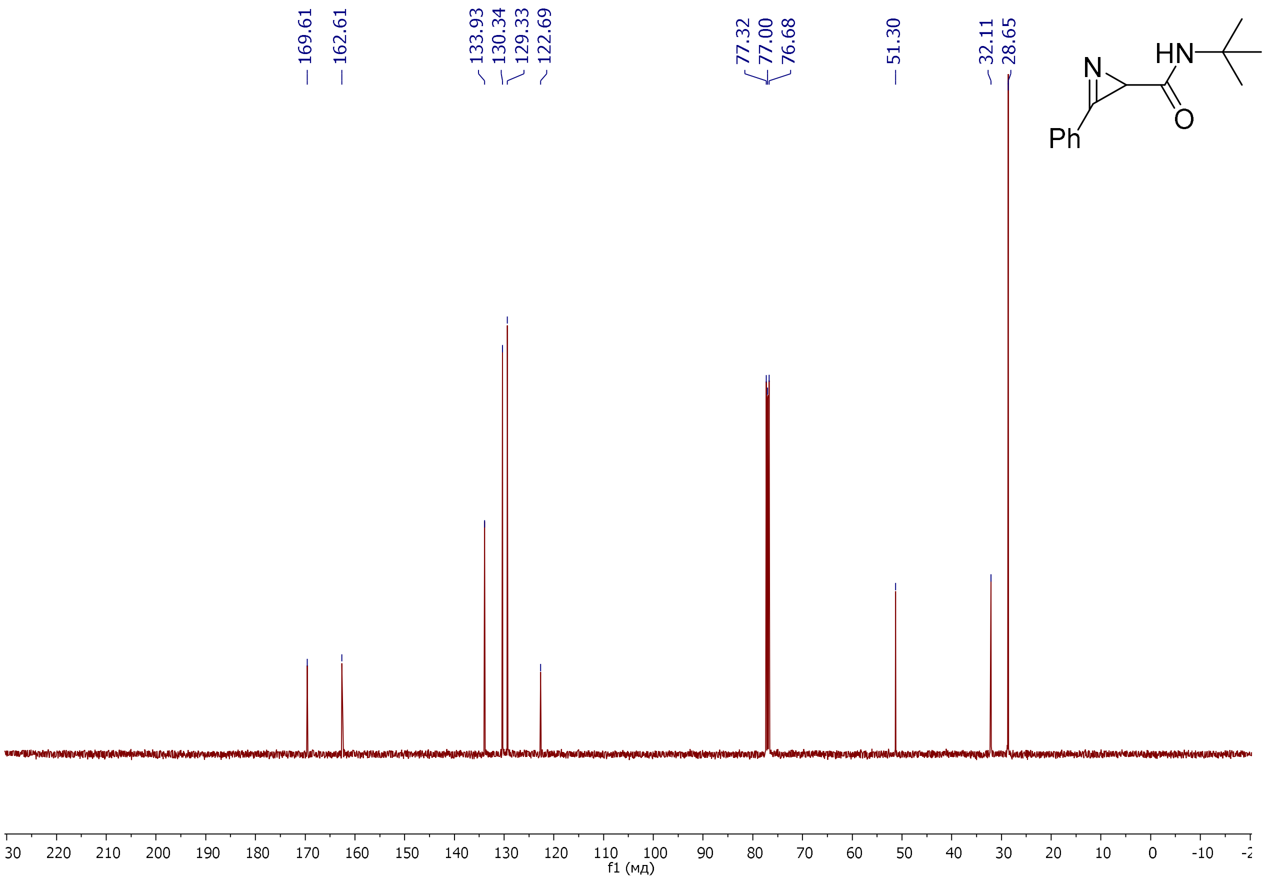

^{1}H NMR spectra ($CDCl_3$, 400 MHz) of compound **7h**

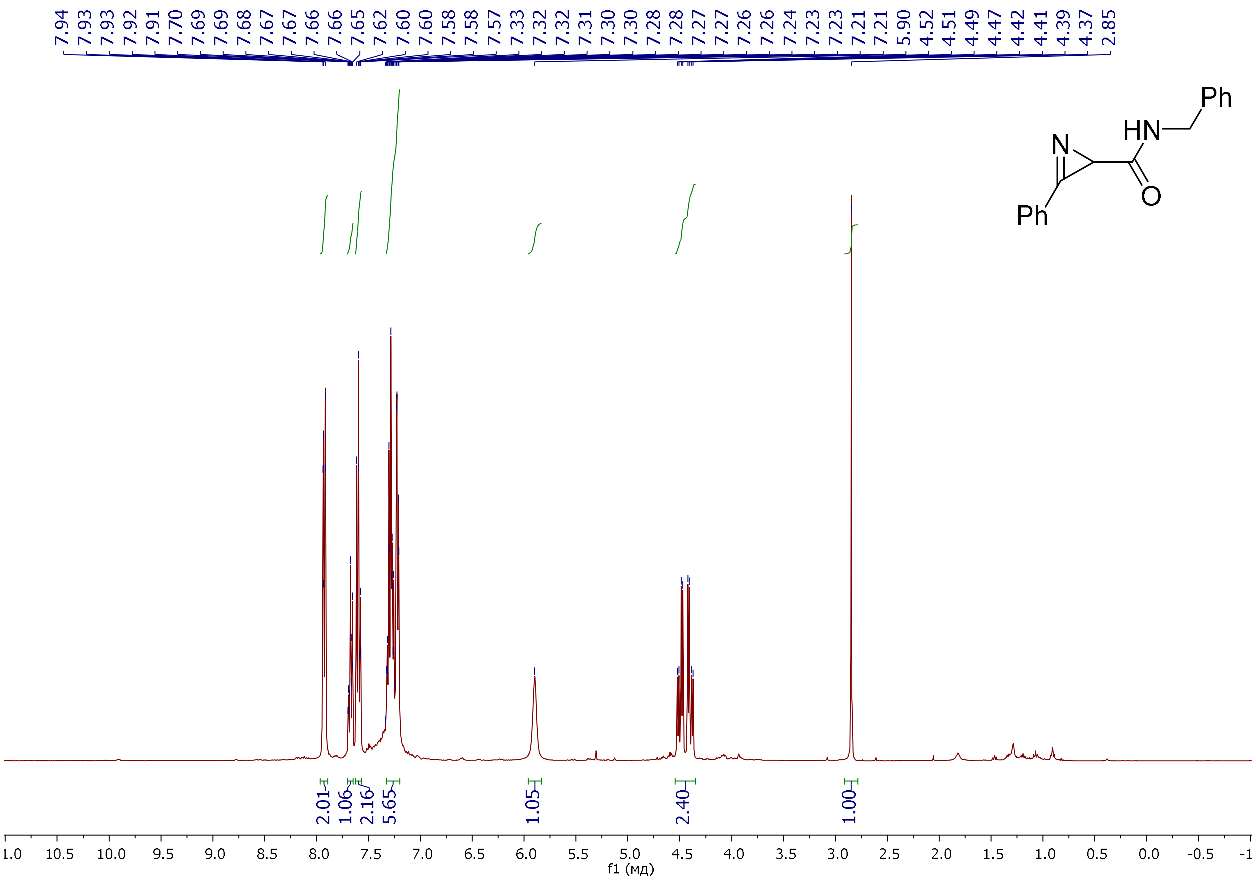

^{13}C{^{1}H} NMR spectra ($CDCl_3$, 100 MHz) of compound **7h**

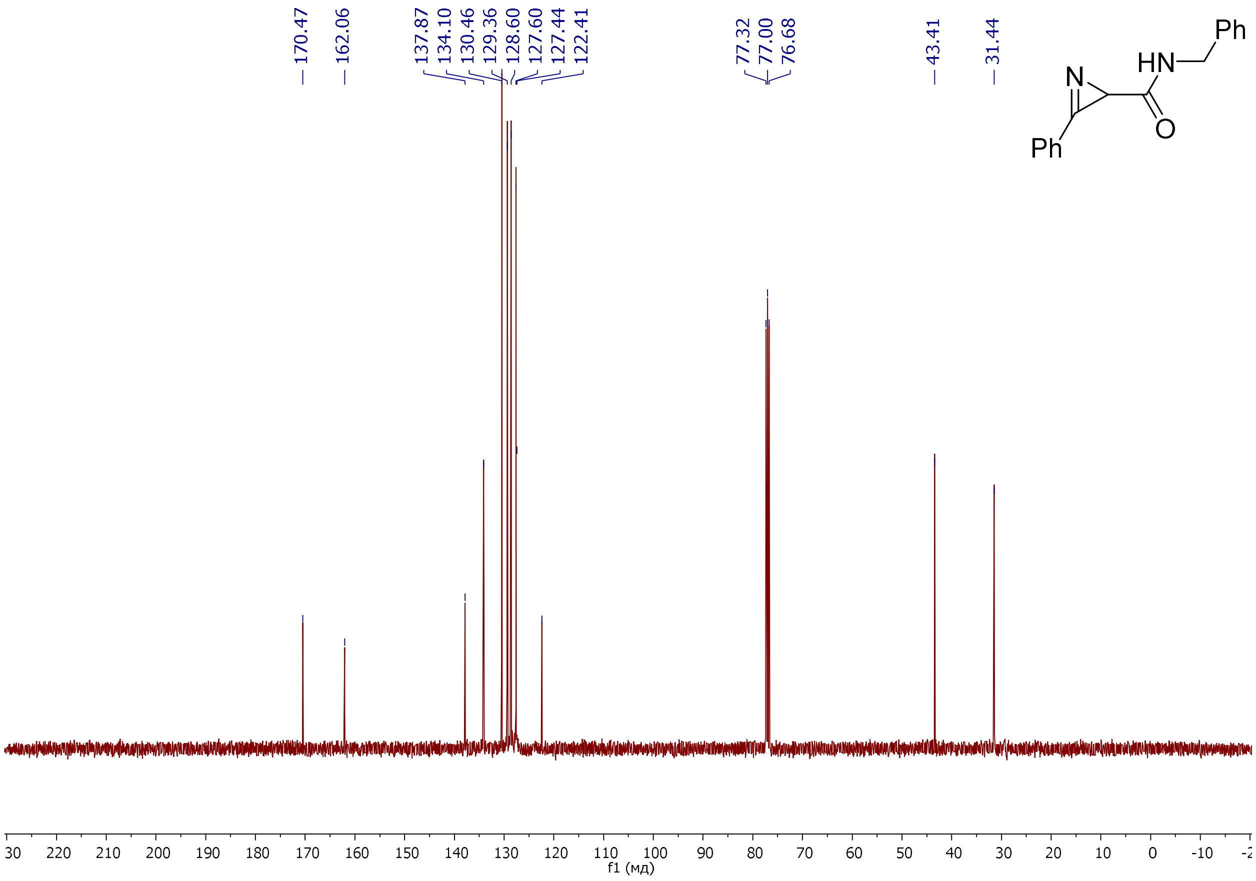

^{1}H NMR spectra ($CDCl_3$, 400 MHz) of compound **7i**

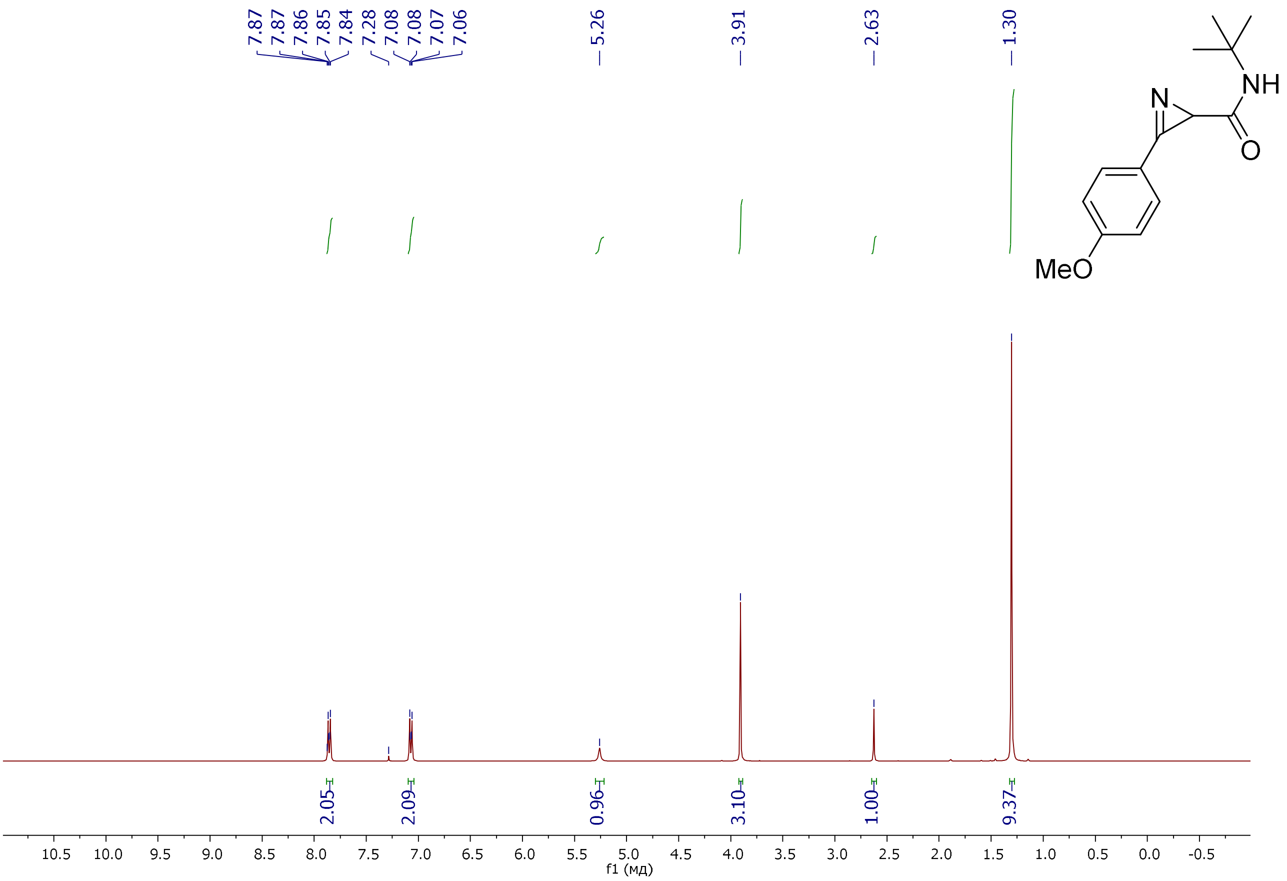

$^{13}C\{^{1}H\}$ NMR spectra ($CDCl_3$, 100 MHz) of compound **7i**

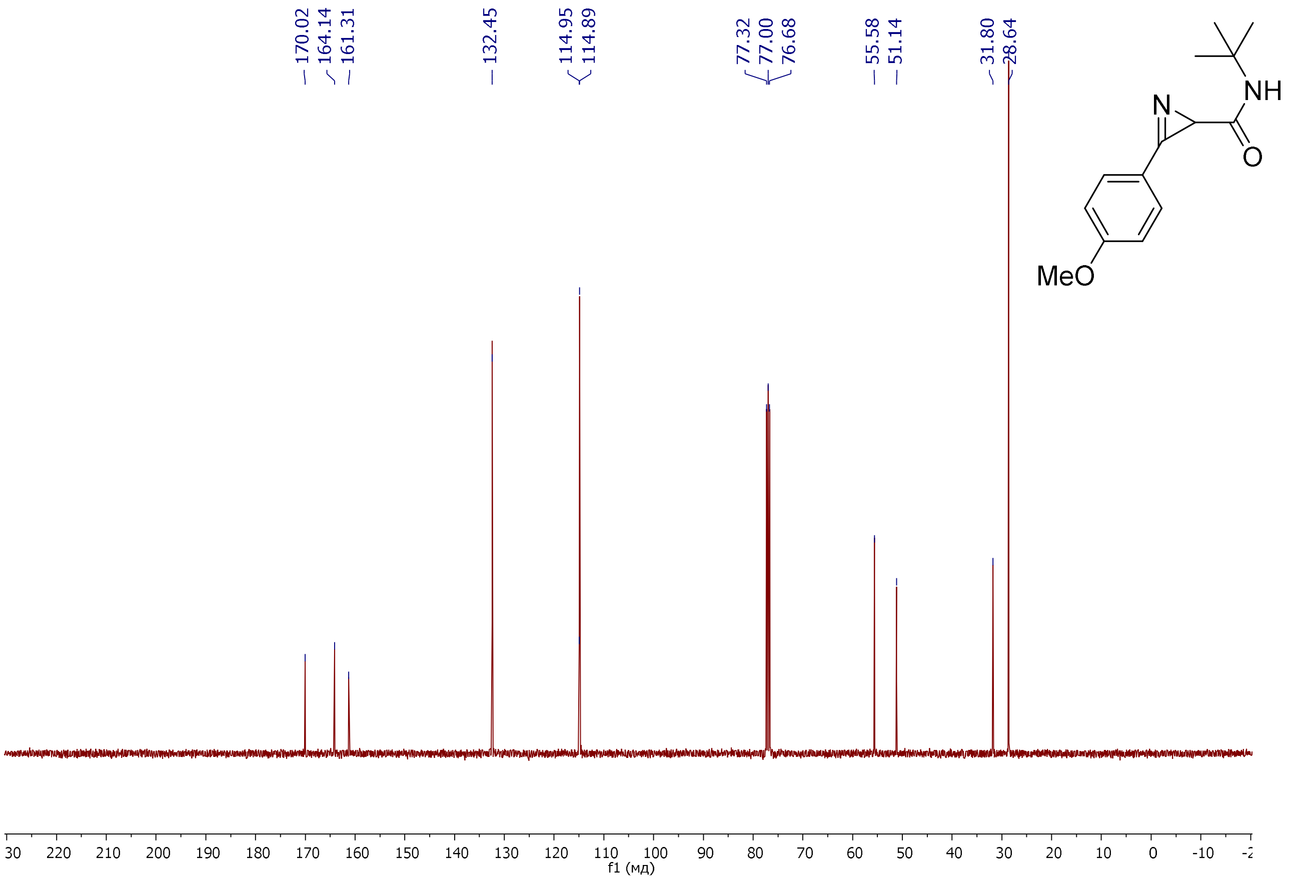

^{1}H NMR spectra ($CDCl_3$, 400 MHz) of compound **7j**

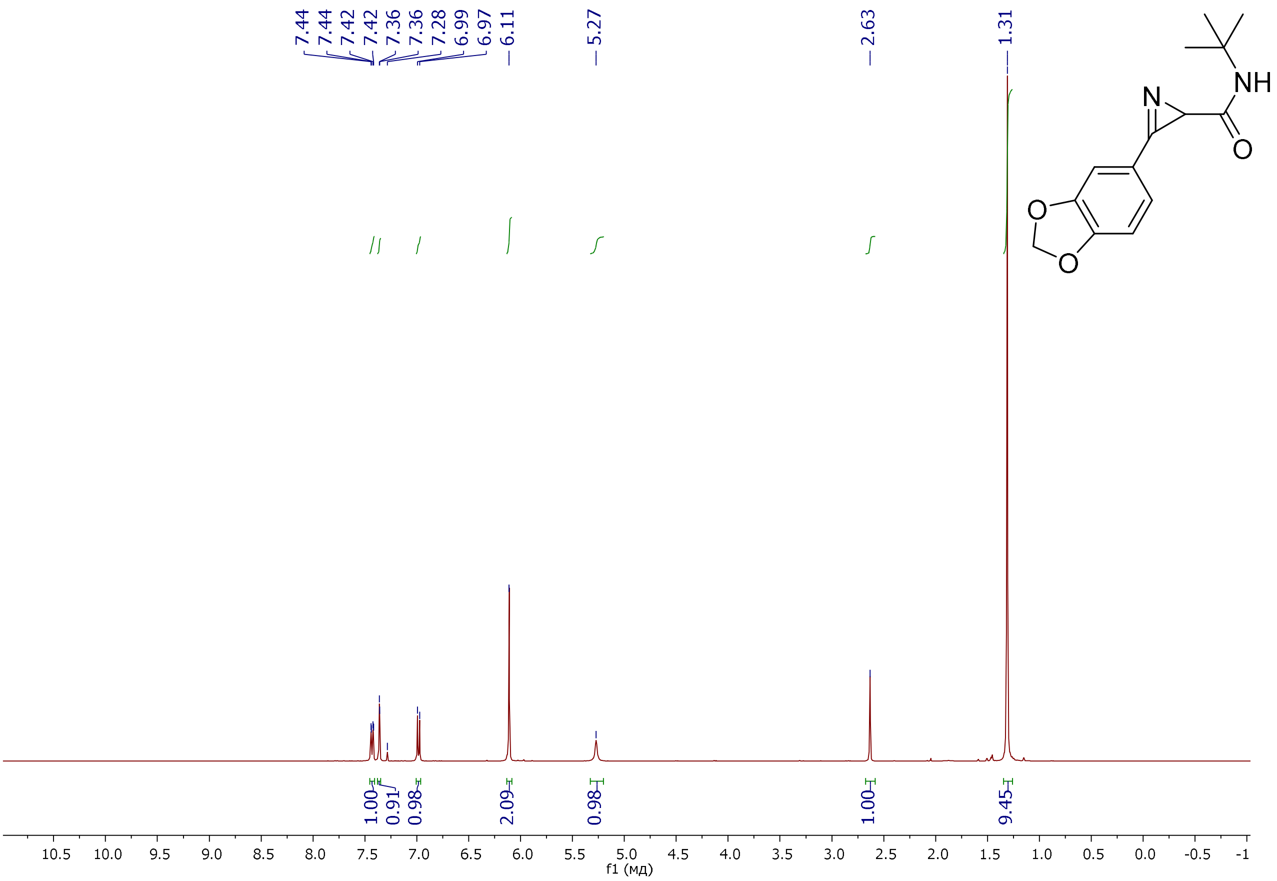

$^{13}C\{^{1}H\}$ NMR spectra ($CDCl_3$, 100 MHz) of compound **7j**

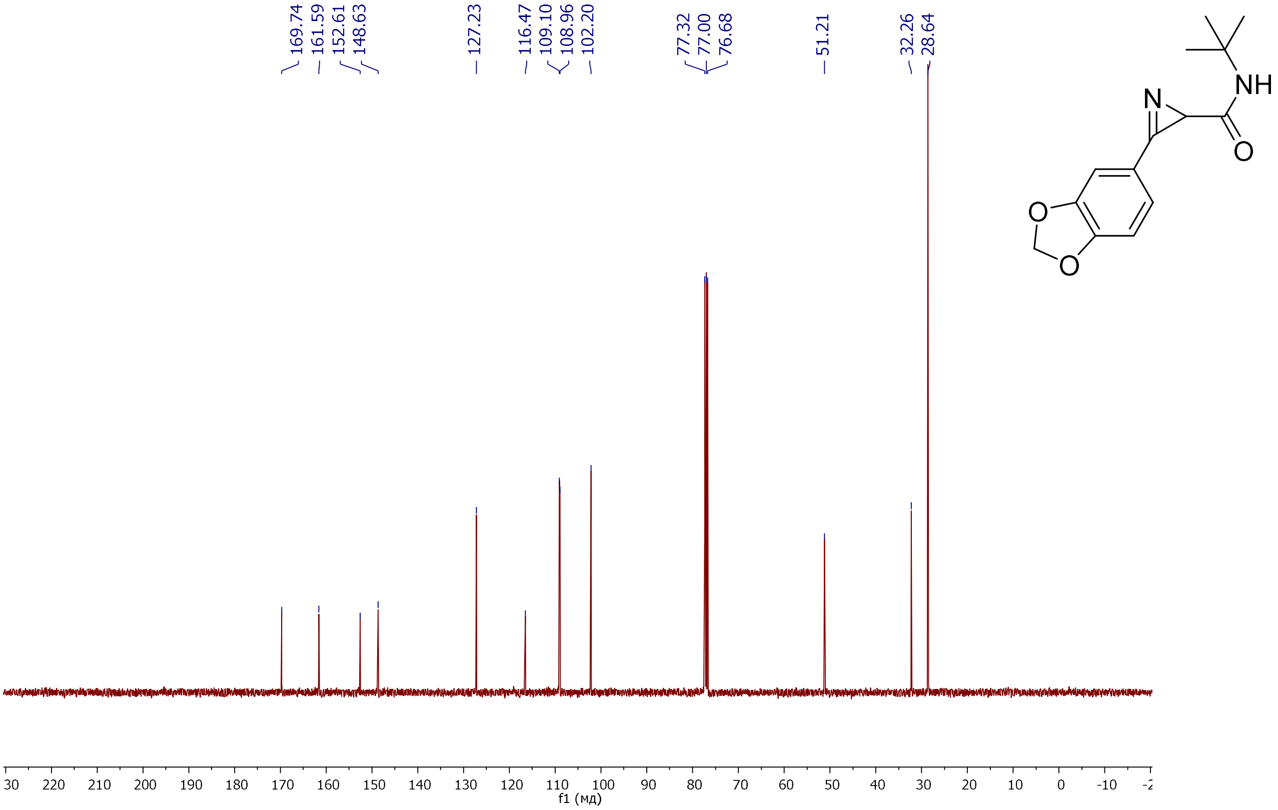

^{1}H NMR spectra ($CDCl_3$, 400 MHz) of compound **7k**

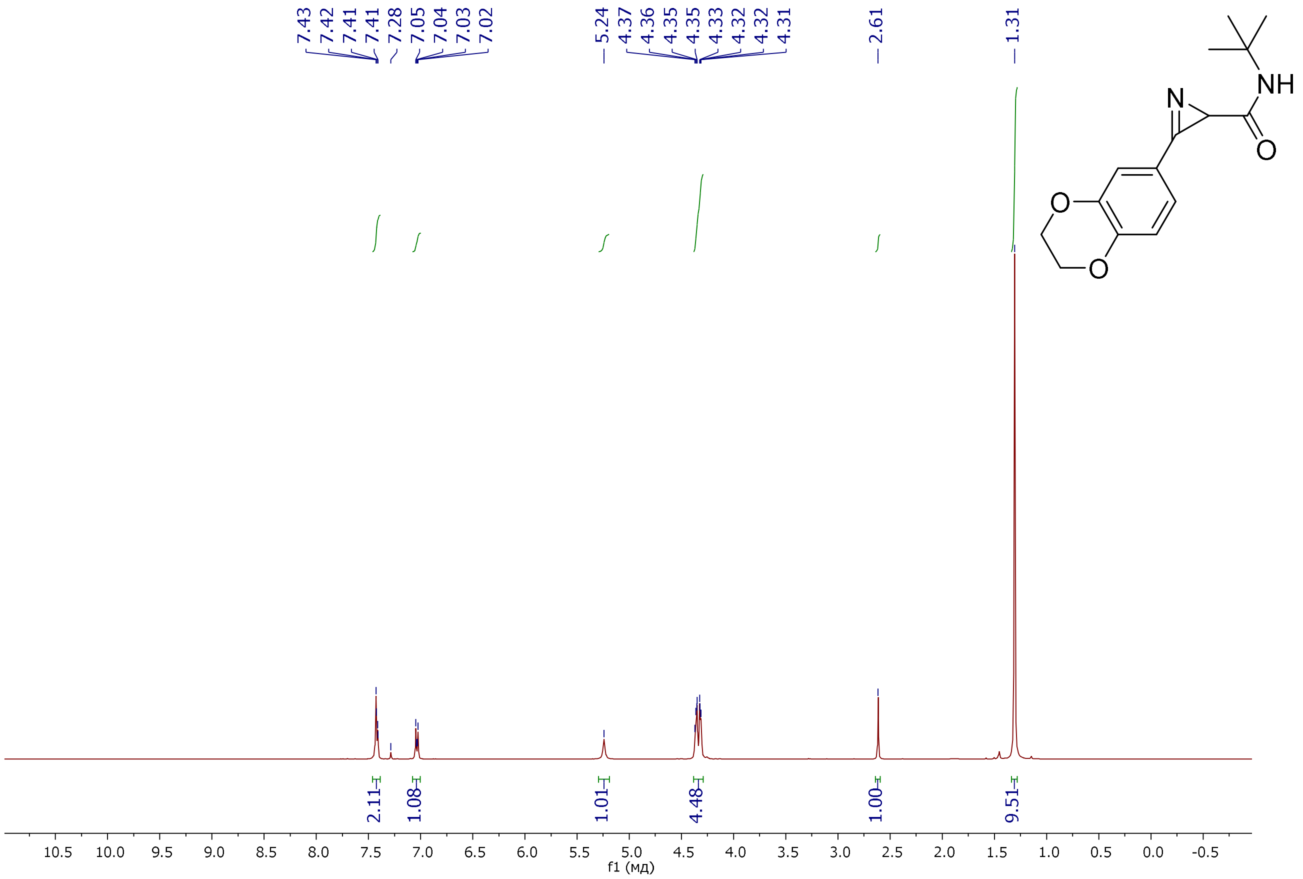

$^{13}C\{^{1}H\}$ NMR spectra ($CDCl_3$, 100 MHz) of compound **7k**

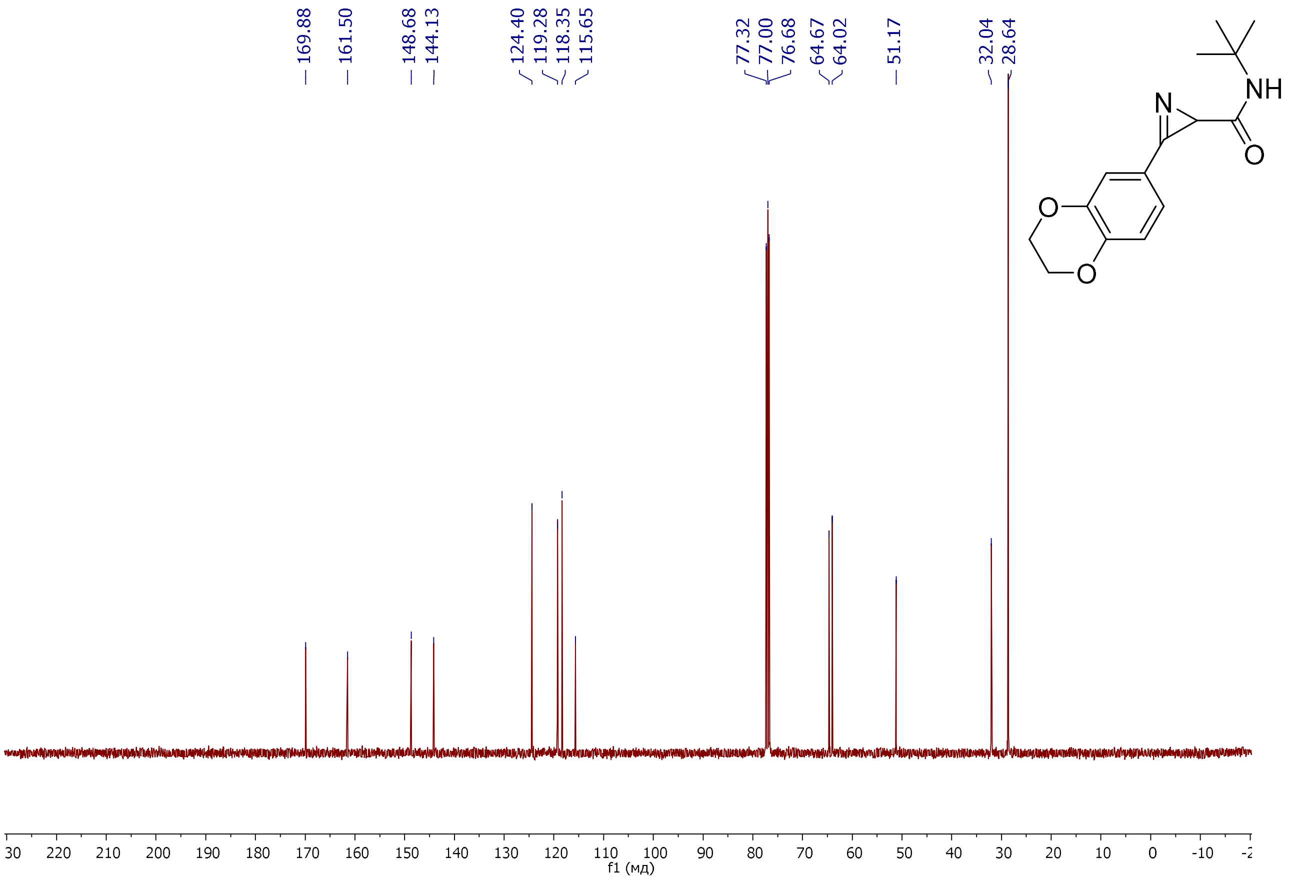

^{1}H NMR spectra ($CDCl_3$, 400 MHz) of compound **7l**

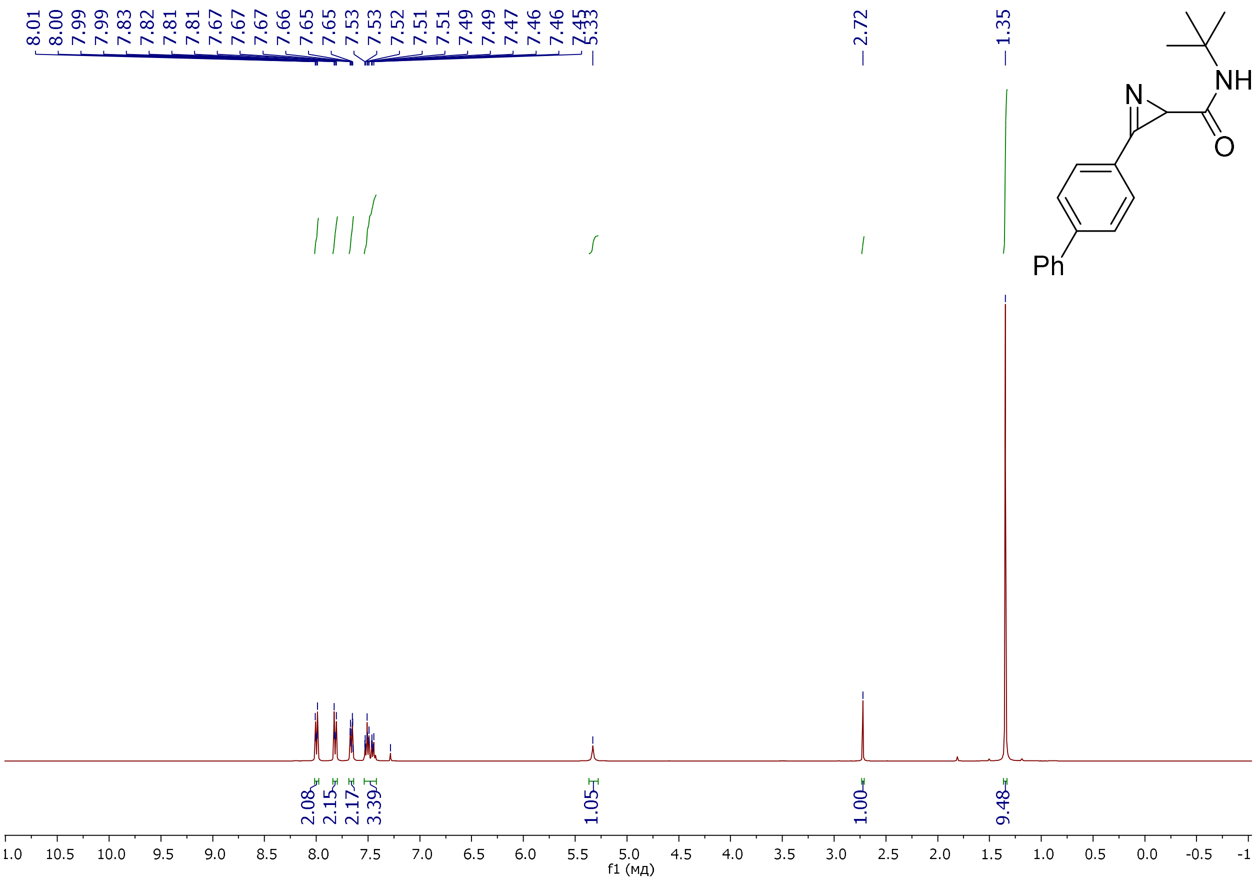

$^{13}C\{^{1}H\}$ NMR spectra ($CDCl_3$, 100 MHz) of compound **7l**

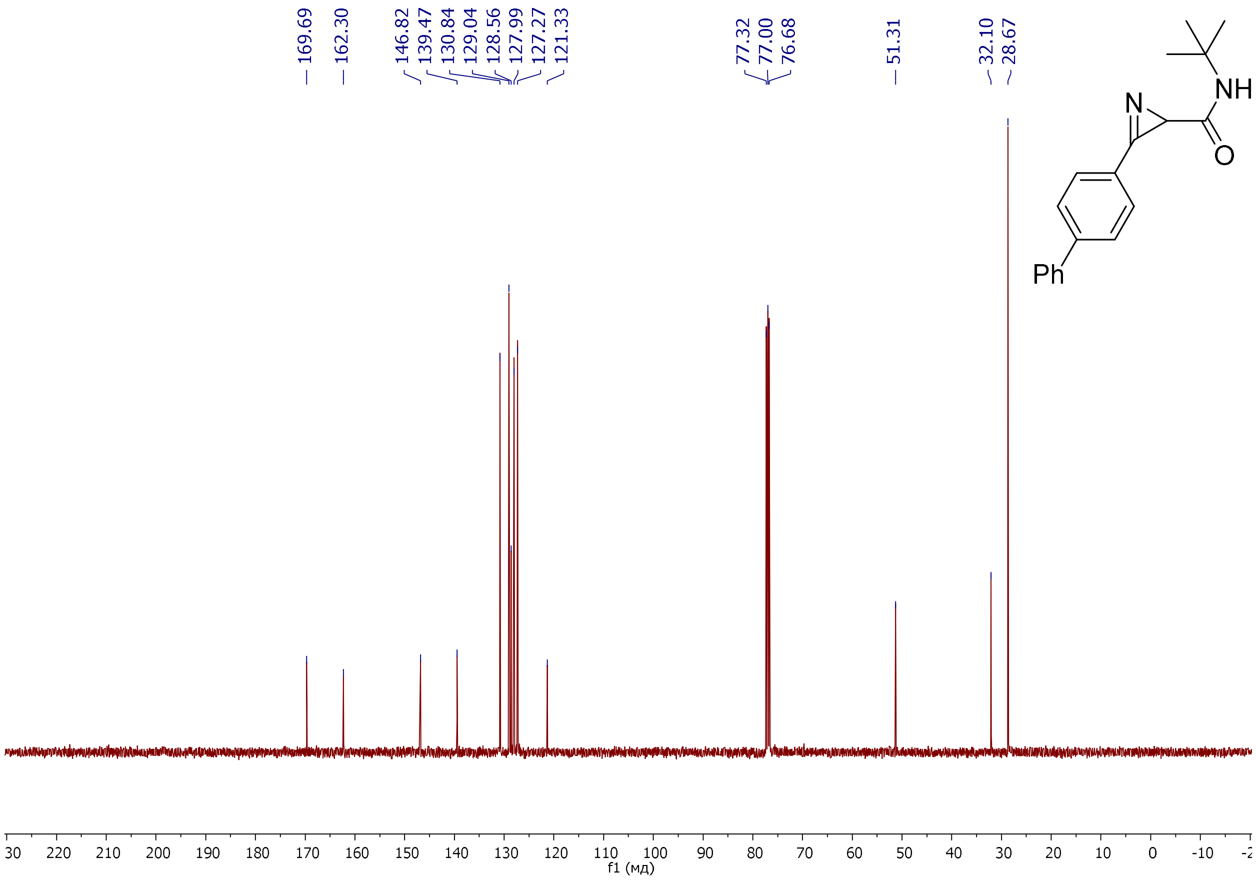

^{1}H NMR spectra ($CDCl_3$, 400 MHz) of compound **7m**

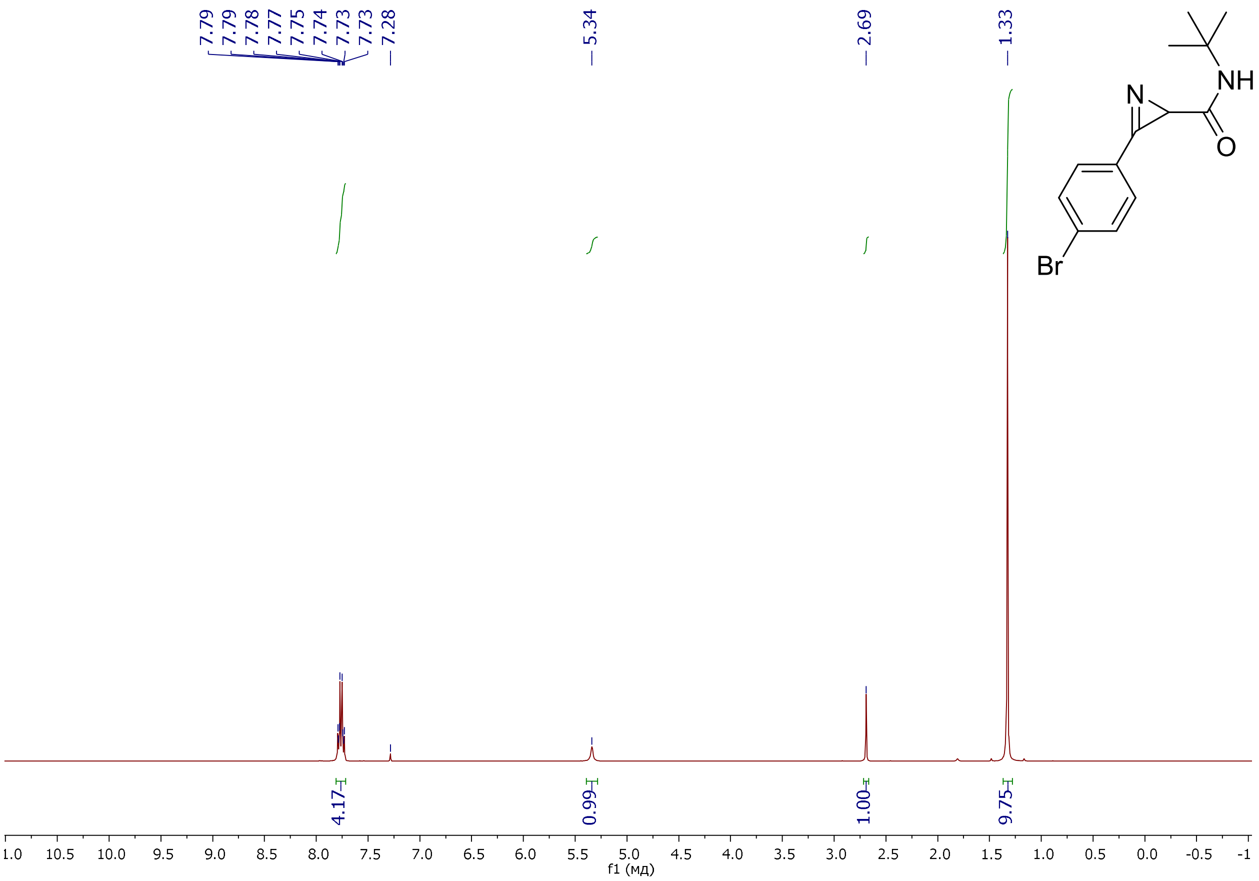

^{13}C{^{1}H} NMR spectra ($CDCl_3$, 100 MHz) of compound **7m**

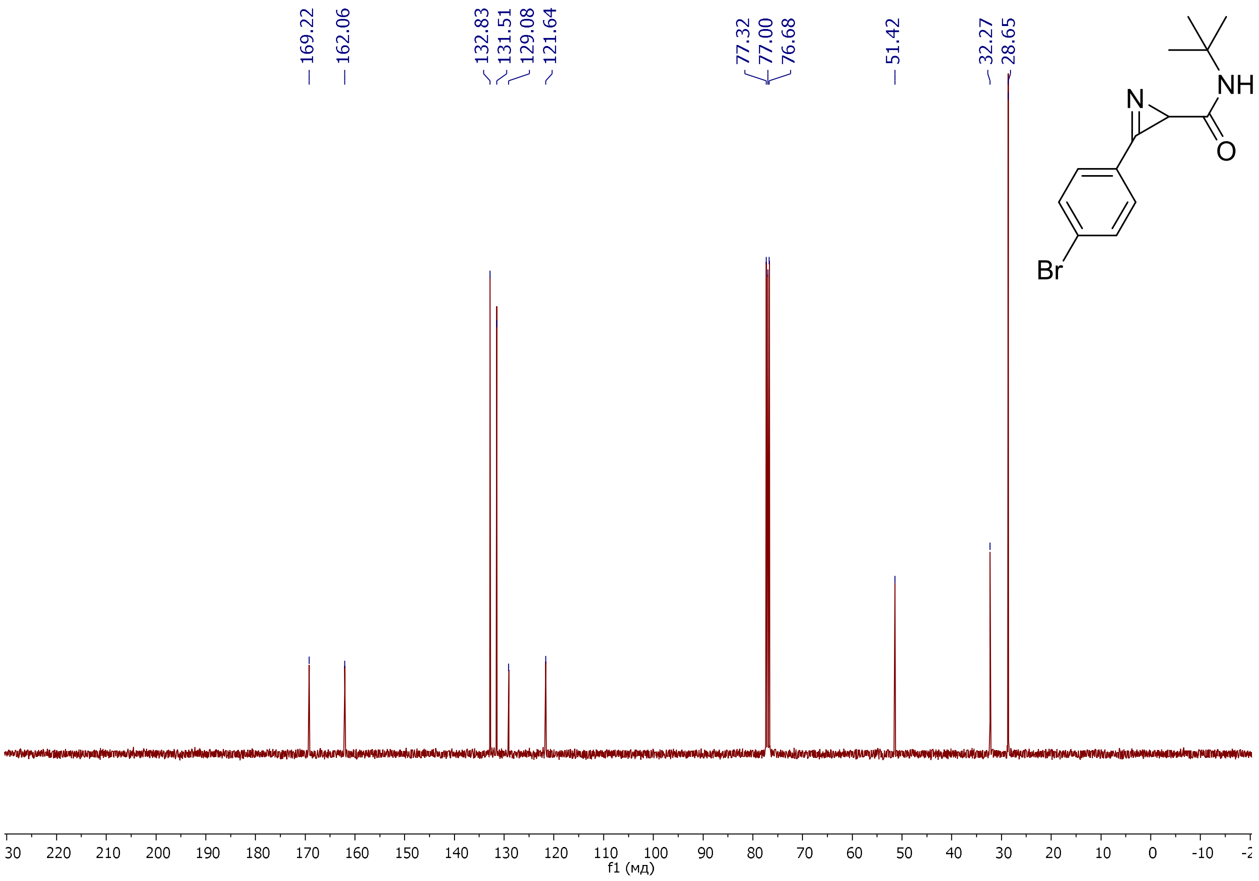

^{1}H NMR spectra ($CDCl_3$, 400 MHz) of compound **7n**

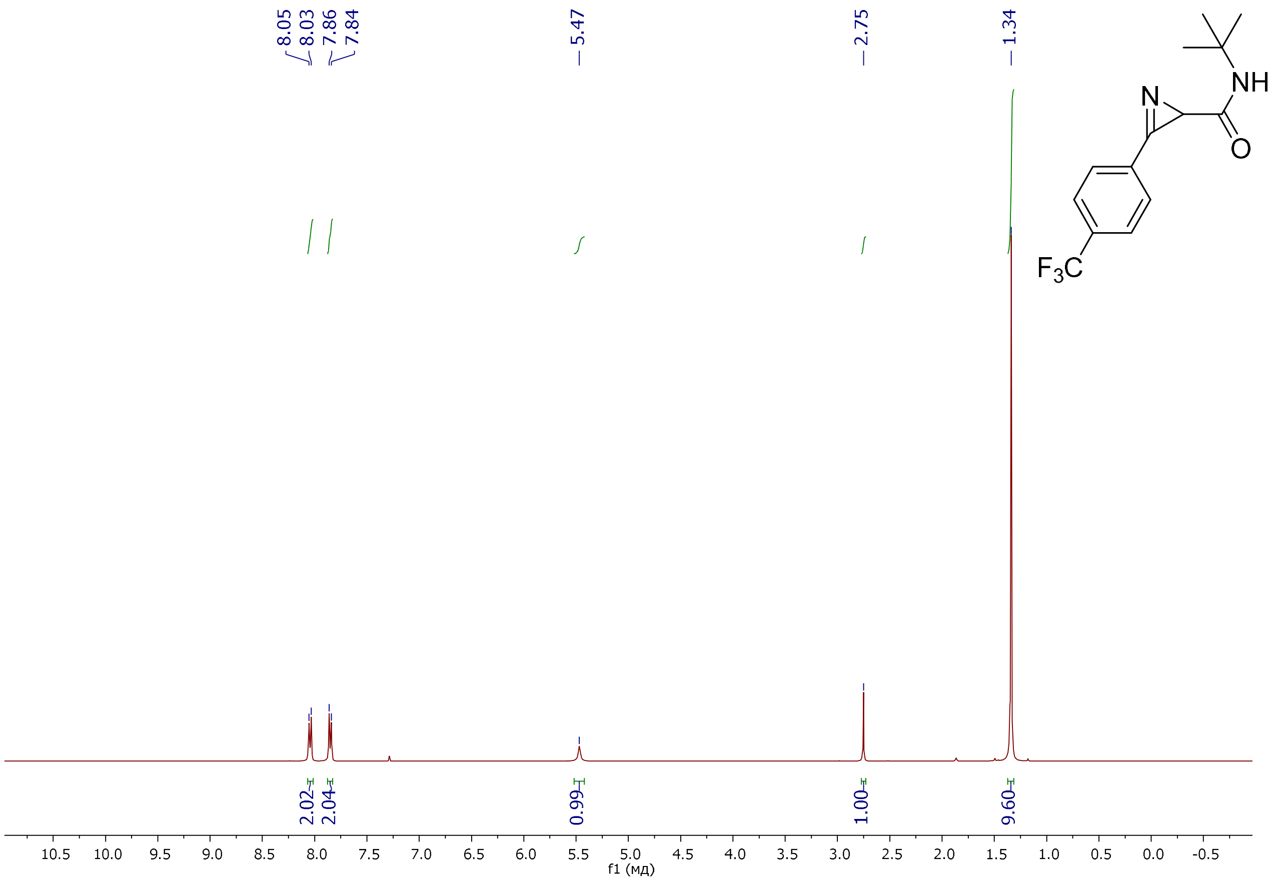

^{13}C{^{1}H} NMR spectra ($CDCl_3$, 100 MHz) of compound **7n**

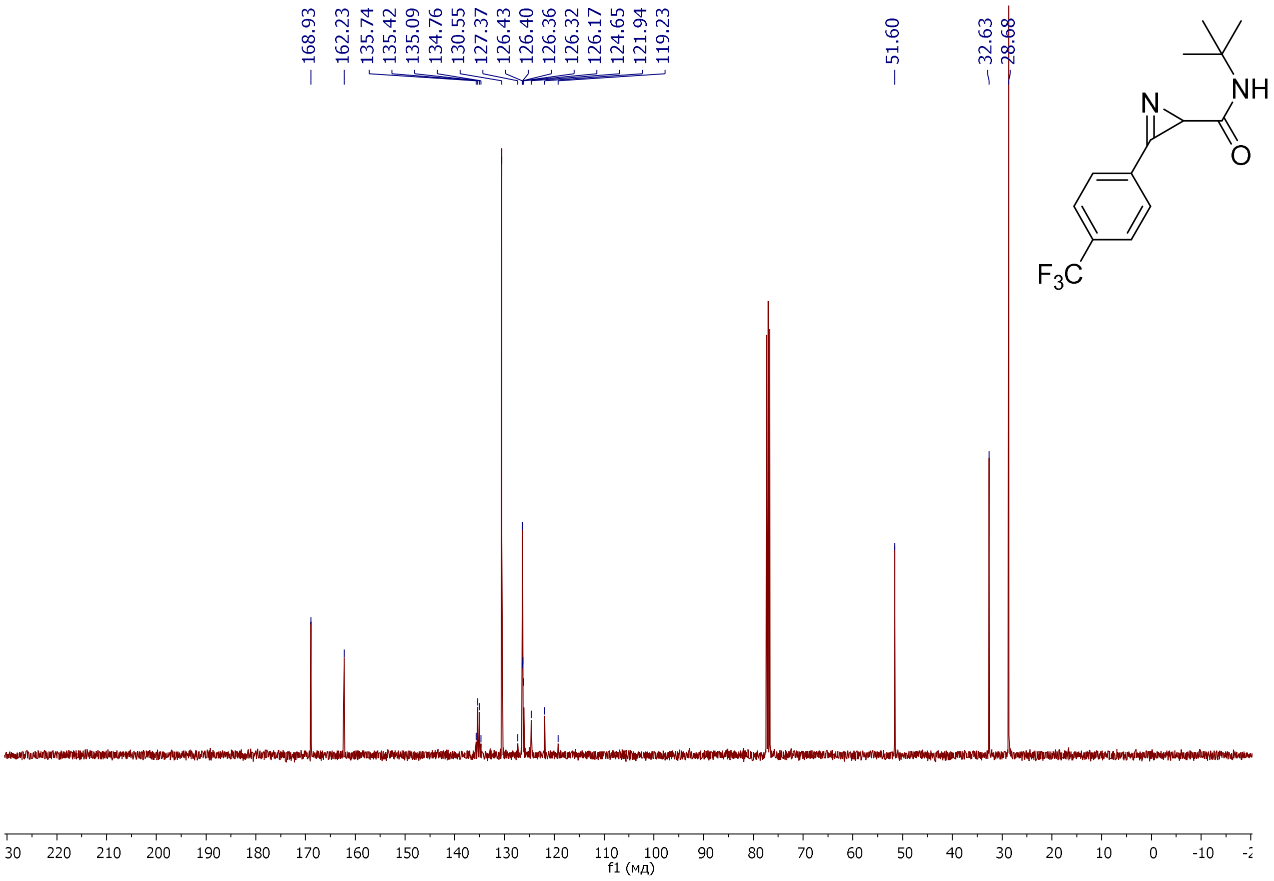

^{1}H NMR spectra ($CDCl_3$, 400 MHz) of compound **7o**

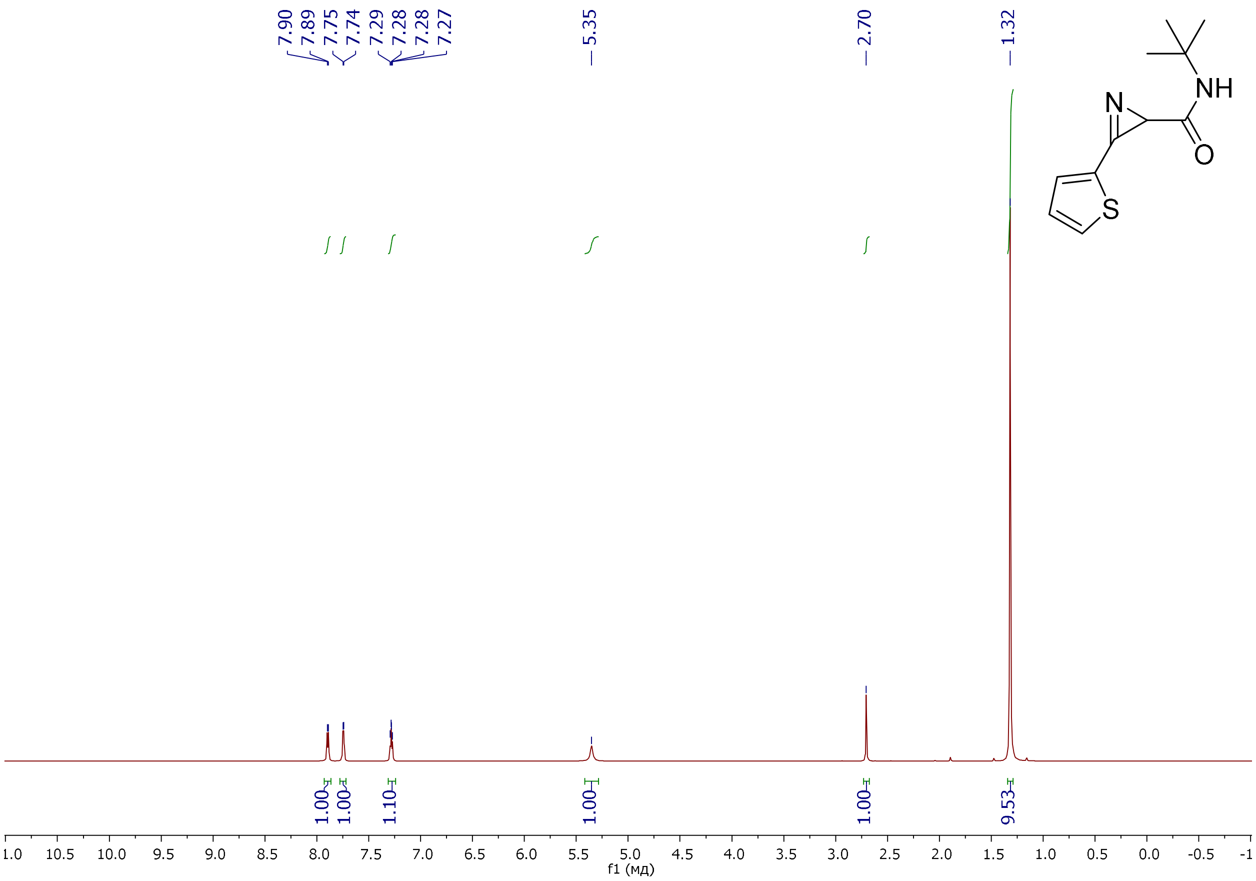

^{13}C{^{1}H} NMR spectra ($CDCl_3$, 100 MHz) of compound **7o**

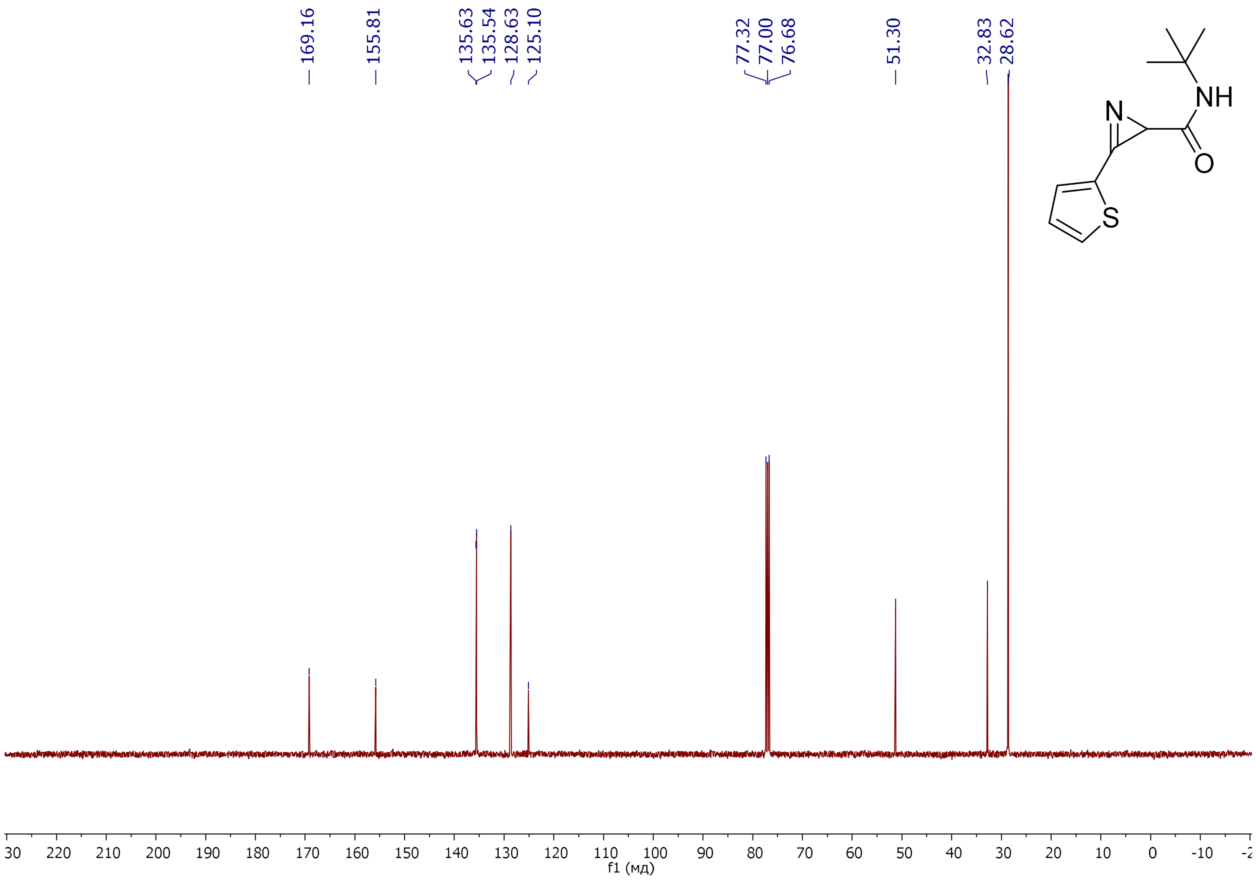

^{1}H NMR spectra ($CDCl_3$, 400 MHz) of compound **7p**

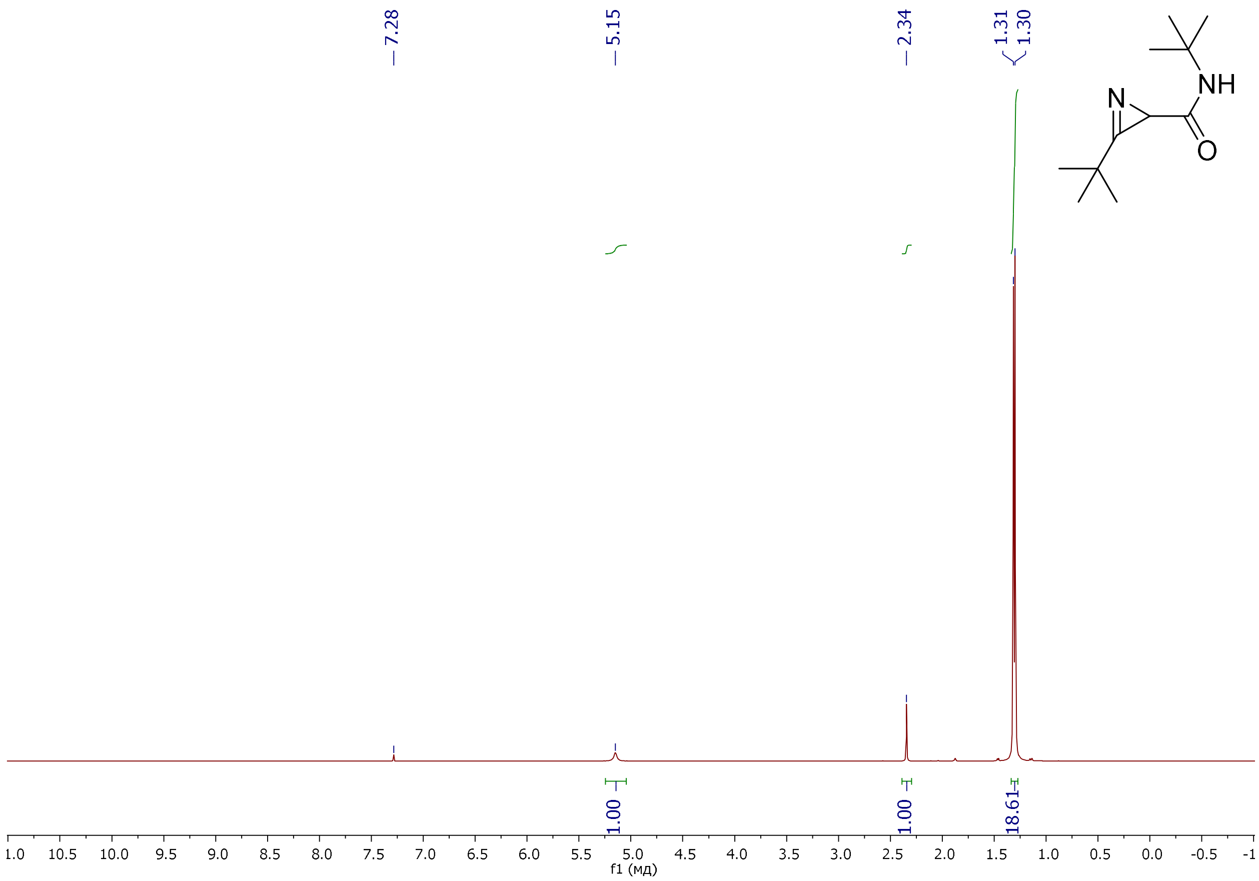

^{13}C{^{1}H} NMR spectra ($CDCl_3$, 100 MHz) of compound **7p**

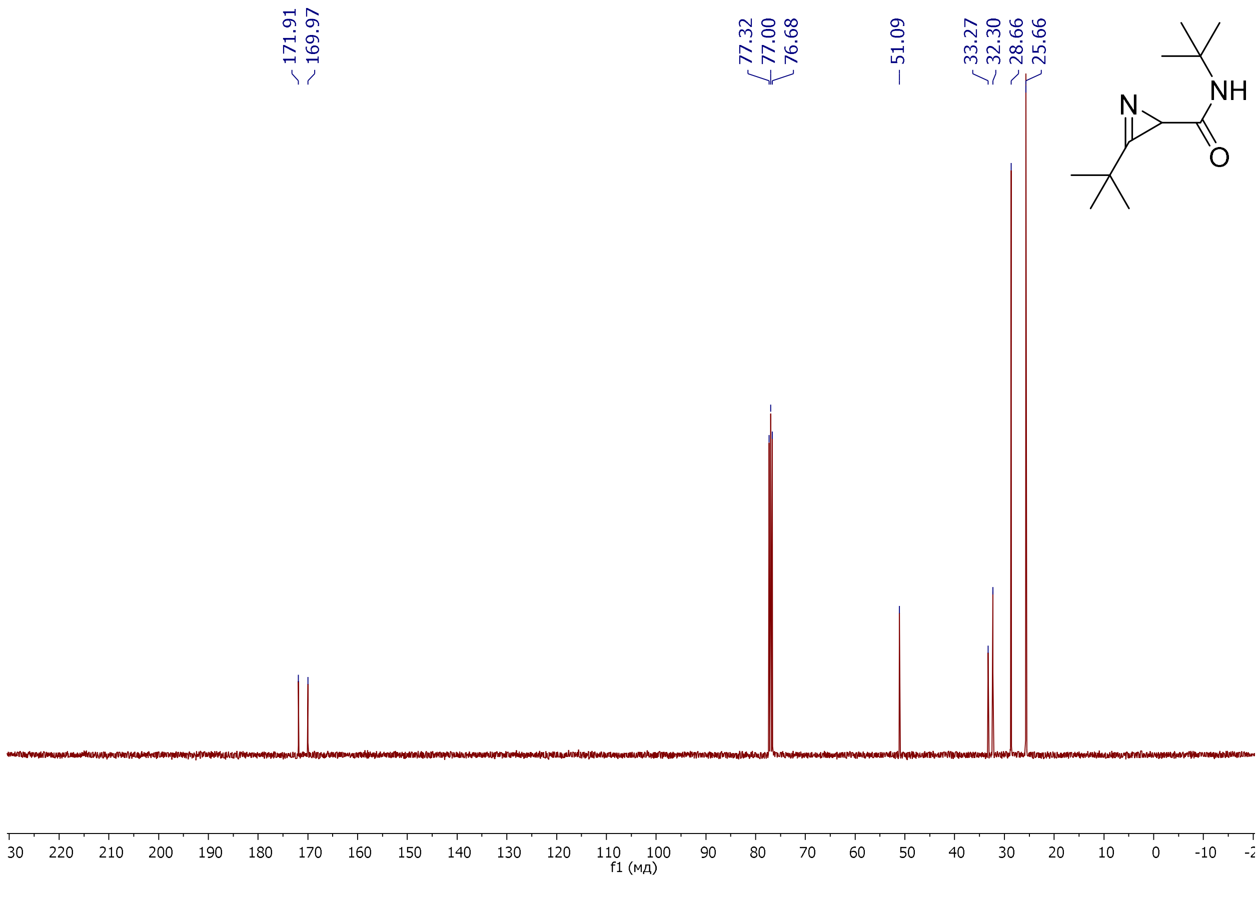

^1H NMR spectra ($CDCl_3$, 400 MHz) of compound **7q**

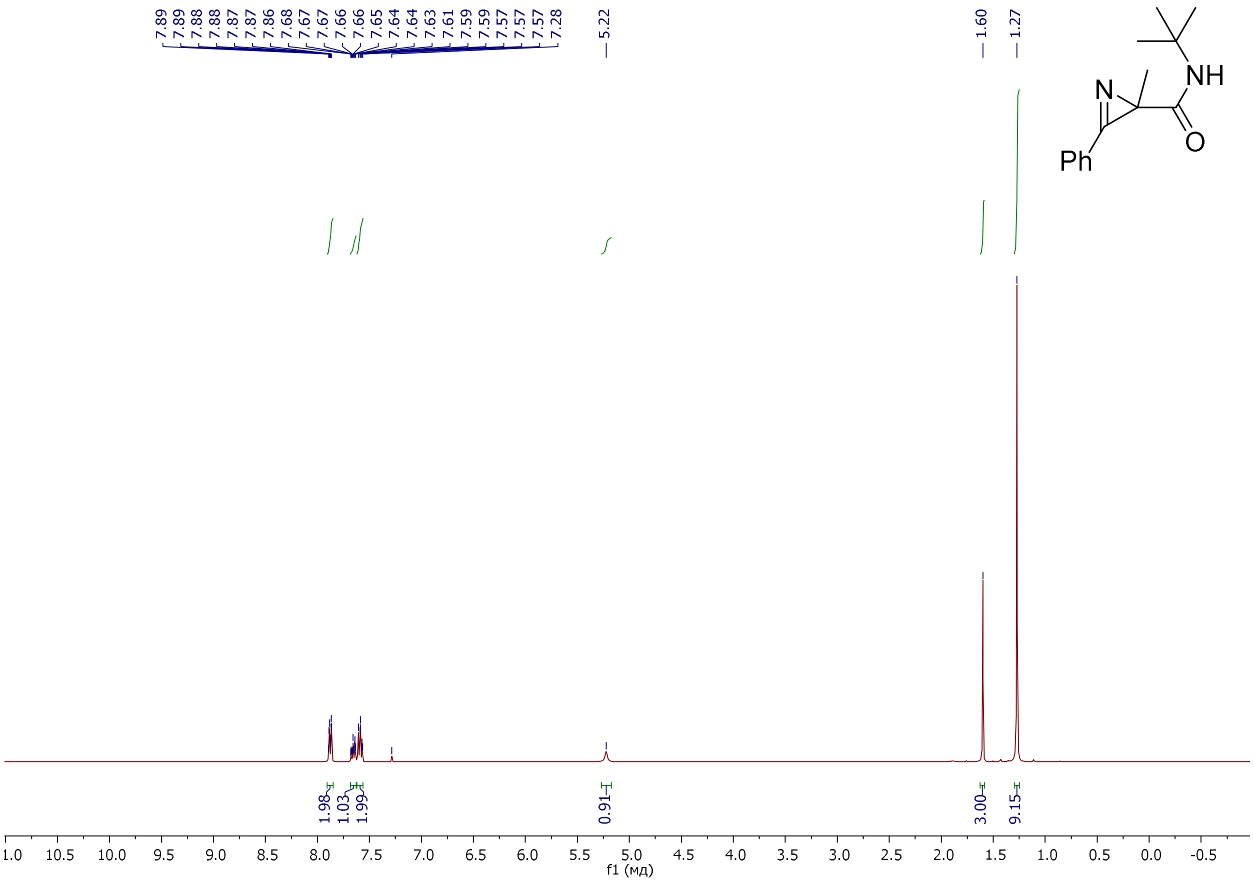

$^{13}C\{^1H\}$ NMR spectra ($CDCl_3$, 100 MHz) of compound **7q**

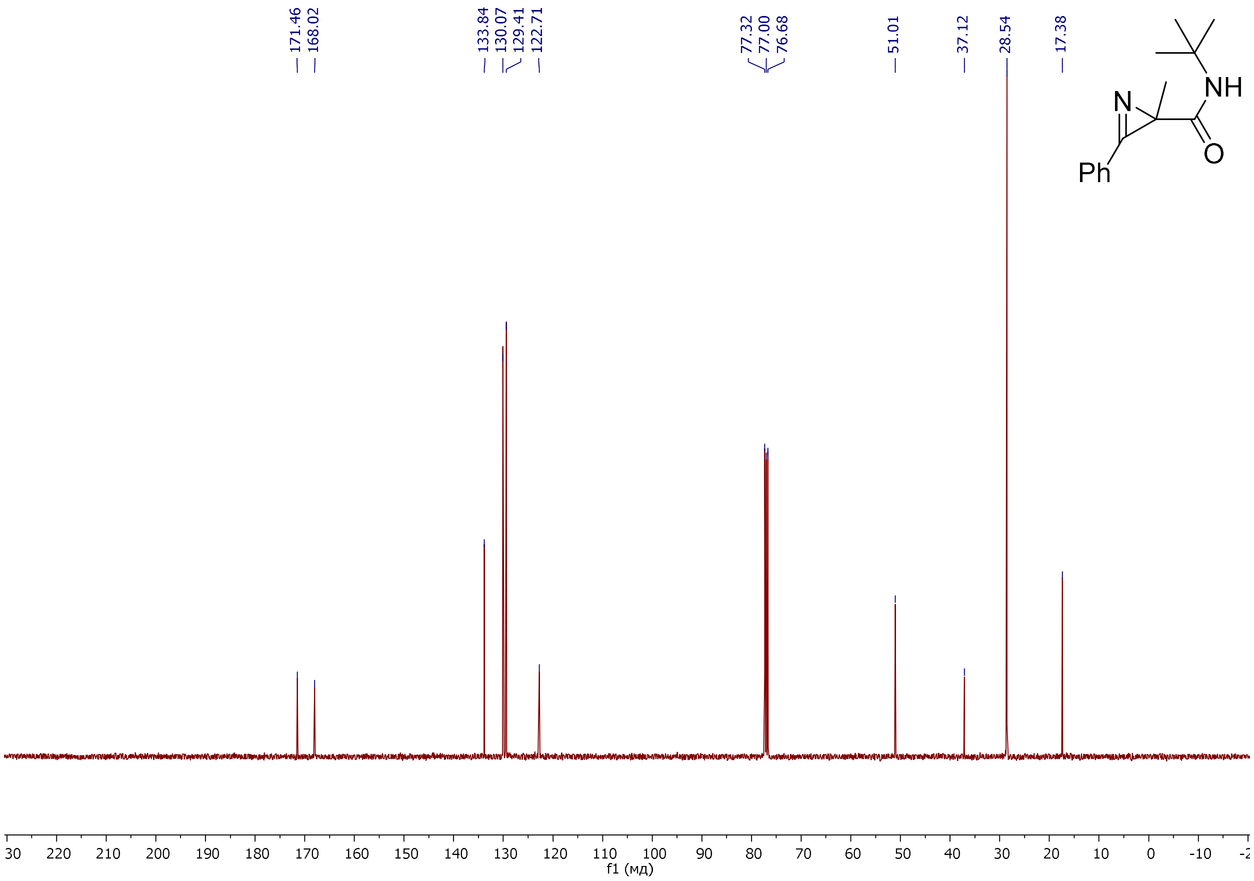

^{1}H NMR spectra (C_6D_6, 400 MHz) of compound **10a**

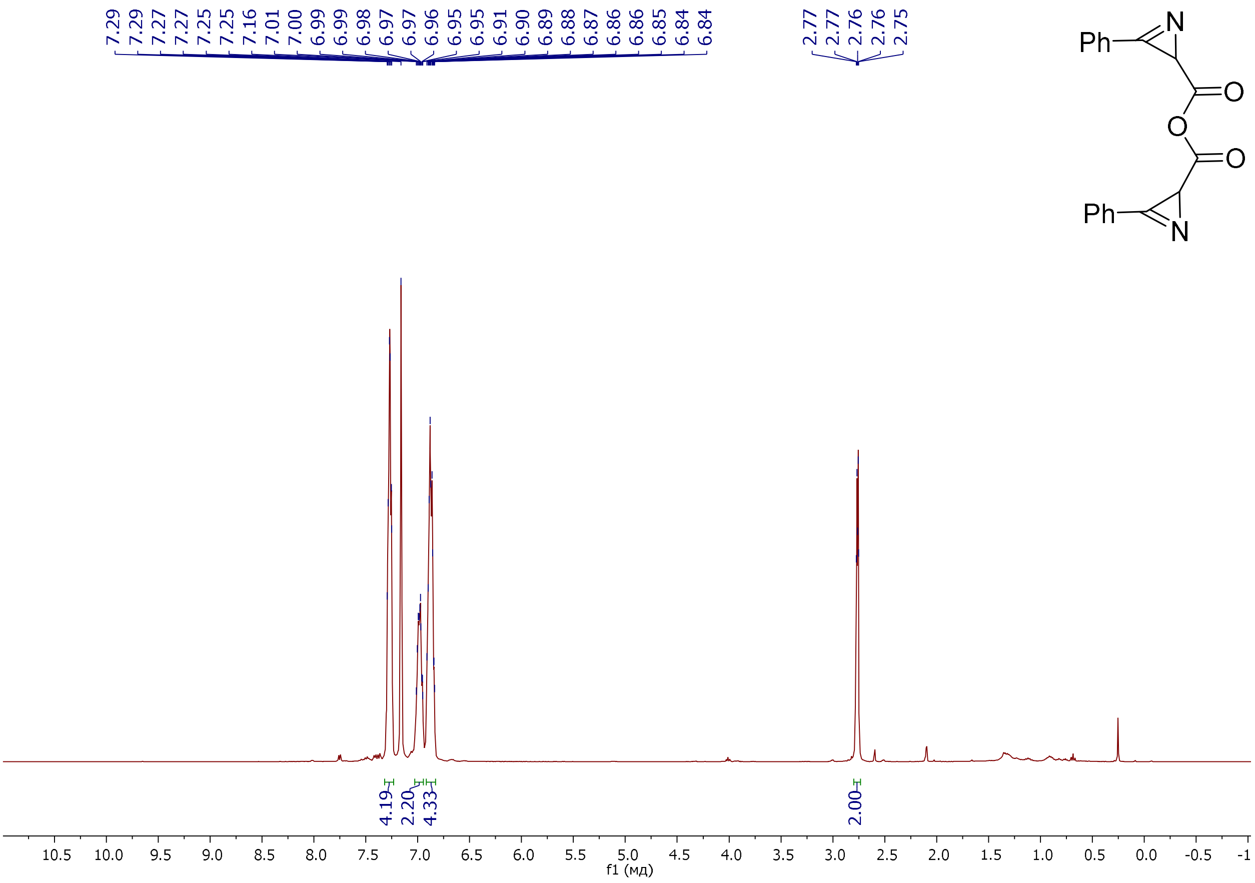

^{13}C{^{1}H} NMR spectra (C_6D_6, 100 MHz) of compound **10a**

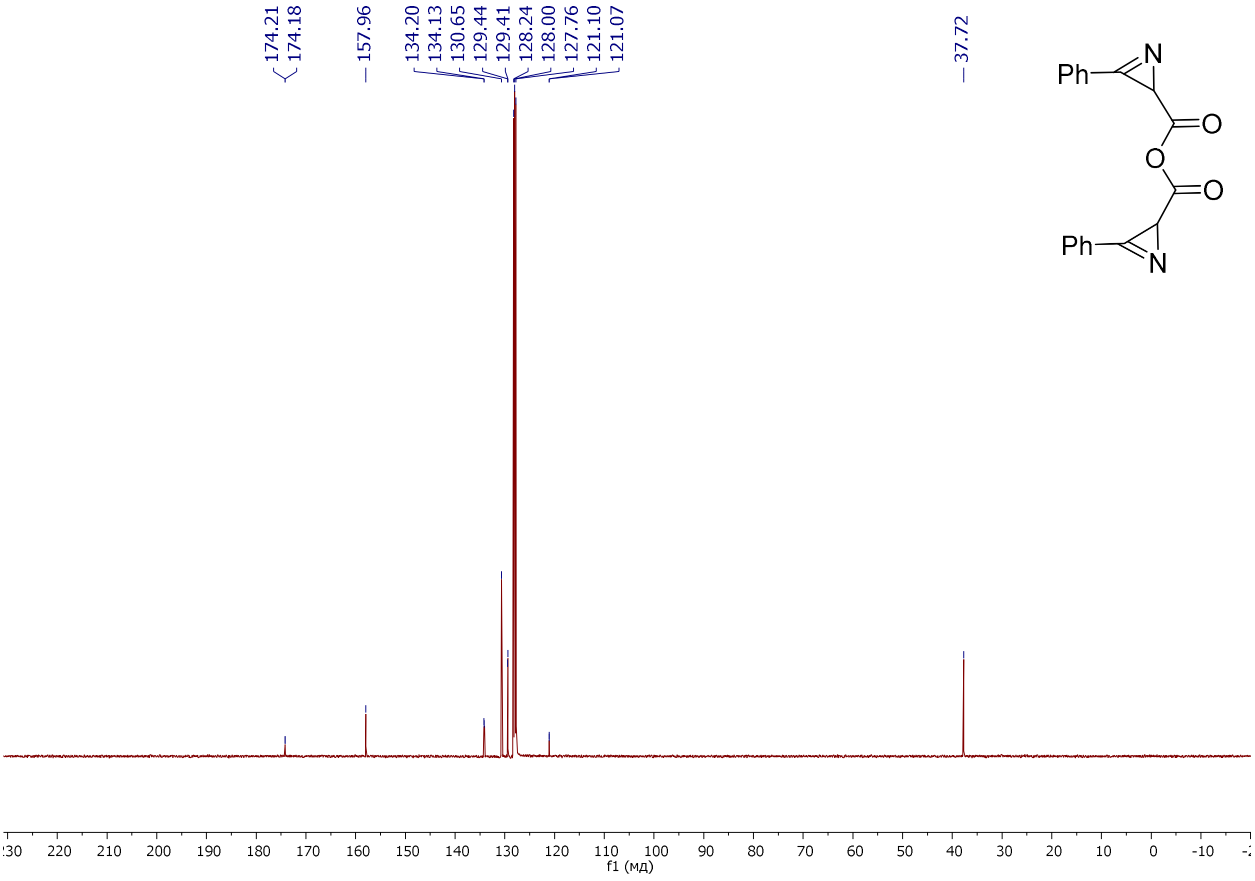

^{1}H NMR spectra (C_6D_6, 400 MHz) of compound **10b**

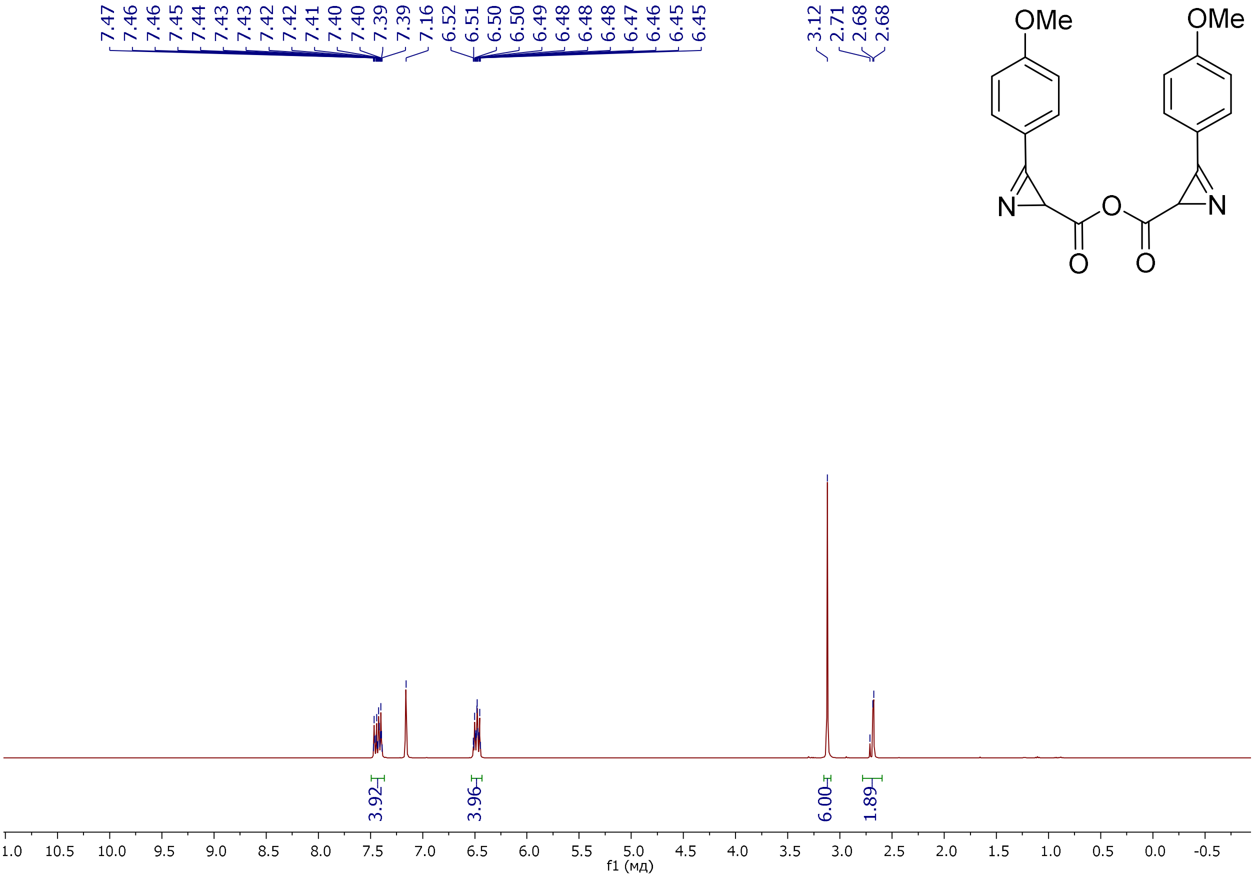

^{13}C{^{1}H} NMR spectra ($CDCl_3$, 100 MHz) of compound **10b**

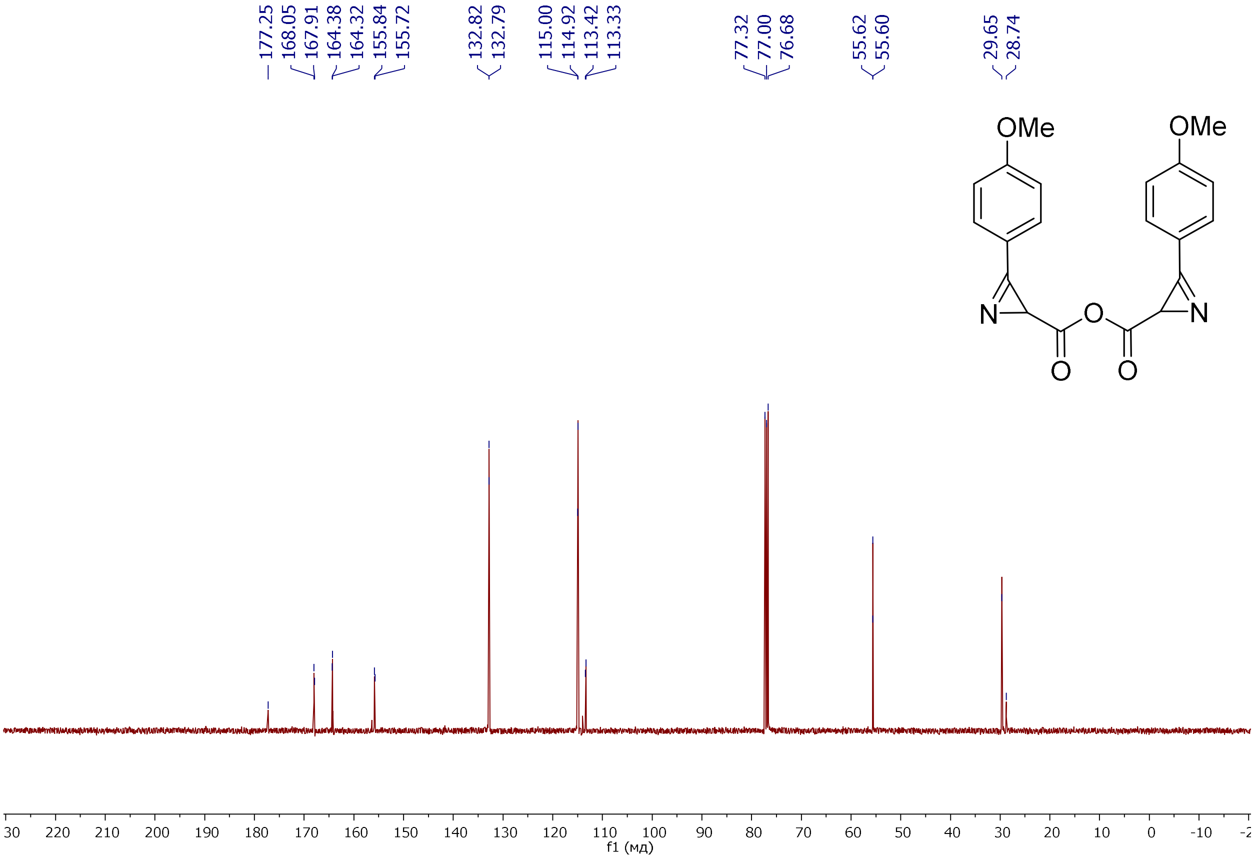

^{1}H NMR spectra ($CDCl_3$, 400 MHz) of compound **14a**

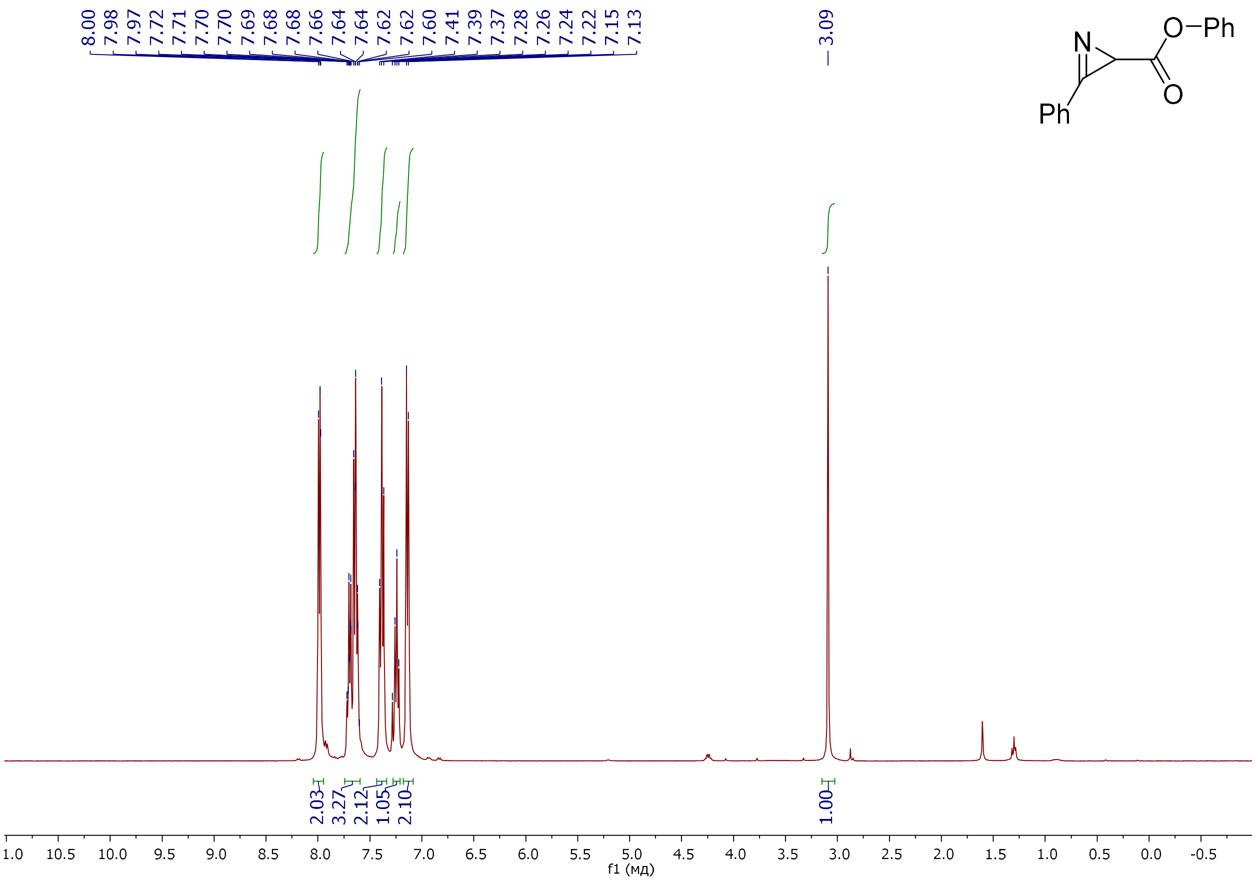

^{13}C{^{1}H} NMR spectra ($CDCl_3$, 100 MHz) of compound **14a**

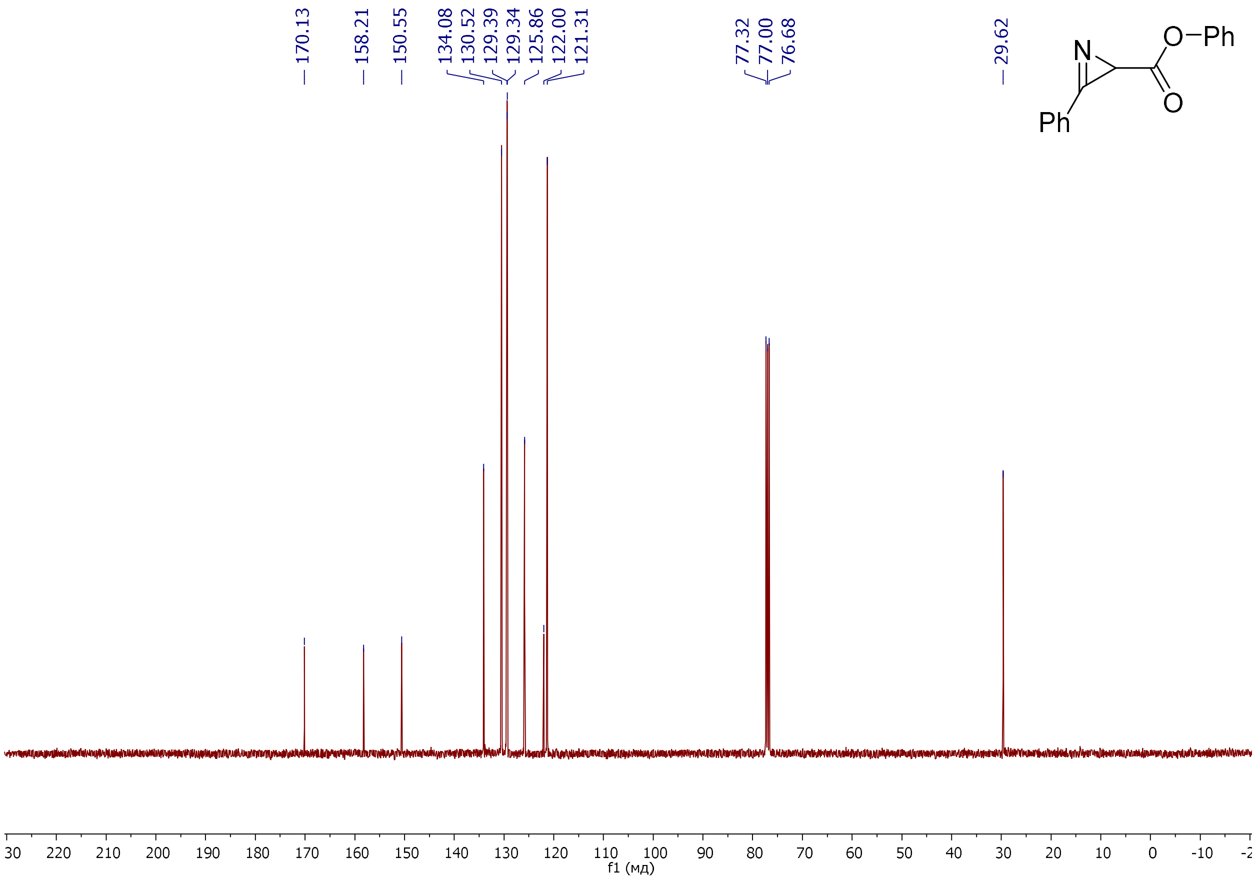

^{1}H NMR spectra ($CDCl_3$, 400 MHz) of compound **14b**

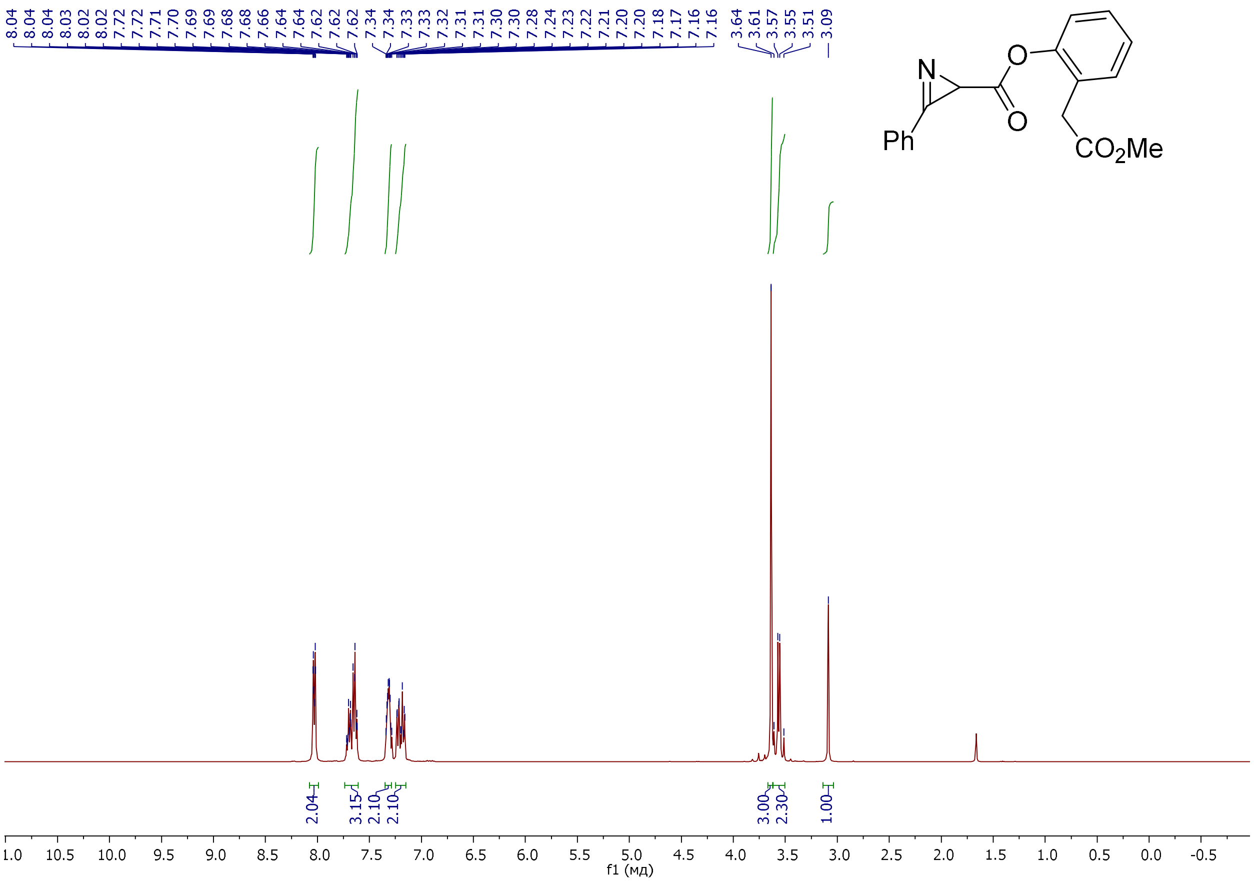

^{13}C{^{1}H} NMR spectra ($CDCl_3$, 100 MHz) of compound **14b**

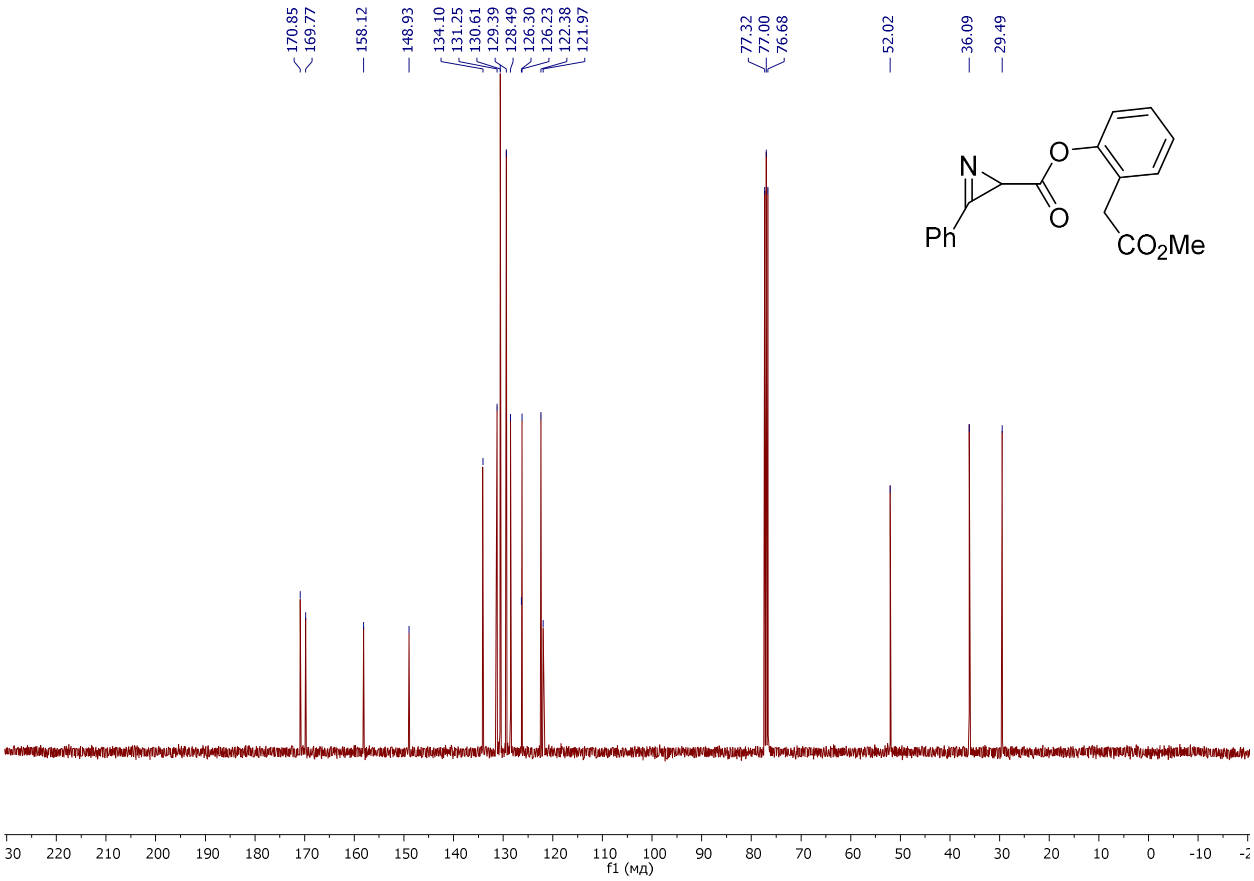

^{1}H NMR spectra ($CDCl_3$, 400 MHz) of compound **14c**

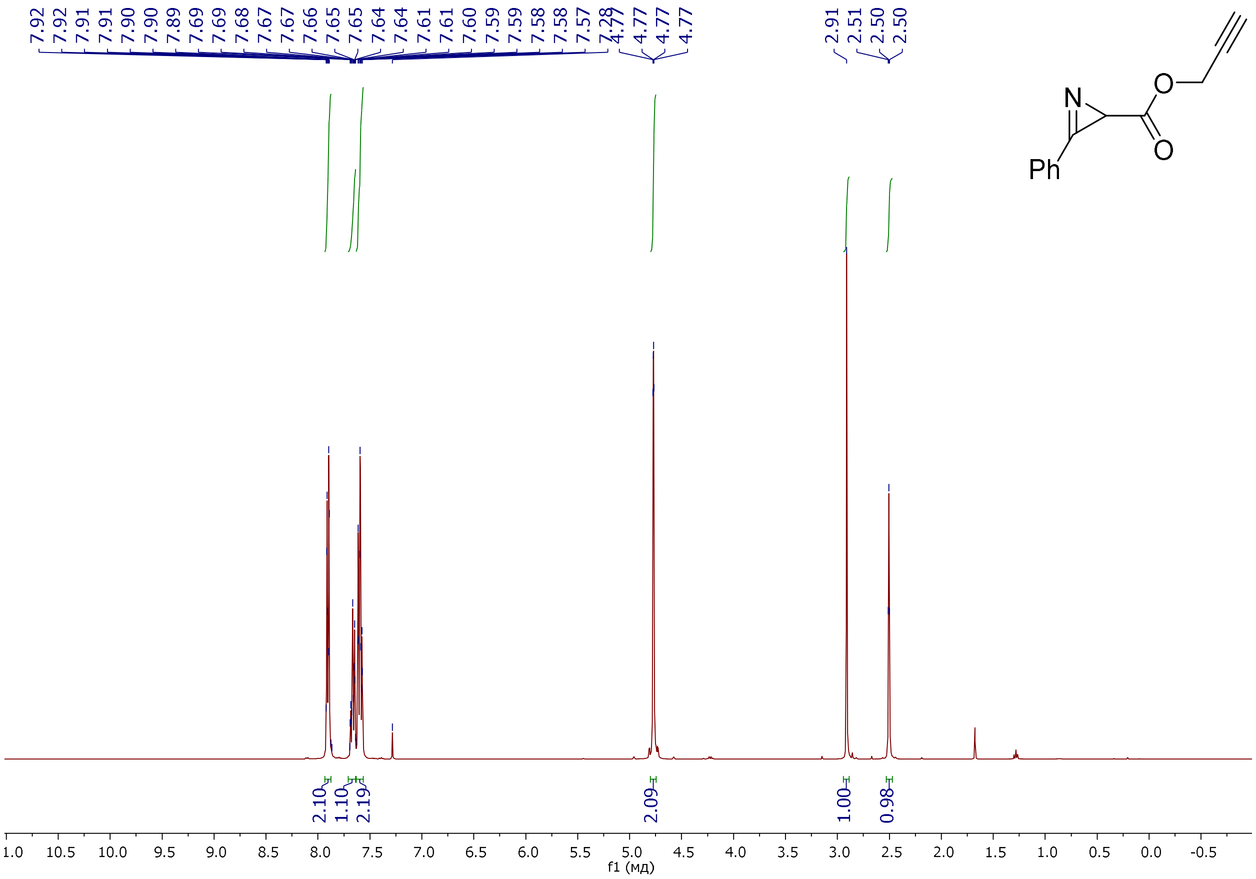

^{13}C{^{1}H} NMR spectra ($CDCl_3$, 100 MHz) of compound **14c**

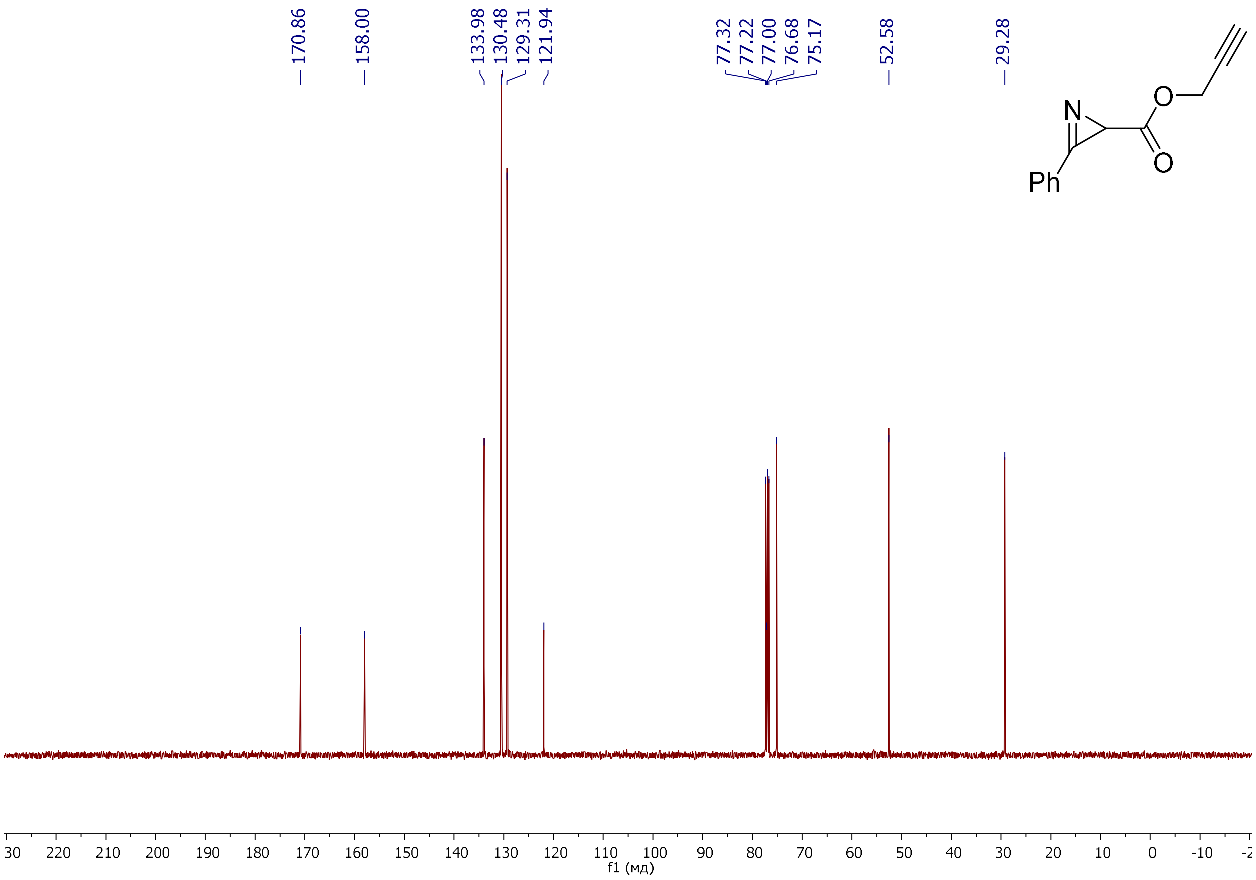

^{1}H NMR spectra ($CDCl_3$, 400 MHz) of compound **14d**

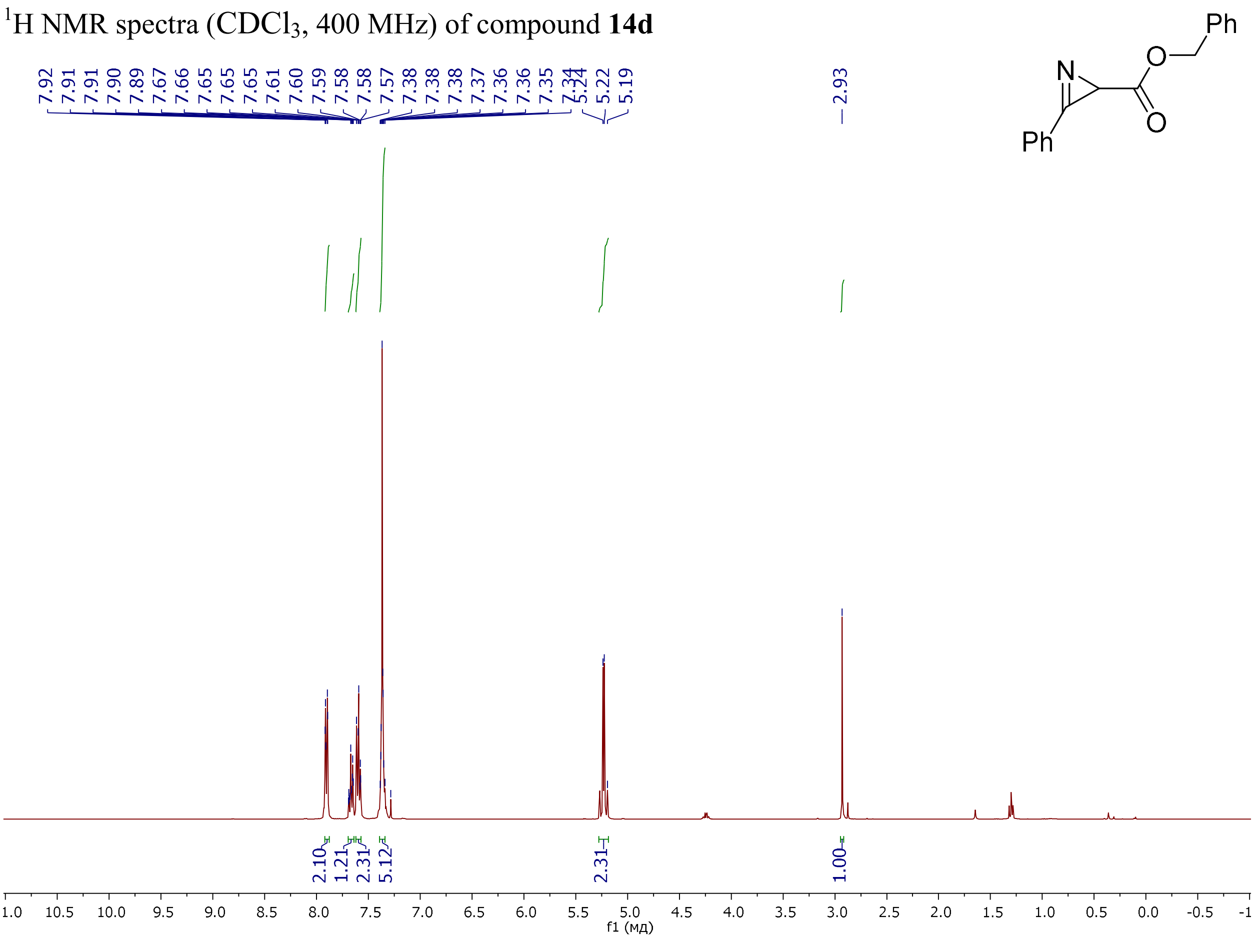

^{13}C{^{1}H} NMR spectra ($CDCl_3$, 100 MHz) of compound **14d**

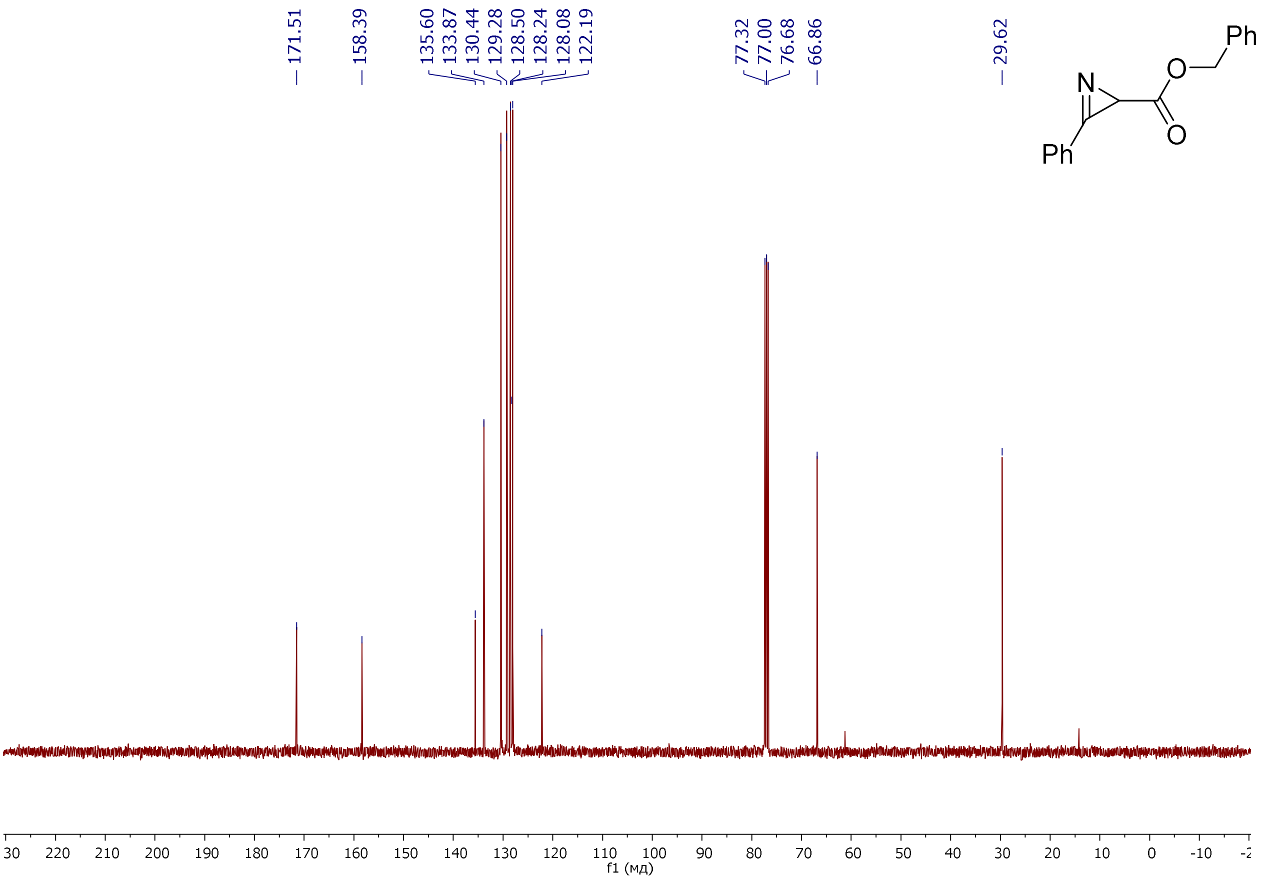

^{1}H NMR spectra ($CDCl_3$, 400 MHz) of compound **15a**

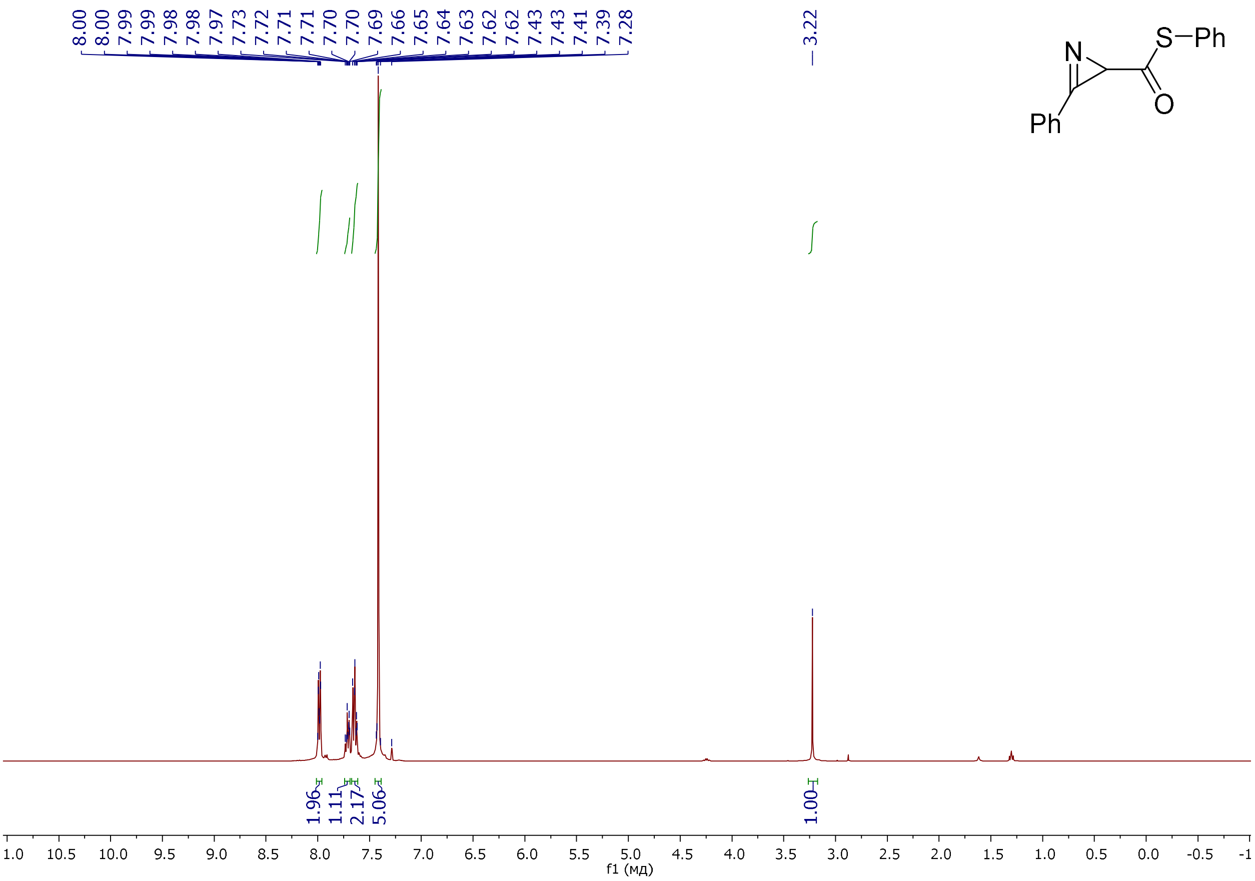

^{13}C{^{1}H} NMR spectra ($CDCl_3$, 100 MHz) of compound **15a**

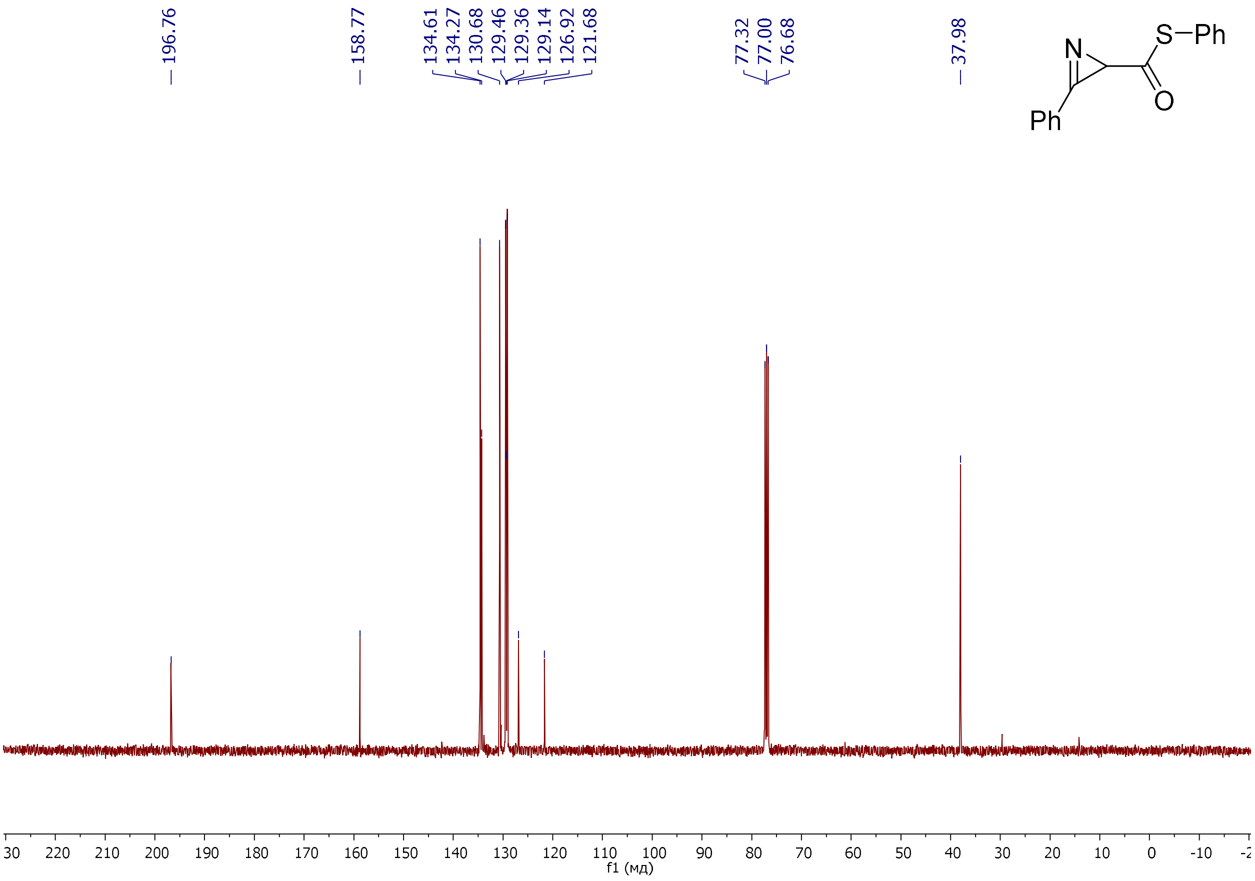

^{1}H NMR spectra ($CDCl_3$, 400 MHz) of compound **15b**

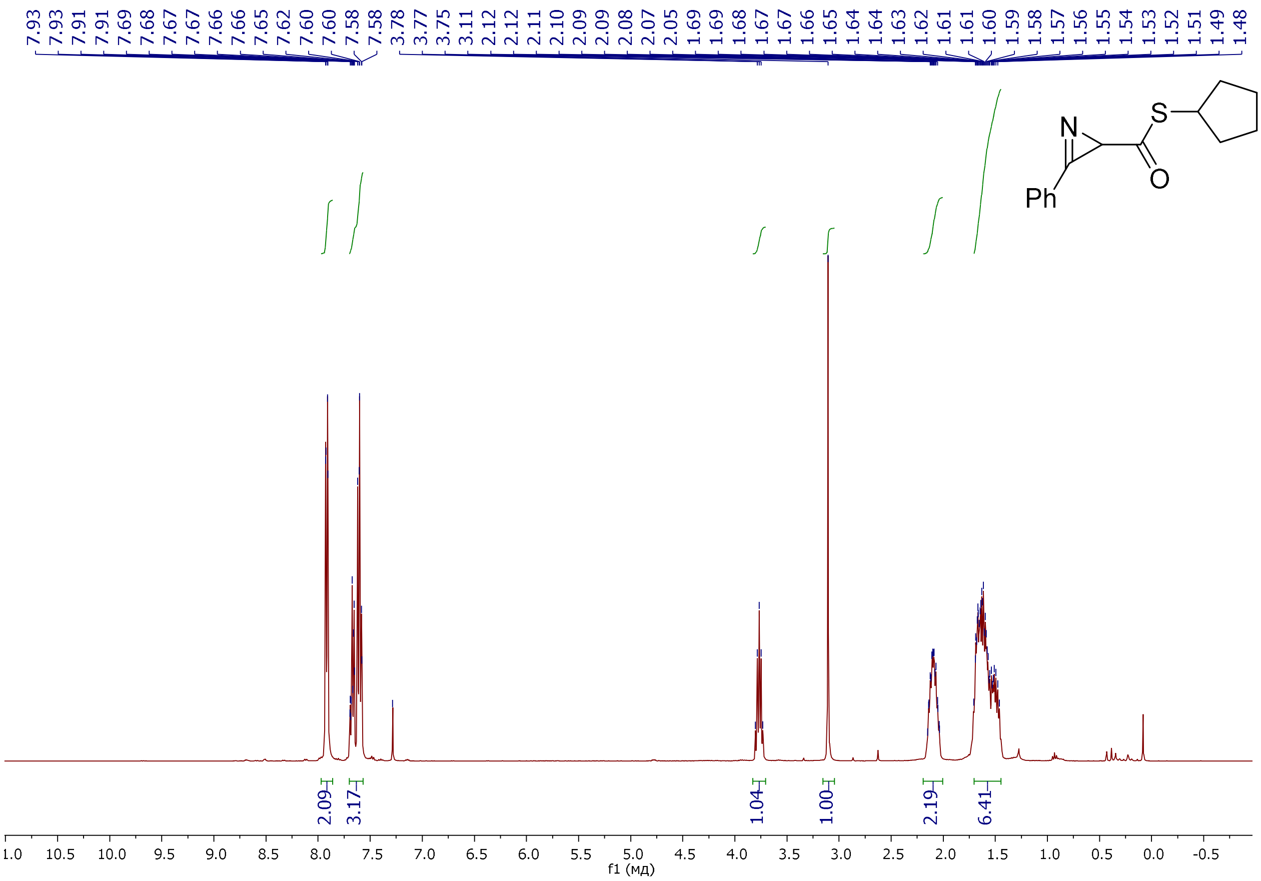

^{13}C{^{1}H} NMR spectra ($CDCl_3$, 100 MHz) of compound **15b**

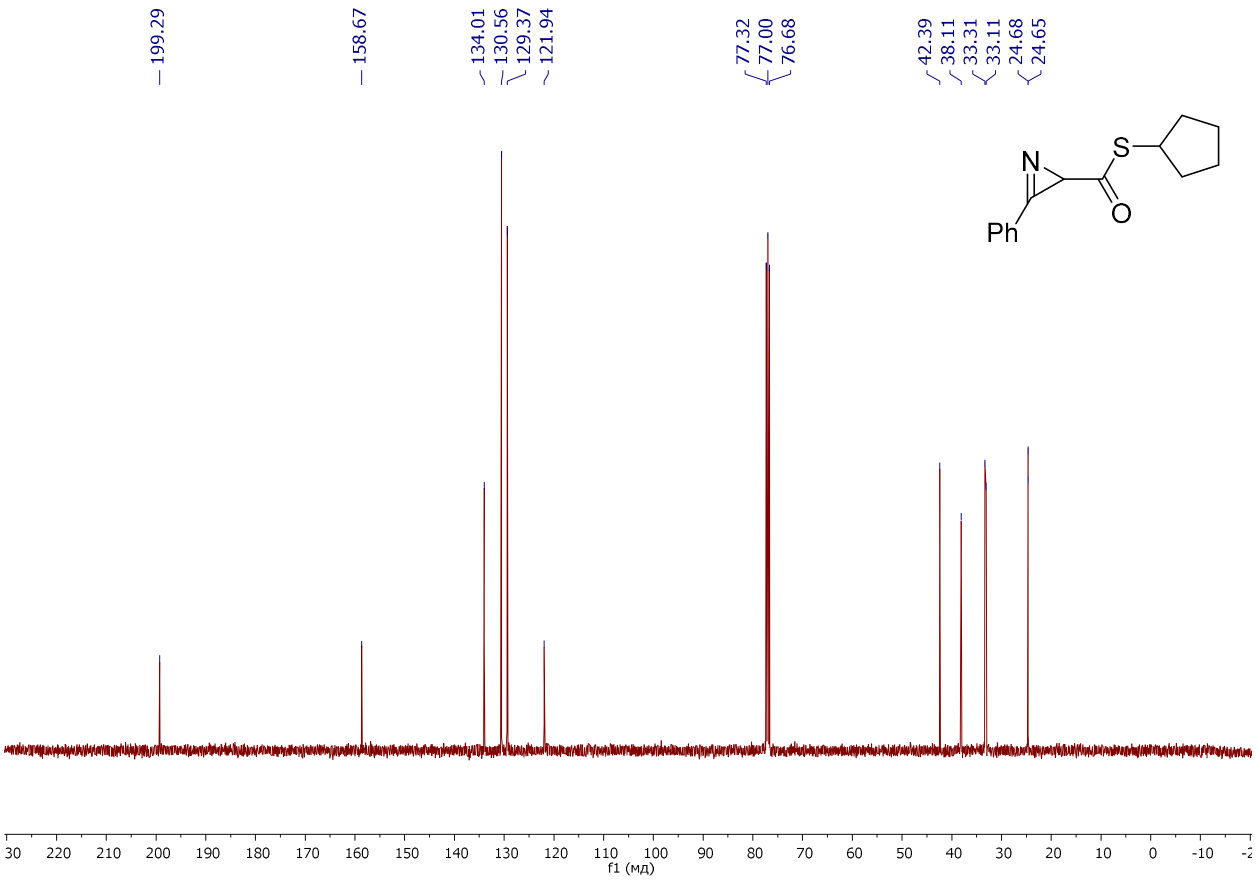

^{1}H NMR spectra ($CDCl_3$, 400 MHz) of compound **15c**

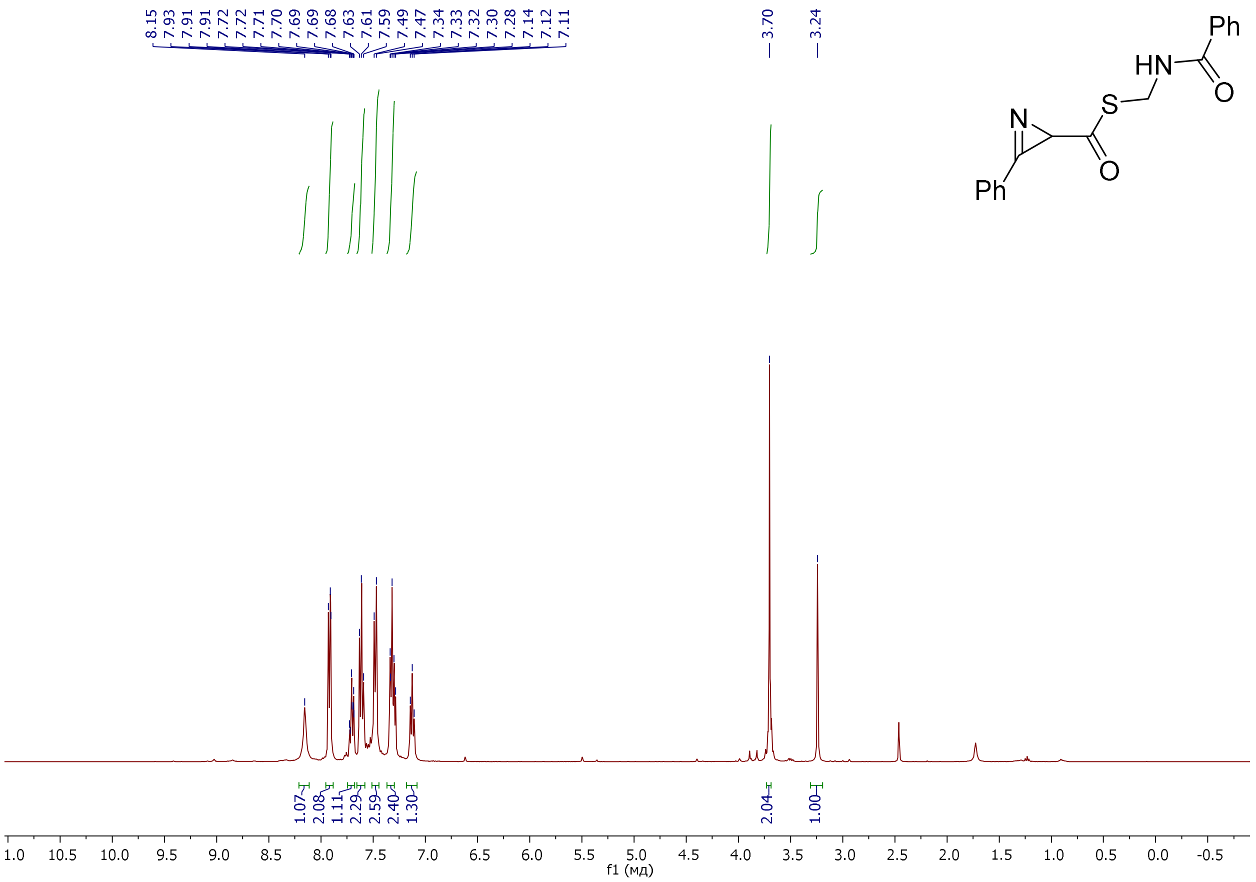

^{13}C{^{1}H} NMR spectra ($CDCl_3$, 100 MHz) of compound **15c**

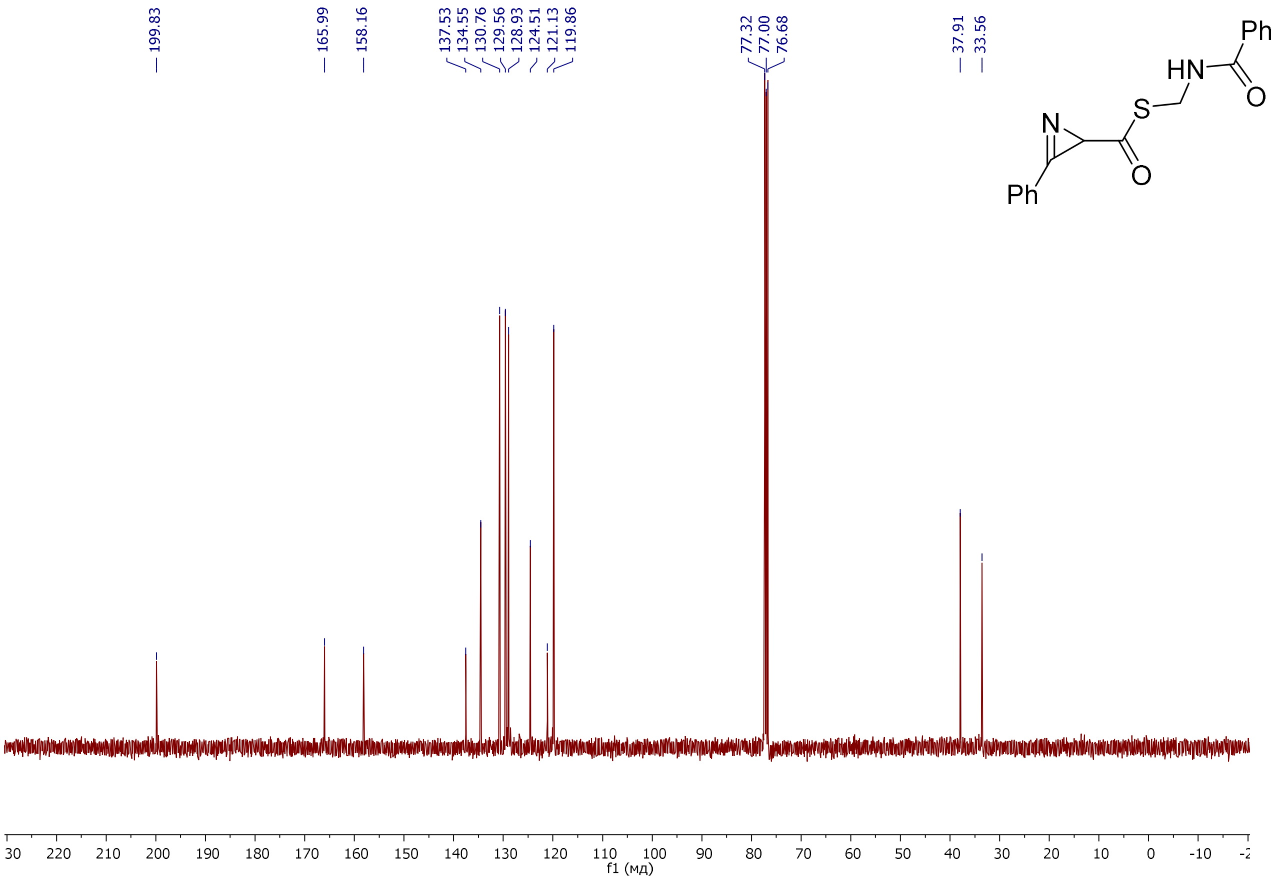

Supplement: Supplementary file 1 [file molecules-28-00275-s001.zip › molecules-2124473-supplementary.pdf]
